# Orogen styles in the East African Orogen: A review of the Neoproterozoic to Cambrian tectonic evolution^☆^

**DOI:** 10.1016/j.jafrearsci.2013.06.004

**Published:** 2013-10

**Authors:** H. Fritz, M. Abdelsalam, K.A. Ali, B. Bingen, A.S. Collins, A.R. Fowler, W. Ghebreab, C.A. Hauzenberger, P.R. Johnson, T.M. Kusky, P. Macey, S. Muhongo, R.J. Stern, G. Viola

**Affiliations:** a Department of Earth Sciences, University of Graz, 8010 Graz, Heinrichstrasse 26, Austria; b Boone Pickens School of Geology, Oklahoma State University, Noble Research Center, Stillwater, OK 74078, USA; c Faculty of Earth Sciences King Abdulaziz University, Jeddah 21589, Saudi Arabia; d Geological Survey of Norway, Trondheim, Norway; e Tectonics Resources and Exploration (TRaX), Geology and Geophysics, School of Earth andEnvironmental Sciences, The University of Adelaide, SA 5005, Australia; f Geology Department, Faculty of Sciences, United Arab Emirates University, P.O Box 17551, Al-Ain, Abu Dhabi, United Arab Emirates; g University of Asmara, Department of Earth Sciences, P.O. Box 1220, Asmara, Eritrea; h 6016 SW Haines Street, Portland, OR 97219, USA; i Three Gorges Research Center for Geohazards, State Key Laboratory of GeologicalProcesses and Mineral Resources, China University of Geosciences, Wuhan, China; j Council for Geoscience (CGS), 280 Pretoria Street, Silverton, South Africa; k Ministry of Energy and Minerals, 754/33 Samora Avenue, Dar Es Salaam,Tanzania; l Geosciences Department, University of Texas at Dallas, Richardson, TX, USA; m Norwegian University of Science and Technology, Trondheim, Norway; n University of Dar Es Salaam, Department of Geology, P.O.Box 35052, Dar Es Salaam, Tanzania

**Keywords:** East African Orogen, Mozambique Belt, Arabian–Nubian Shield, Tectonics, Metamorphism, Magmatism

## Abstract

The East African Orogen, extending from southern Israel, Sinai and Jordan in the north to Mozambique and Madagascar in the south, is the world́s largest Neoproterozoic to Cambrian orogenic complex. It comprises a collage of individual oceanic domains and continental fragments between the Archean Sahara–Congo–Kalahari Cratons in the west and Neoproterozoic India in the east. Orogen consolidation was achieved during distinct phases of orogeny between ∼850 and 550 Ma. The northern part of the orogen, the Arabian–Nubian Shield, is predominantly juvenile Neoproterozoic crust that formed in and adjacent to the Mozambique Ocean. The ocean closed during a protracted period of island-arc and microcontinent accretion between ∼850 and 620 Ma. To the south of the Arabian Nubian Shield, the Eastern Granulite–Cabo Delgado Nappe Complex of southern Kenya, Tanzania and Mozambique was an extended crust that formed adjacent to theMozambique Ocean and experienced a ∼650–620 Ma granulite-facies metamorphism. Completion of the nappe assembly around 620 Ma is defined as the East African Orogeny and was related to closure of the Mozambique Ocean. Oceans persisted after 620 Ma between East Antarctica, India, southern parts of the Congo–Tanzania–Bangweulu Cratons and the Zimbabwe–Kalahari Craton. They closed during the ∼600–500 Ma Kuungan or Malagasy Orogeny, a tectonothermal event that affected large portions of southern Tanzania, Zambia, Malawi, Mozambique, Madagascar and Antarctica. The East African and Kuungan Orogenies were followed by phases of post-orogenic extension. Early ∼600–550 Ma extension is recorded in the Arabian–Nubian Shield and the Eastern Granulite–Cabo Delgado Nappe Complex. Later ∼550–480 Ma extension affected Mozambique and southern Madagascar. Both extension phases, although diachronous,are interpreted as the result of lithospheric delamination. Along the strike of the East African Orogen, different geodynamic settings resulted in the evolution of distinctly different orogen styles. The Arabian–Nubian Shield is an accretion-type orogen comprising a stack of thin-skinned nappes resulting from the oblique convergence of bounding plates. The Eastern Granulite–Cabo Delgado Nappe Complex is interpreted as a hot- to ultra-hot orogen that evolved from a formerly extended crust. Low viscosity lower crust resisted one-sided subduction, instead a sagduction-type orogen developed. The regions of Tanzania and Madagascar affected by the Kuungan Orogeny are considered a Himalayan-type orogen composed of partly doubly thickened crust.

## Highlights

• We review the tectonic evolution of the East African Orogen.
• We present tectonic and metamorphic maps from East Africa and Madagascar.
• We define orgogen styles of East African and Kuungan orogenies.

## Graphicalabstract

Condensed timetable of major geologic events in the East African Orogen. Opening of the Mozambique Ocean and formation of rift basins in Zambia, Malawi, Mozambique and SW Madagascar was accompanied by emplacement of anorthosite melt in extending crust. A first phase of ocean closure and accretion of terranes defines the East African Orogeny. The second orogen phase, Kuungan Orogeny, mainly affected the southern parts of east Africa and Madagascar. Both shortening events were followed by extension phases that were accompanied by emplacement of late- to post-tectonic granitoids. EGCD: Eastern Granulite–Cabo Delgado Nappe Complex; Madag: Madagascar; Ub–Us: Usagaran/Ubendian Belts; WG: Western Granulite Belt; IB (Z–M): Irumide Belt of Zambia and Malawi; IB (M): Irumide Belt of Mozambique.

**Figure:**
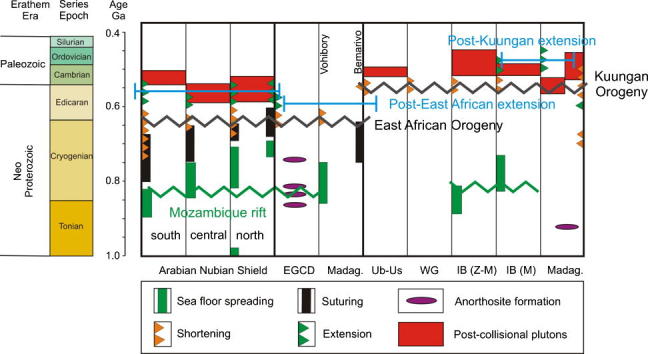

## 1 Introduction

**Fig. 1:**
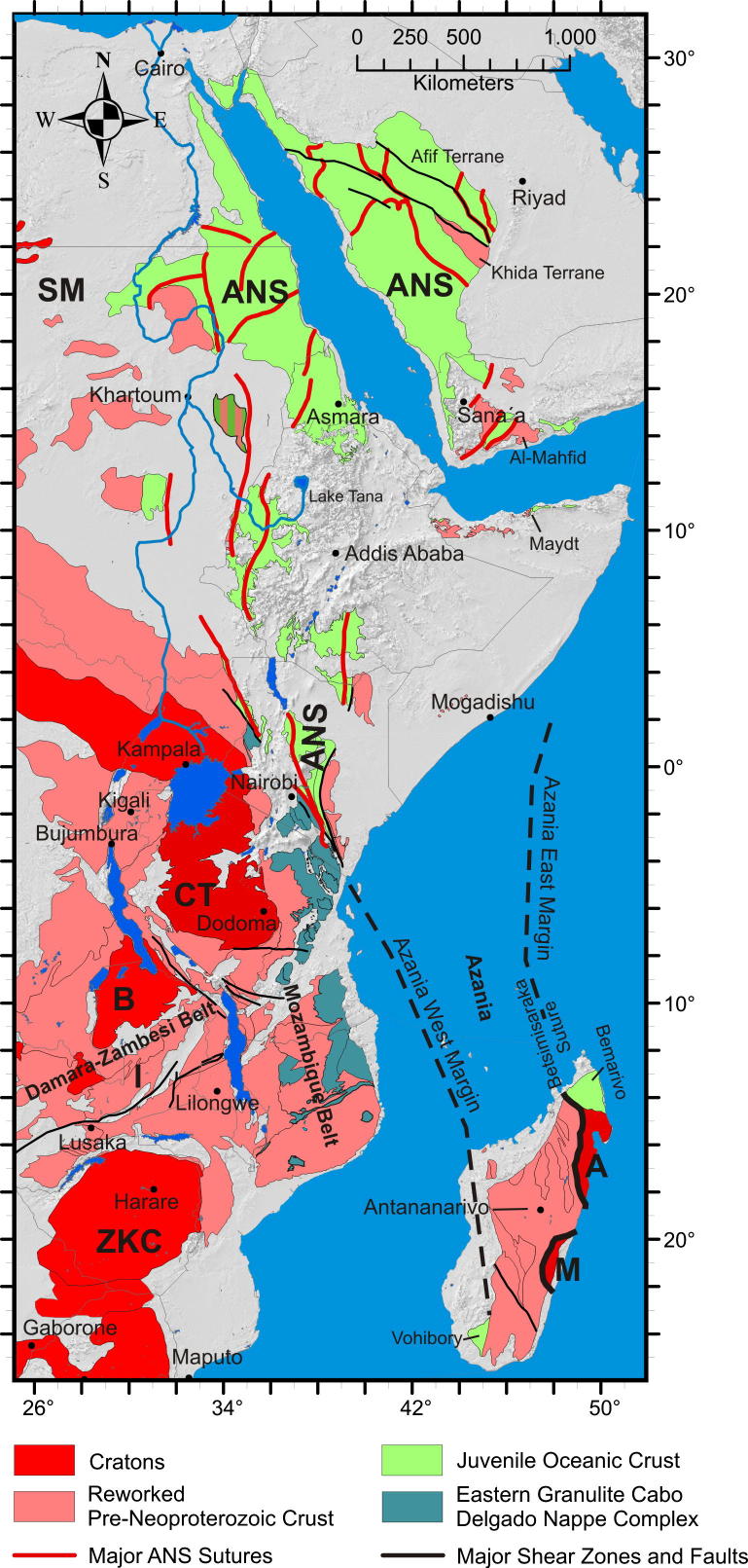
Distribution of crustal domains in the East African Orogen. SM, Sahara Metacraton; CTB, Congo–Tanzania–Bangweulu Cratons; ZKC, Zimbabwe–Kalahari Cratons; I, Irumide Belt; A, Antogil Craton; M, Masora Craton; ANS, Arabian Nubian Shield. Capital names indicated here are not labelled on subsequent maps.

The East African Orogen (EAO; Stern, 1994) is a Neoproterozoic–early Cambrian mobile belt that today extends south along eastern Africa and western Arabia from southern Israel, Sinai and Jordan in the north to Mozambique and Madagascar in the south. A southern continuation of the EAO, from Mozambique to Antarctica, was proposed by Jacobs et al., 1998, Jacobs and Thomas, 2004 and Grantham et al. (2011), but this is challenged by Collins and Pisarevsky (2005). Crust similar to the EAO is likely present in the northern Arabian plate buried under Phanerozoic sedimentary cover (Stern andJohnson, 2010), and also in terranes now found in Asia Minor. In its southern portion, between the Congo–Tanzania–Bangweulu Cratons and the Zimbabwe–Kalahari Craton (Fig. 1), the EAO devides into the E–W trending Damara–Zambesi Belt (Johnson et al., 2005) that continues to the Southern Granulite Terrane of India (Plavsa et al., 2012, Collins et al., in press) and further into Sri Lanka and East Arctica at Lützow-Holm Bay (Shiraishi et al., 1994). All these belts were part of an orogenic cycle spanning the period between breakup of Rodinia (870–800 Ma; Li et al., 2008) and final amalgamation of Gondwana (∼500 Ma: Collins and Pisarevsky, 2005, Pisarevsky et al., 2008). Here we review the tectonometamorphic evolution of the EAO. Extending ∼6000 km N–S, the EAO forms the largest continuous Neoproterozoic–Cambrian orogen on Earth, comparable in extent to the ∼7500 km long Cenozoic Alpine–Himalayan orogenic system. The EAO is considered a “Transgondwanan supermountain range” whose formation and destruction had important implications for atmospheric oxygenation (Och and Shields-Zhou, 2012) and thus for the evolution of higher organized organisms on Earth (Squire et al., 2006).

Traditionally, the EAO is subdivided into the Arabian–Nubian Shield (ANS) in the north, composed largely of juvenile Neoproterozoic crust (e.g. Stern, 1994, Stern, 2002, Johnson and Woldehaimanot, 2003, Johnson et al., 2011), and the Mozambique Belt (MB) in the south comprising mostly pre-Neoproterozoic crust with a Neoproterozoic–early Cambrian tectonothermal overprint (Fig. 1) (e.g., Fritz et al., 2005, Collins, 2006, De Waele et al., 2006a, Viola et al., 2008, Bingen et al., 2009). Although the focus of this review is on Neoproterozoic to early Cambrian mountain building and destruction processes that shaped the EAO, relevant precursor EAO orogenies are also briefly described. We avoid using the term “Pan-African” to describe the orogenies outlined below. This term was originally applied to describe a widespread ∼500 Ma tectonothermal event in Africa (Kennedy, 1964) but was subsequently used widely toencompass any Neoproterozoic thermal or tectonic event. Hence, we believe that this term has lost any use as defining a particular tectonic event. Instead, we herein refer to ∼650–620 Ma tectonothermal events as the East African Orogeny and ∼600–500 Ma events as the Kuunga Orogeny. The term Kuunga was originally used as a time frame of global orogenies (Meert et al., 1995) and subsequently linked with the collision of Australia–Antarctica with southern India and the Kalahari Craton (Meert, 2003). Collins and Pisarevsky (2005) then introduced the term Malagasy Orogeny to specify the ∼600–500 Ma orogeny caused by the collision of India with the already amalgamated Congo and Azania terranes, the latter constituting much of Madagsacar. Here we use the broader term KuungaOrogeny for a common ∼600–500 Ma tectonometamorphic event in East Africa.

The ∼6000 km length of the EAO makes it is unlikely that it has similar tectonic styles along its entire length. Even a cursory inspection suggests that different processes were active in the ANS in the north and the MB in the south. Additional differences in temporal and geometric relations are seen in Tanzania and Mozambique where the E–W trending Damara–Zambesi–Irumide Belt intersects the N–S trending MB. Thus it is appropriate to define different orogenic styles, aiming to highlight major characteristics of the EAO segments. By orogen style we understand first-ordern parameters such as relative plate movement and the thermal and mechanical structure of the crust/lithosphere during mountain growth and destruction.

## 2 Configuration of plates and oceanicbasins

The late Mesoproterozoic to Cambrian period is one of the most remarkable time episodes in Earth’s history. It began with the formation of supercontinent Rodinia through worldwide orogenic events between ∼1300 Ma and 900 Ma involving most continental blocks at that time (Cawood, 2005, Li et al., 2008). At ∼825 Ma Rodinia began breaking-up in association with widespread continental rifting (possibly triggered by uprising of global superplumes (Cawood, 2005, Li et al., 2008). By the end of the Neoproterozoic to early Cambrian the supercontinent Gondwana was formed by multiple closures of oceanic realms. A generalized picture (Fig. 1) shows three major orogens that shaped the final configuration of greater Gondwana.These include the West-African-Brasiliano Orogen, which was tectonically active until at least the end of the Cambrian (∼490 Ma; Schmitt, 2004, Meert and Lieberman, 2008), the Damara–Zambesi–Irumide Orogen, and the East African Orogen that records a polyphase tectonothermal evolution. Central Gondwana, comprising numerous terranes attached to the Sahara Metacraton (Abdelsalam et al., 2002) and parts of the Congo–Tanzania–Bangweulu Craton, underwent >∼600 Ma collisional deformation forming the East African Orogeny. However, oceans still existed between East Antarctica, India, southern parts of the Congo–Tanzania–Bangweulu Craton and the Zimbabwe–Kalahari Craton at that time (e.g., Meert, 2003, Collins and Pisarevsky, 2005). The second major orogenic episode, the KuunganOrogeny, took place between ∼600 and 500 Ma when India moved closer to its Gondwana position and remaining oceanic realms were closed.

The older, East African Orogeny resulted from the collision of amalgamated arc terranes of the ANS with the Sahara and Congo–Tanzania Cratons to the west and the Azania and Afif terranes to the east (Fig. 1). Azania, named after the ancient name for the East African coast (Collins and Pisarevsky, 2005), constitutes one or more continental blocks between the Indian Shield and Congo–Tanzania–Bangweulu Craton. It was defined by geochronological data (Collins and Windley, 2002) as an extensive ribbon or micro-continent of Archean and Paleoproterozoic crust (2900–2450 Ma), extending over Madagascar, Somalia, and Arabia (Afif terrane).The Neoarchean Al-Mahfid Block of Yemen (Fig. 1), a continental block that was thermally and structurally reworked in the Neoproterozoic–Cambrian, is the likely link between Azania and Afif (Windley et al., 1996, Whitehouse et al., 2001). As modeled by Collins and Pisarevsky (2005) and Collins et al. (2012), the Azania microcontinent separated from the Congo–Tanzania–Bangweulu Craton due to roll-back of the Mozambique Ocean subducted slab under central Madagascar, resulting in formation of an early Neoproterozoic or even latest Mesoproterozoic back-arc basin.

In the view of Collins and Pisarevsky (2005) both the eastern and western margins of Azania are marked by Neoproterozoicvolcanosedimentary sequences and rocks formed in an oceanic environment. To the east of Azania these include rocks incorporated into the Betsimisaraka Suture of eastern Madagascar, the juvenile Cryogenian rocks of the Bemarivo domain of northern Madagascar (Thomas et al., 2009) and possibly the Maydt (or Mait) Complex in northern Somalia (Fig. 1). Late Cryogenian–Ediacaran terranes define its eastern margin in Arabia (Johnson et al., 2011). The western margin of Azania in Madagascar is marked by juvenile Neoproterozoic oceanic crust exposed in the Vohibory Belt (Jöns and Schenk, 2008, Tucker et al., 2011, Collins et al., 2012). In Kenya, Ethiopia, Eritrea, Sudan, Egypt and Saudi-Arabia Neoproterozoic ophiolites and juvenile volcanic rocks are found in broad,south tapering terranes defining Azaniás western margin (e.g., Abdelsalam and Stern, 1996). The ocean separating Azania from East Africa was interpreted to have started being consumed by an intra-oceanic arc in the Tonian (Jöns and Schenk, 2008, Collins et al., 2012). This ocean is thought to have finally closed by continent–continent collision between Azania and the Congo–Tanzania Craton at ∼630 Ma (Collins and Windley, 2002, Collins, 2006) causing high-grade metamorphism and contractional deformation in southwestern Madagascar (Jöns and Schenk, 2011) and eastern Africa (Möller et al., 2000, Hauzenberger et al., 2004, Hauzenberger et al., 2007).

The Kuunganshortening phase is absent in the exposed ANS, but is likely to occur beneath the Phanerozoic of central Arabia (Cox et al., 2012, Johnson et al., 2011). This orogeny is prevalent in the southern EAO. It marks the time when Gondwana amalgamation was completed through the closure of the remaining oceanic basins, including those around Azania (Meert, 2003, Jacobs and Thomas, 2004, Collins and Pisarevsky, 2005) and the simultaneous docking of India to Australia–East Antarctica (Collins, 2003, Fitzsimons, 2003, Boger and Miller, 2004, Collins and Pisarevsky, 2005, Kelsey et al., 2008, Cox et al., 2012). In East Africa an E–W trending belt with Kuungan ages extends from Zambia–Malawi across Mozambique and further into southern India and Sri Lanka (Collins et al., 2007, Collins et al., in press, Plavsa et al., 2012). Eclogites, arc-volcanics and ophiolites from the Irumide–Zambezi Belt (Fig. 1) demonstrate that Mesoproterozoic to Neoproterozoic oceanic crust existed between the Congo–Tanzania–Bangweulu Craton and the Zimbabwe–Kalahari Craton (John et al., 2003). Closure of this ocean and collision of the Zimbabwe–Kalahari Craton with the Congo–Tanzania–Bangweulu Craton has been interpreted to correlate with 560–510 Ma high-pressure metamorphism found in Zambia, southern Tanzania (Boniface and Schenk, 2012) and Malawi (Ring et al., 2002). Peak-metamorphism in the Damara Belt dated at 538–505 Ma (Goscombe etal., 2004) may reflect amalgamation of the Congo and Kalahari Cratons farther west.

Simultaneously, or shortly after the East African Orogeny, a series of Cadomian terranes such as Avalonia and Armorica were rifted off the northern margin of Gondwana in the early Paleozoic and accreted to Laurentia (Keppie et al., 2003). Rifting of these terranes, possibly due to the retreat of the Cadomian arc (Jacobs and Thomas, 2004; Meert and Lieberman, 2008), may have enabled northward expulsion of the ANS. Soon after the Kuungan collisional phase, rifting of terranes around the Natal embayment between South Africa and Dronning Maud Land of East Antarctica enabled late southward extrusion of southern African domains (Jacobs and Thomas, 2004).

## 3 Crustal domains within the East African Orogen

### 3.1 The Arabian–Nubian Shield

Although pre-Neoproterozoic crust crops out in the ANS, there is no evidence of extensive pre-Neoproterozoic crust beneath the shield. A critical review of geochronological and isotopic data from both the northern ANS (Saudi Arabia, Egypt, Sudan: Stern and Kröner, 1993, Stern, 2002, Stern et al., 2010a, Stein, 2003, Stoeser and Frost, 2006, Ali et al., 2009, Ali et al., 2012, Andresen et al., 2009, Liégeois and Stern, 2010, Johnson et al., 2011) and the southern ANS (Eritrea, Ethiopia, Kenya: Teklay et al., 1998, Stern, 2002, Yibas et al., 2002, Kebede and Koeberl, 2003, Stern et al., 2010b) shows that the internal portions of the ANS are dominantly composed of juvenile Neoproterozoic crust. Stern et al. (2010a) have shown that only ∼5% of individually dated zircons from the ANS igneous rocks are older than 880 Ma, with concentrations in the Tonain-Stenian (0.9–1.15 Ga), late Paleoproterozoic (1.7–2.1 Ga), Paleoproterozoic–Neoarchean (2.4–2.8 Ga) and early Archean (>3.2 Ga). Stern et al. (2010a) favored the interpretation that these zircon xenocrysts resided in the mantle and were entrained when this mantle melted to form juvenile ANS crust. The largest contiguous fragment of pre-Neoproterozoic crust is represented by the isotopically defined 1800–1650 Ma old Khida terrane in Arabia (Stoeser and Frost, 2006, Johnson et al., 2011) (Fig. 1). The juvenile crust around and to the west of this terrane contains minor assimilation of continental material and has been defined as “contaminated shield” (Hargrove et al., 2006a).

**Fig. 2:**
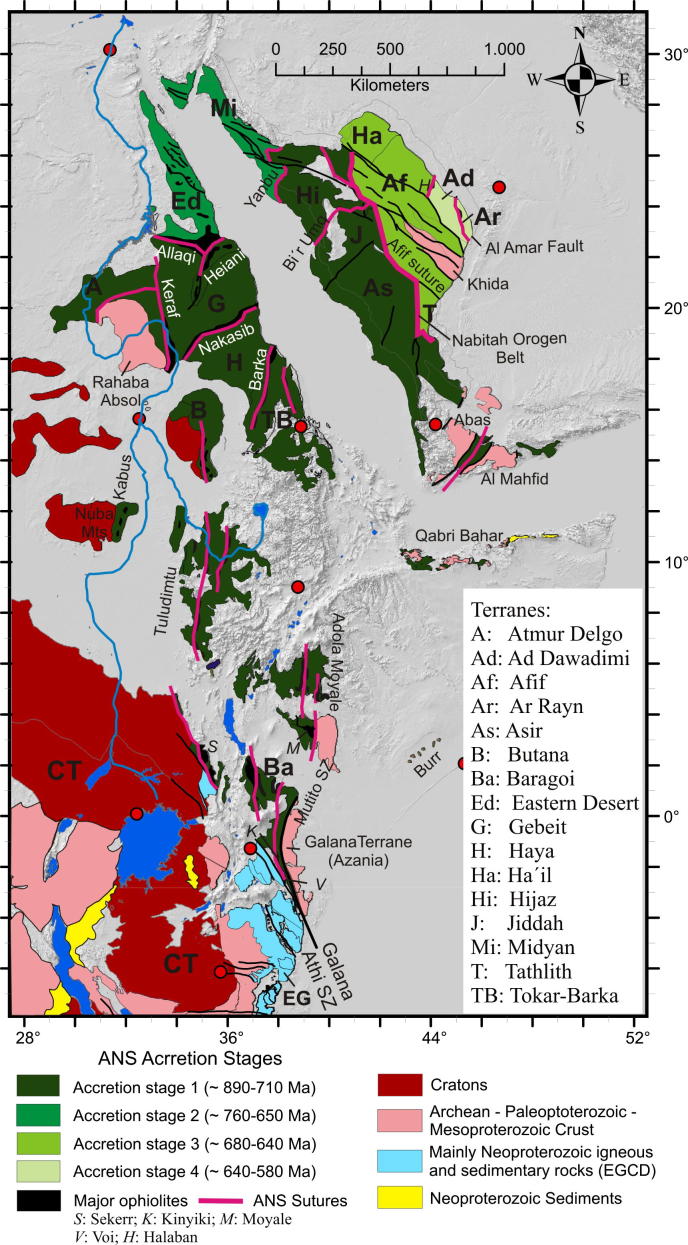
Crustal age domains in the northern East African Orogen and crustal growth phases in the Arabian–Nubian Shield. CT, Congo–Tanzania Craton; EG, Eastern Granulites.

The ANS constitutes a southward narrowing belt internally structured by individual terranes (Fig. 1, Fig. 2). Its western margin is defined by juxtaposition of ophiolite-decorated volcanosedimentary sequences and juvenile Neoproterozoic arc magmatic terranes with the Eastern Granulite complex of the MB, the Archean Congo Craton and the Sahara Metacraton (Fig. 1). In the north, the westmargin of the ANS is not defined because it is covered by Mesozoic to Cenozoic sedimentary rocks but it extends along the line of the Nile Valley and crops out in the Keraf arc-continent suture in northern Sudan (Abdelsalam et al., 1998). South of this, the margin is defined by a line of sutures and ophiolite belts, namely the Kabus suture of the Nuba Mountains (Abdelsalam and Dawoud, 1991), the Sekerr ophiolite of northwestern Kenya (Vearncombe, 1983, Berhe, 1990, Ries et al., 1992, Mosley, 1993) and the Kinyiki ophiolite of southern Kenya (Frisch and Pohl, 1986) (Fig. 2). The southern tip of the ANS is represented by a belt of 955–845 Ma old subduction-related amphibolites andgneisses adjacent to the Galana-Athi shear zone (Fig. 2; Hauzenberger et al., 2007, Bauernhofer et al., 2009). The same shear zone defines the western margin of the Galana terrane considered to be part of Azania.

The eastern ANS margin, the inferred contact with Azania, is not well-defined in Kenya, Ethiopia and Somalia. However, we propose that it is marked by the Mutito-Bruna Shear Zone (Mosley, 1993), which separates the Barsaloi-Adola Moyale ophiolitic belts from the poorly exposed older suites of the Burr Complex in southern Somalia (Fig. 2; Warden and Horkel, 1984). The Qabri Bahar and Mora Complexes (Fig. 2) containingpre-Neoproterozoic zircons (Kröner and Sassi, 1996) may represent Azania in northern Somali. The ANS-Azania boundary in Yemen is probably defined by arc-continent sutures along the Abas and Al Mahfid terranes (Fig. 2). The Afif terrane in eastern Saudi Arabia, including the Paleoproterozoic Khida subterrane, likely represents a crustal block within the ANS since its boundaries are defined as arc–arc sutures (Whitehouse et al., 2001, Johnson et al., 2011).

#### 3.1.1 Crustal growth phases of the Arabian–Nubian Shield

Apart from a few ages dating the formation of oceanic crust, such as data from ophiolite suites including leucogranite (e.g., Pallister et al., 1988, Stern et al., 1991, Kröner et al., 1992, Andresen et al., 2009), the overwhelming majority of geochronological data in the ANS came from granitoids. A rather oversimplified but generally accepted opinion is that the early magmatic calc-alkaline granitoid bodies formed within evolving arc settings (e.g., Hargrove et al., 2006a, Hargrove et al., 2006b). Differently, the youngest late- to post-collisional alkaline suite granitoids are thought to have formed during the decay of the previously consolidated orogen, possibly involving sub-continental lithospheric delamination (e.g., Farahat et al., 2007, Be’eri-Shlevin et al., 2009, Johnson et al., 2011). In this hypothesis, the oldest available age data of calc-alkaline magmatism reflect onset of subduction between individual terranes that finally amalgamated along the various ophiolite-decorated sutures.The distribution of protolith ages suggests that the ANS evolved through four main phases of crustal growth.

**Fig. 3:**
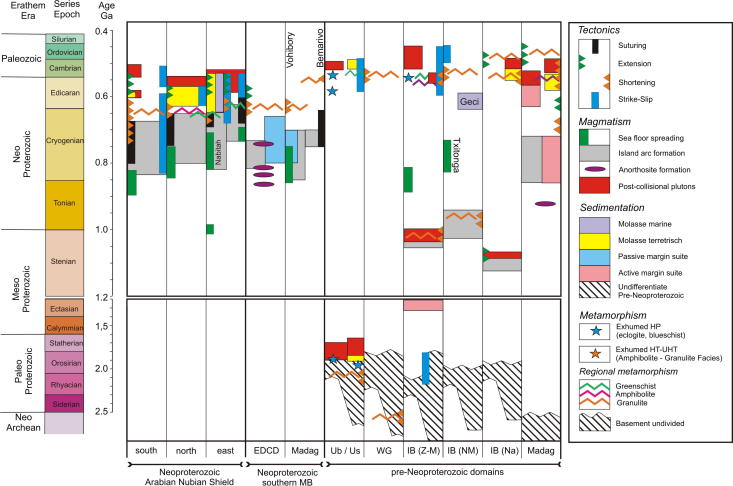
Timetable of geologic events in the East African Orogen and neighbouring areas. Time scale according to Gradstein et al. (2004). Sources are cited in the text. EGCD: Eastern Granulite–Cabo Delgado Nappe Complex; Ub/Us: Usagaran/Ubendian Belts; WG: Western Granulite Belt; IB (Z–M): Irumide Belt of Zambia and Malawi; IB (NM): Irumide Belt of northern Mozambique; IB (Na): Irumide Belt of the Nampula Block; Madag: Madagascar.
(2) The northern ANS formed during a second growth phase between ∼760 and 730 Ma when the Midyan-Eastern Desert terrane was formed. This terrane subsequently collided and amalgamated with the earlier formed older terranes along the Yanbu-Onib-Sol Hamed-Gerf-Allaqi-Heiani suture (Ali et al., 2010a, Ali et al., 2010b, Johnson et al., 2011). The resulting geologic entity is commonly referred to as the “western arc or oceanic terranes” of the ANS (e.g., Stoeser and Frost, 2006, Ali et al., 2009, Johnson et al., 2011). However, the northernmost ANS in Sinai contains also older rocks (∼1025 Ma rocks of the Sa’al Complex; Be’eri-Shlevin et al., 2012).
(3) The “western arc or oceanic terranes” of the ANS subsequently collided and amalgamated between 680 and 640 Ma with the Afif and Tathlith terranes, creating a neocontinental crustal block referred to as the proto-ANS (Johnson et al., 2011). The Afif terrane is a composite tectonostratigraphic unit comprising the Paleproterozoic Khida terrane (or subterrane) and three Neoproterozoic arc assemblages formed at 840–820 Ma, 750–720 Ma, and 700–680 Ma. The 680–640 Ma assembly of the proto-Arabian–Nubian Shield was associated with metamorphic, deformational, and igneous intrusion events that are referred to in Arabia to as the “Nabitah orogeny” (680–640 Ma; Stoeser and Stacey, 1988).
(4) The youngest ANS terranes, with late Cryogenian to Ediacaran protoliths, are the Ad Dawadimi and Ar Rayn terranes in the easternmost part of the ANS in Arabia (Fig. 2, Fig. 3). A suturing event along the eastern margin of the Afif terrane is marked by the formation of the Halaban ophiolite at ∼680–670 Ma (Al-Saleh et al., 1998). Ad Dawadimi and Ar Rayn terranes are in contact along the serpentinite-decorated Al Amar fault, which is interpreted as a suture. This suture can be traced magnetically in the subsurface far to the north and is known as the Central Arabian Magnetic Anomaly. The anomaly may mark the eastern limit of the ANS (Stern and Johnson, 2010), but this is controversial and even younger sutures may lie farther east(Cox et al., 2012). The pre-Phanerozoic crust in eastern Arabia is concealed by Phanerozoic sediments but, where locally exposed in Oman, it appears to have stabilized in the Cryognenian (∼750–700 Ma) (Stern and Johnson, 2010).

(1) The southern ANS, extending from southern Kenya to the Nakasib-Bir Umq suture (Fig. 2, Fig. 3), formed first (Johnson et al., 2003). This part of the ANS constitutes terranes that are internally partitioned by arc–arc sutures (Kröner et al., 1991, Abdelsalam and Stern, 1996, Stern et al., 2010b, Johnson et al., 2011). The terranes are known as Tokar–Barka, Butana, Haya terranes in Eritrea, Ethiopia, and Sudan and Abbas, Asir, Jiddah, and Hijaz terranes in Yemen and Saudi Arabia. Protolith ages from these terranes extend back to 900–830 Ma. The tectonic evolution of the southern ANS through a Wilson Cycle is well illustrated in the Tuludimtu belt of central Ethiopia (Woldemichael et al., 2010). Here, early rifting was initiated between 900 and 860 Ma; the transition from rifting to ocean floor spreading occurred between 860 and 830 Ma; subduction and formation of arc- and back-arc basins occurred between 830 and 750 Ma; and basin closure by accretion of island arcs commenced between 750 and 650 Ma. To the north, amalgamation of arc systems by closure of internal arc–arc sutures (e.g., Barka and Bir’ Umq-Nakasib sutures) occurred between 800 and 700 Ma (Abdelsalam and Stern, 1996, Yibas et al., 2002, Woldemichael et al., 2010). The central ANS, lying between the Nakasib-Bir Umq suture in the south and the Yanbu-Onib-Sol Hamed-Gerf-Allaqi-Heiani suture in the north (Fig. 2), was formed by arc formationand accretion between 830 and 710 Ma (e.g., Johnson et al., 2003, Johnson et al., 2011). The ages for the central ANS igneous rocks overlap with those obtained from the southern ANS but also extend to younger ages.

### 3.2 The central and southern Mozambique Belt in Africa – juvenile Neoproterozoic crustal fragments

**Fig. 4:**
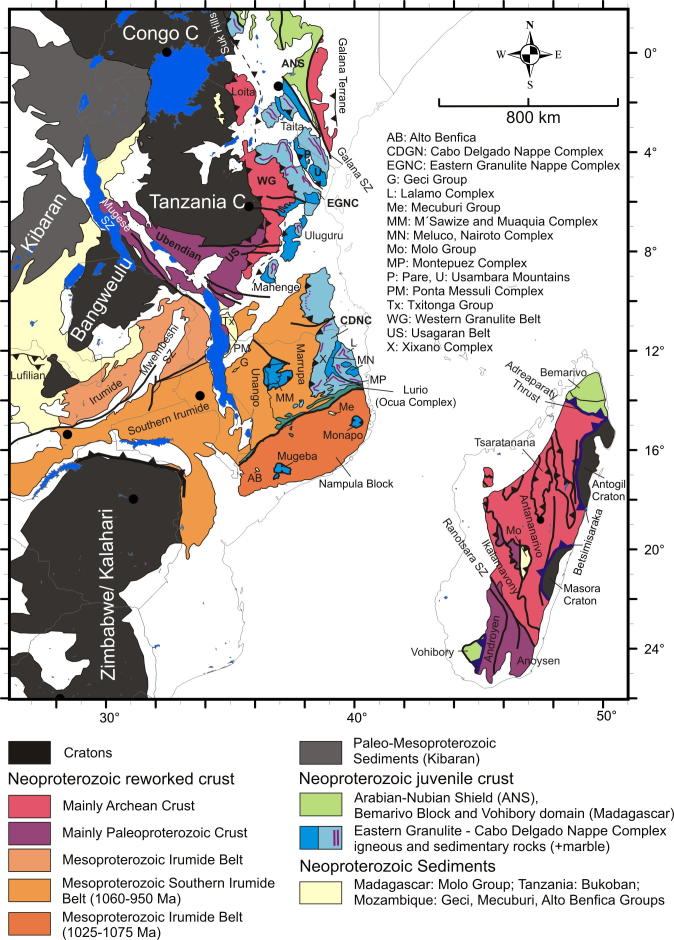
Crustal age domains and major litho-tectonic units in the southern East African Orogen.

Assemblages with high-grade Neoproterozoic metamorphism and intense deformation constitute the southward continuation of the ANS from Kenya through Tanzania to Mozambique. These units have been classified as part of the MB (Holmes, 1951) but contain a collage of different crustal fragments. A key tectonic position is occupied by the Eastern Granulites in Tanzania (Hepworth, 1972) and the Cabo Delgado Nappe Complex in Mozambique (Viola et al., 2008) that are regarded as a coherent entity (Eastern Granulite–Cabo Delgado Nappe Complex: EGCD). Recent mapping and map compilation confirm earlier observations by Mosley (1993) that this granulite belt forms a contiguous band traceable from eastern Uganda (Mäkitie et al., 2011, Mänttäri et al., 2011, Schenk and Loose, 2011), via western Kenya in the Suk Hills (Mosley, 1993), southern Kenya (Turoka Series and Taita Hills; Hauzenberger et al., 2007), eastern Tanzania (Pare-Usambara, Uluguru Mountains, Mahenge Mountains; Fritz et al., 2005) to southern Tanzania (Kröner et al., 2003) (Fig. 1, Fig. 4).

In Mozambique, this tectonic unit is subdivided into the CaboDelgado Nappe Complex (*sensu stricto)* in the north (Viola et al., 2008, Bingen et al., 2009) and the Mugeba and Monapo klippen in the Nampula Block in the center (Macey et al., 2010). In Tanzania, the Eastern Granulites are divided into a basal unit largely composed of metaigneous rock suites and an upper unit with metasedimentary rocks including marbles (Fritz et al., 2005, Fritz et al., 2009). In the Taita Hills of southern Kenya equivalent units are mapped as metamagmatic Kasigau Group and metasedimentary Kurase Group (Horkel et al., 1979, Pohl and Niedermayr, 1979, Hauzenberger et al., 2007). The metaigneous suite of Tanzania contains anorthosites as a characteristic member that yields overwhelmingly 900–700 Maformation ages (Tenczer et al., 2006). Nd-model ages around 1000 Ma (Möller et al., 1998) likewise suggest that most metaigneous rocks of the Eastern Granulites were formed during the Neoproterozoic. On the basis of these results, Maboko and Nakamura (2002) inferred that the Eastern Granulites of northern Tanzania represent Neoproterozoic juvenile crust coeval with the ANS.

The Cabo Delgado Nappe Complex of northern Mozambique is a similar stack of thrusts composed of metaigneous and metasedimentary rocks. The M’Sawize, Muaquia, Meluco and Nairoto nappes (Fig. 4; Viola et al., 2008) expose mainly felsic orthogneisses (intruded between 973 and 946 Ma) aswell as enderbite. The core of the Xixano nappe is made up of enderbite intruded at ∼744 Ma, with zircons yielding positive initial epsilon Hf values of +4.4 to +9.8 that point to a dominantly juvenile source (Bingen et al., 2007, Boyd et al., 2010). The enderbite is associated with a sequence of metavolcanic and metaplutonic rocks formed between ∼818 and ∼787 Ma.

It should be noted, of course, that not all igneous rocks of the EGCD display juvenile Neoproterozoic isotopic signatures. Some anorthosites and gneisses from the Eastern Granulite Belt of Tanzania contain Archean and Paleoproterozoic, but not Mesoproterozoic zircons (Muhongo et al., 2001, Muhongo et al., 2003, Tenczer et al., 2006, Le Goff et al., 2010). In Mozambique, parts of the M’Sawize, Muaquia, Meluco and Nairoto nappes contain basementgneisses with ages overlapping those of the underlying Mesoproterozoic Marrupa and Unango complexes (Boyd et al., 2010). It is uncertain whether these rocks represent remnants of original pre-Neoproterozoic crust or tectonically emplaced thrust slices, but otherwise, overall, the EGCD is considered to be “juvenile crust” in that it began forming from ∼900 Ma onward, similar to ANS crust.

A distinctive feature of the EGCD is the presence of marble-bearing metasedimentary units. Depositional ages of eight occurrences of marble exposed in the Cabo Delgado Nappe Complex of Mozambique (Xixano-, Lalamo- and Montepuez-Complexes) have been narrowed down by chemostratigrapic ^87^Sr/^86^Sr isotopic studies between 800 and 600 Ma (Melezhik et al., 2008). The time range of sedimentation, aftercessation of Mesoproterozoic orogenies in Mozambique and before onset of the Mozambique orogeny, makes deposition on the margin of the Mozambique Ocean plausible. Presumably the sediments were deposited in a passive continental margin setting in the ocean separating Azania from mainland Africa, as also inferred from scanty evidence by Stern (1994). This passive margin may have formed when Azania rifted off from the Congo–Tanzania–Bangweulu craton in the early Neoproterozoic (Collins and Pisarevsky, 2005). Alternatively, the Xixano enderbite nappe and the metasedimentary Lalamo nappe have been interpreted as fragments of oceanic volcanic arcs formed within the Mozambique Ocean (Boyd et al., 2010, Bingen et al., 2011).

### 3.3 The Mozambique Belt in Madagascar – juvenile Neoproterozoic crustal fragments

The Bemarivo Belt of northern Madagascar and the Vohibory domain in the southwest (Fig. 1, Fig. 3, Fig. 4) consist mainly of juvenile Neoproterozoic rocks (Jöns and Schenk, 2008, Thomas et al., 2009); all other domains constitute pre-Neoproterozoic crust. The Bemarivo Belt itself is divided into two terranes (Thomas et al., 2009). The older, in the south, contains high-grade paragneisses derived from Paleoproterozoic protoliths; the younger northern terrane is a Cryogenian arc system developed to the east of Azania. This arc system comprises volcanosedimentary sequences with a maximum depositional age of ∼750 Ma and 740–750 Ma magmatic rocks (Jöns et al., 2006) and has been linked with the Seychelles and north-west India because of the common occurrence of ∼750 Ma magmatic rocks (Tucker et al., 2001, Torsvik et al., 2001, Ashwal et al., 2002).

The Vohibory domain is the westernmost of three domains in southern Madagascar, south of the sinistral Ranotsara (shear)zone (Fig. 4; Schreurs et al., 2010). The other domains are the Androyen and Anosyen, and all are separated by N-trending shear zones (Martelat et al., 2000). The Vohibory domain consists of juvenile mafic granulites and metasediments with protolith ages between 910 and 760 Ma (Jöns and Schenk, 2008, Collins et al., 2012). It has been interpreted as an intra-oceanic arc within a Neoproterozoic ocean west of the reworked older crust of central Madagascar. The intra-oceanic character of the domain and its 630–600 Ma peak metamorphism (Jöns and Schenk, 2008), similar to that of the Eastern Granulites in Tanzania (Möller et al., 2000, Hauzenberger et al., 2007), are strong evidence that juvenile crust extends from southern Kenya and Tanzania to southern Madagascar. Correlation between the Vohibory domain and the Eastern Granulites is even more evident when Madagascar is restored to its position before rifting-off East Africa.

Accepting that the Bemarivo Belt and the Betsimisaraka Suture as well as the Vohibory Belt contain Neoproterozoic juvenile crust implies that Archean Madagascar, the core of Azania, was flanked by two oceans (Fig. 1). The western Mozambique Ocean can be traced from the Vohibory domain to the ANS. The eastern Malagasy Ocean continued from the Betsimisaraka Suture via the Bemarivo Belt to the Seychelles (Collins and Pisarevsky, 2005) and, more controversially, into Arabia (Cox et al., 2012).

### 3.4 The central and southern Mozambique Belt in East Africa – reworked crustal fragments

In the central and southern MB heterogeneous pre-Neoproterozoic crustal fragments have been variably reworked during both the East African and Kuungan orogenies. The western MB orogenic front, as defined by the first appearance of Neoproterozoic and/or early Cambrian deformation andmetamorphism, can be approximately delineated by the eastern margins of the Congo–Tanzania–Bangweulu Craton (Kabete et al., 2012a, Kabete et al., 2012b) and the Zimbabwe–Kalahari Craton. Major crustal blocks with distinctly different pre-Neoproterozoic tectonothermal evolutions are grouped here as the Usagaran–Ubendian Belts, the Western Granulite Belt, the Irumide Belt and the Nampula Block (Fig. 3, Fig. 4). Neoproterozoic to Early Cambrian deformation and metamorphism, subject of subsequent chapters, is variable in these units.

#### 3.4.1 The Usagaran–Ubendian Belts

Basal and westernmost (external) portions of the nappe pile within the MB of Tanzania, northern Malawi and Zambia constitute the Usagaran Belt to the east of the Tanzania Craton and the Ubendian Belt to thesouth and southwest of it (Fig. 4). Both are the product of Paleoproterzoic orogeny and have similar Paleoproterozoic magmatic ages (2000–1800 Ma; Gabert and Wendt, 1974, Lenoir et al., 1994, Möller et al., 1995, Collins et al., 2004), strong evidence that the two are correlative. The belts mostly lack Mesoproterozoic (Kibaran) units, but parts of the Ubendian Belt contain 1090–1120 Ma granite plutons (Ring et al., 1999) and rocks metamorphosed at 1091 Ma (Boniface et al., 2012).

The Ubendian Belt is a transcurrent NW-trending orogen in which different terranes were juxtaposed by repeated strike-slip deformation (Priem et al., 1979, Theunissen et al., 1996, Boven et al., 1999). The belt is characterized by an early granulite-facies metamorphism dated at 2100–2025 Ma (Dodson et al., 1975, Lenoir et al., 1994, Ring et al., 1997, Muhongo et al., 2002). The Paleoproterozoic orogenic cycle here was associated with 1831–1817 Ma subduction-type metamorphism in which eclogites have REE patterns that are similar to those of N-MORB and E-MORB (Boniface et al., 2012). Amphibolite-facies metamorphism, together with dextral strike-slip shearing and emplacement of late kinematic granitoids, occurred between 1960 and 1800 Ma (Priem et al., 1979, Theunissen et al., 1996, Vrana et al., 2004).

The Usagaran Belt is subdivided intotwo major litho-tectonic units; a high-grade structural basement (Isimani Suite: Mruma, 1989) and, separated by an angular unconformity, a ∼1920 Ma low-grade metamorphosed volcanosedimentary cover (Ndembera Group: Mruma, 1995, Sommer et al., 2005a, Tenczer et al., 2007). The Isimani Suite contains lenticular bodies of ∼2000 Ma eclogites (Möller et al., 1995, Reddy et al., 2003, Collins et al., 2004). Depleted mantle Nd model ages of 3100–2800 Ma obtained from Usagaran gneisses (Maboko and Nakamura, 1996, Möller et al., 1998, Maboko, 2000) suggest a component of Archean crust. More than 50% of the total exposure of the belt consists of granitoids and granitoid gneiss. They wereemplaced between 1900 and 1730 Ma (Gabert and Wendt, 1974, Maboko and Nakamura, 1996, Sommer et al., 2005a) and represent syn- to post-tectonic intrusions (Gabert, 1973).

#### 3.4.2 The Western Granulite Belt of Tanzania

The Western Granulite Belt (Fritz et al., 2005) or Western MB (Cutten et al., 2006) forms a contiguous tectonic unit that rests structurally above the Tanzania Craton and/or the Usagaran Belt and structurally below the Eastern Granulites (Fig. 4). The belt is traceable from southwestern Kenya (Loita Hills: Mosley, 1993) to southern Tanzania. Its southern termination is not well defined, largely because it is poorly exposed within the Selous Game Reserve and covered by late Paleozoic–Mesozoic Karoo sediments (Wopfner, 2002) and rift-related Neogene sedimentary rocks. Because few geochemical and geochronological data exist from the Western Granulite Belt, the nature of its crustal assembly remains speculative. Even the name “Western Granulites” is a bad choice since many rocks in this unit experienced much lower grade of metamorphism. However, Nd model ages of 2500–3100 Ma, indistinguishable from those obtained from the Tanzanian Craton, suggest a predominance of reworked Archean crust (Maboko, 1995, Möller et al., 1998), and zircon geochronology indicates granitoid emplacement in the Archean and the Paleoproterozoic (Muhongo et al., 2001, Johnson et al., 2003, Sommer et al., 2003, Sommer et al., 2005b, Cutten et al., 2006, Vogt et al., 2006, Le Goff et al., 2010, Tenczer et al., 2012, Thomas et al., 2013).

#### 3.4.3 The Irumide Belt in Zambia and Malawi

The Irumide Belt is a NE-trending Mesoproterozoic orogenic belt stretching from Zambia via northern Malawi to southern Tanzania (Fig. 4; De Waele et al., 2006a). Mesoproterozoic and Neoproterozoic transcurrent shear zones (Mugese Shear Zone) separate the Irumide Belt from reactivated parts of the Paleoproterozoic Ubendian Belt in the northeast. Its continuation into southern Tanzania remains to be established. Although not considered part of thegeographically defined Irumide Belt, large portions of Mozambique (Marrupa, Unango Complexes and Nampula Block) are also composed of Mesoproterozoic crust that may be part of the Irumide Belt. Hence, large portions of the southern MB are likely floored by Mesoproterozoic crust.

An extensive geochronological and structural data base allows detailed reconstruction of tectonothermal events within the Zambian–Malawian Irumide Belt (Kröner et al., 1997, Kröner et al., 2001, De Waele and Mapani, 2002, Johnson et al., 2005, Johnson et al., 2006, De Waele et al., 2006a, De Waele et al., 2006b, De Waele et al., 2008, De Waele et al., 2009). The southern Irumide Belt (SIB) is a structurally and metamorphically complex region of mainly Mesoproterozoic igneous rocks. It is underlain by predominantly late Paleoproterozoiccrust, which was intruded by voluminous 1090–1040 Ma continental arc-related magmatic rocks, accompanied byhigh-temperature/low-pressure metamorphism (Johnson et al., 2006). The Mwembeshi Shear Zone and a Permo-Triassic graben form the contact between the SIB and the Irumide Belt *sensu stricto* (IB) (Fig. 4). The northern IB is underlain by a late Paleoproterozoic crust that is generally older than 2000 Ma and contains significant mid-Mesoproterozoic plutonic rocks that are not present within the SIB.

We draw attention to the fact that Neoproterozoic divergence along the southern margin of the Congo–Tanzania–Bangweulu craton is recorded within the Zambesi–Lufilian Belt to the northwest of the IB (Fig. 4). This belt contains rift-related volcanosedimentary passive margin sequences. A first phase of rifting between 880 and 820 Ma coincided with thedeposition of extensive copper-bearing Roan strata of the Lufilian Belt. A second phase, which began at ∼765 Ma, is interpreted as resulting in the development of an extensive Neoproterozoic passive margin (De Waele et al., 2008).

#### 3.4.4 The Irumide Belt in northern Mozambique

Northern and central Mozambique consists of two major Mesoproterozoic domains that are separated by the prominent Neoproterozoic–early Palaezoic Lurio Shear Belt (Fig. 4) (Engvik et al., 2007, Viola et al., 2008). The northern domain consists of the Unango and Marrupa complexes and the structurally overlying Cabo Delgado Nappe Complex. The southern domain, the Nampula Block, comprises Mesoproterozoic rocks tectonically below the Monapo-Mugeba klippen (Fig. 4; Viola et al., 2008, Macey et al., 2010).

The Unango and Marrupa Complexes themselves are mapped as two individual crustal domains with the Marrupa Complex overlying the Unango Complex. Both contain large volumes of orthogneiss emplaced between 1062 and 946 Ma that probably formed in a continental arc setting (Bingen et al., 2009). Granulite-facies metamorphism in the Unango Complex at ∼953 Ma is thought to be the result of the Irumide Orogeny. The Unango and Marrupa Complexes witnessed minor Neoproterozoic magmatism in the form of nepheline syenite plutons dated at ∼799 Ma. We note that similar age groups, emplacement of granitoid melt at ∼1150 Ma andhigh-temperature metamorphism at ∼750, are also found in southern Tanzania (Sommer and Kröner, 2013). In northwesternmost Mozambique, along the shore of Lake Malawi, the Unango Complex overlies the small fault-bounded Ponta Messuli Complex (Viola et al., 2008, Bingen et al., 2009). The Ponta Messuli Complex contains Paleoproterozoic metasediments that were affected by migmatitization at around 1950 Ma and intruded by 1056 Ma granite. Because of this geochronology, the Ponta Messuli Compex is considered equivalent to the Usagaran–Ubendian Belt. The Ponta Messuli Complex is also overlain by the Neoproterozoic Txitonga Group (the Cobué Group of Pinna et al., 1993), a volcanosedimentary complex with minor rhyolite flows, attesting to bimodal magmatism coeval with the development of aCryognian basin.

The Nampula Complex to the south of the Lurio Shear Belt mainly consists of late Mesoproterozoic crust (1125–1075 Ma). It has been subdivided by lithology and age into the Mocuba Suite, Rapale Gneiss, Molocue Group, Namala Gneiss and Culicui Suite (Macey et al., 2010). The oldest of these units, the Mocuba Suite, contains TTG suites and granitoids intruded between 1148 and 1114 Ma and is interpreted to have evolved in a Mesoproterozoic juvenile island arc system. Emplacement of the Rapale Gneiss at 1095–1091 Ma probably occurred during subduction and accretion. The younger Molocue Group contains metasediments with detrital zircon ages clustering around 1000 and 1800 Ma and probably evolved in a back arc basin setting. A final phase of Mesoproterozoic activity is represented by the intrusion of voluminous granitic plutons and sheet-like bodies of the Culicui Suite (1087–1040 Ma), which have A-type granite geochemical characteristics. They are interpreted to have been generated in a late tectonic, extensional setting, culminating at ∼1075 Ma. Although the units north and south of the Lurio Belt share a similar Mesoproterozoic crustal growth history, their evolution was diachronous. The Nampula Complex south of the Lurio developed between 1125 and 1075 Ma, the Unango and Marrupa Complexes north of the Lurio Belt between 1062 and 946 Ma.

### 3.5 The Mozambique Belt in Madagascar – reworked crustal fragments

The rocks of the Antogil and Masora Cratons exposed at the eastern margin of Madagascar are the oldest in the island (Fig. 4) and include orthogneiss whose tonalite precursors were emplaced at 3300–3150 Ma and intruded by 2570–2100 Ma granite (Collins, 2006, Schofield et al., 2010). They are part of the greater Indian Dharwar Craton (e.g., Tucker et al., 1999). The largest part of central Madagascar comprises the Neoarcheanarchean Antananarivo Craton that was thermally and structurally reworked between ∼750 and 500 Ma (e.g., Schofield et al., 2010, De Waele et al., 2011 and references cited therein). Two different hypotheses attempt to explain their present-day juxtaposition. The “out of Africa hypothesis” or “Azania terrane model” (e.g. Collins, 2006 and references therein), portrays them as fragments of India (Antogil and Masora Cratons) and central East Africa (Antananarivo Craton) respectively, joined along a convergent margin boundary – the Betsimisaraka suture – active throughout Neoproterozoic time (∼800–550 Ma). Another hypothesis, the “out of India hypothesis” (Tucker et al., 2011), portrays them as different parts of the Dharwar Craton joined by a Neoarchean accretion event (∼2.5 Ga). A close match of Paleoproterozoic detrital zircon ages from the Antananarivo Craton (Itremo Group) with the Ubendian–Usagaran belts of East Africa led Cox et al. (2004) and Fitzsimons and Hulscher (2005) to conclude that at least part of the Antananarivo Block was derived from East Africa.

Central Madagascar is composed of the Antananarivo Craton, the Tsaratanana Sheet, and the Itremo–Ikalamavony Domain (Fig. 4). The Antananarivo Craton consists of 2550–2490 Ma old crust tectonically interlayered with voluminous 824–719 Ma granites, syenites and gabbros (Tucker et al., 1999, Kröner et al., 2000, Collins, 2006) that have subduction-zone geochemical characteristics. The structurally upper Tsaratanana sheet, separated by a mylonite zone from the Antananarivo Craton, has similar formation ages (2750–2490 Ma) to the Antananarivo Craton, but has zircon xenocrysts that date back to 3260 Ma and show Mesoarchean isotope signatures (Tucker et al., 1999).

The Itremo–Ikalamavony Domain, subdivided into Itremo and Ikalamavony sub-domains (Tucker et al., 2011),contains Archean, Paleoproterozoic and Neoproterozoic rocks. The Itremo sub-domain consists of Paleoproterozoic metasediments that unconformably overlie amphibolites and gneisses correlated with Neoarchean orthogneisses of the Antananarivo Craton. The sedimentary protoliths were deposited after ∼1700 Ma (1800–1650; Cox et al., 1998, Cox et al., 2004, Tucker et al., 2011) and before ∼820 Ma (Collins et al., 2003a, Collins et al., 2003b). The Ikalamavony sub-domain also includes Neoarchean migmatite gneiss, but their exposures are limited in extent and their contacts with adjacent stratified gneiss are equivocal. The main unit in the sub-domain is the Ikalamavony Group, a metasedimentary assemblage intruded by 1020–982 Ma calc-alkaline magmatic rocks that appear to be absent in the Itremo sub-domain. The Ikalamavony sub-domain alsoincludes a minor unit of Ediacaran, quartz-rich metasedimentary rocks (Molo Group of Cox et al., 2004) that are absent in the Itremo Sub-domain.

(Anoysen and Androyen described here since these domains are Pre-Neoproterozoic): Pre-Neoproterozoic assemblages of southern Madagascar include the Androyen and Anoysen domains. The Androyen domain largely consists of Paleoproterozoic to Neoproterozoic sedimentary rocks intruded by Tonian anorthosites and Ediacaran granites (Tucker et al., 2011, Collins et al., 2012). The Anoysen domain in southeast Madagascar was formed between 1900 and 1680 Ma and is intruded by igneous rocks of Stenian–Tonian (1030–980 Ma) and Cryogenian (800 Ma) ages. Nd crustal residence ages of 2800–2100 Ma (Paquette etal., 1994) and abundant Neoarchean detrital and inherited zircons (Tucker et al., 2011) suggest that large parts of the Anoysen and Androyen domains are underlain by Paleoproterozoic and older crust (Tucker et al., 2011). However, whether the Anoysen and Androyen domains, together with the central Madagascan Antananarivo Craton, are part of Africa / Azania or part of India as proposed by Tucker et al. (2011) is a matter of ongoing discussion.

## 4 Accretion and thickening in the Arabian–Nubian Shield

Accretion and thickening phases are defined in different ways within the EAO. In the ANS, they are bracketed between the times of closure of individual oceanic realms formingdiverse sutures and the final build-up of the nappe stacks. In other domains, where sutures are not preserved or poorly defined, the ages of peak metamorphic conditions serve as proxies for the timing of crustal thickening.

### 4.1 Assembling the southern ANS

**Fig. 5:**
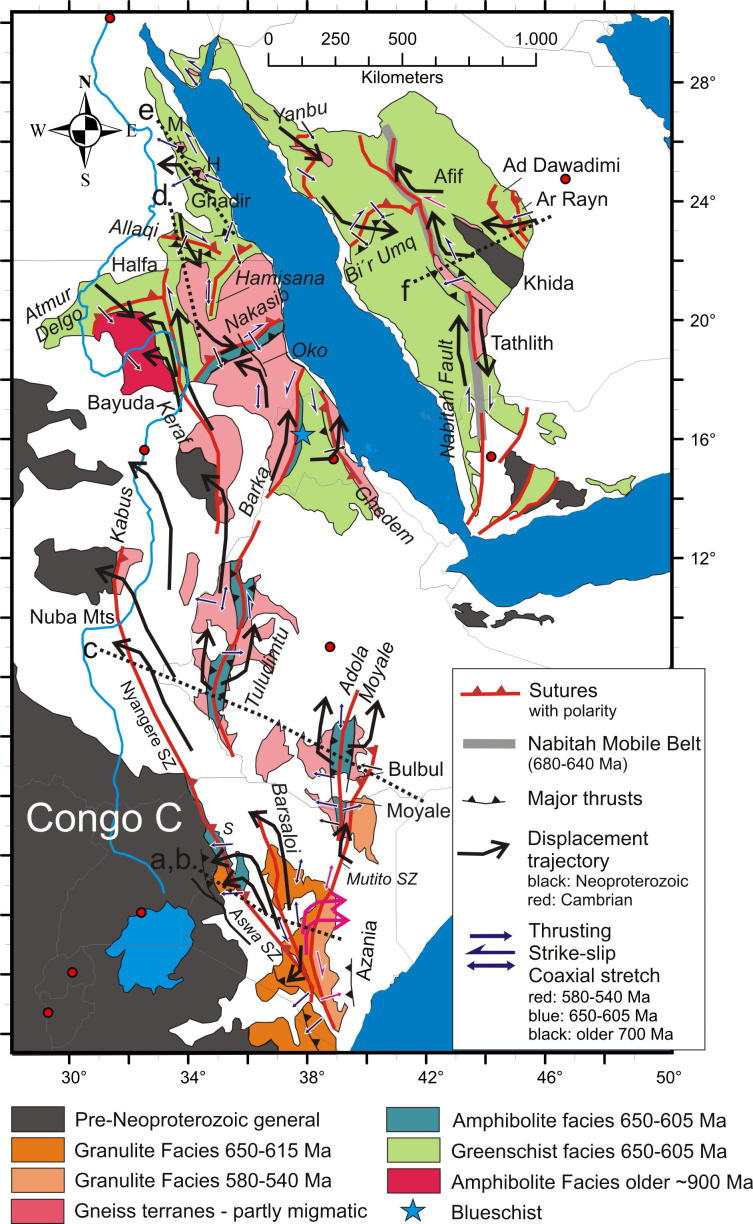
Structural and metamorphic map of the northern East African Orogen. Displacement trajectories are drawn combining successive deformation phases. Sutures and major faults are labelled in italics. M: Meatiq Dome; H; Hafafit Dome. Dotted lines a–f indicate cross sections in Fig. 6.

**Fig. 6:**
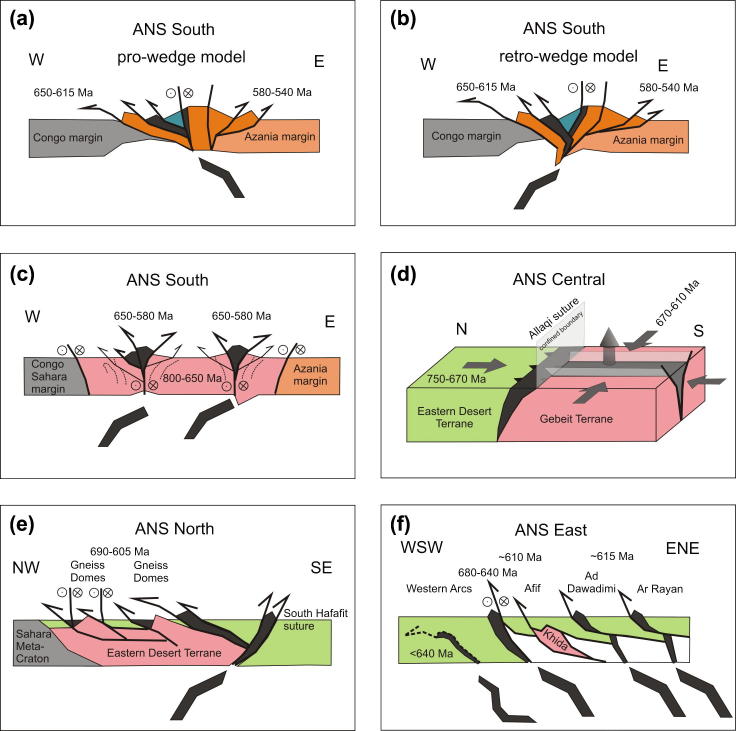
Tectonic style sketches of different ANS segments (for profile location see Fig. 5). Colour code indicates prevailing metamorphic conditions shown in Fig. 5. Dark grey are ophiolite remnants and subducted oceanic slabs. (a and b) Pro-wedge and retro-wedge models for the southern ANS margins that experienced highest grade metamorphism and thick-skinned thrusting. Note that closure of the Azania margin occurred later than closure of the Congo margin. (c) Positive flower structure geometry within the southern ANS. Deformation within roots of arc systems may have predated final emplacement of ophiolite nappes. Circle symbols denote strike slip component. (d) Opposing shortening directions between the steep southern ANS belts and the northern – central ANS terranes is compensated by amplified exhumation in the south (see symbols for motion of rocks). (e) High-grade gneiss domes are separated along shallow dipping thrusts from low-grade superficial nappes in the Eastern Desert Terrane. Low-grade nappes evolved in thin-skinned tectonic style, gneiss domes record high-temperature fabrics. (f) Polyphase shortening within the eastern ANS and diachronous closure of individual oceanic basins. For further information see text.

**Fig. 7:**
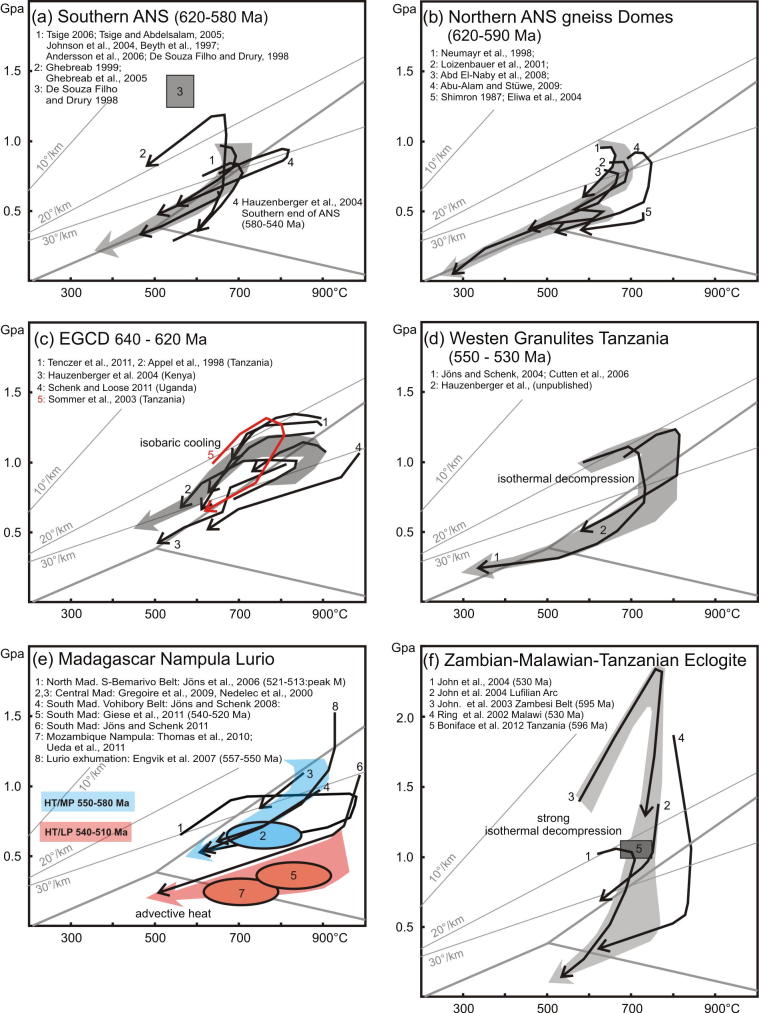
P–T diagrams paths from different EAO domains. Linear geothermal gradients and stability fields of kyanite, sillimanite, and andalusite are indicated. Grey shaded arrows are averaged trends. (a) Except of one anticlockwise P–T path, clockwise paths are reported from the southern ANS. Ghebreab (1999) and Ghebreab et al. (2005) interpret the anticlockwise path by burial of hot island arc roots followed by exhumation during orogenic collapse. (b) Clockwise P–T paths from northern ANS gneiss domes. The β-shaped loop (2) and the cooling path along the high thermal gradient (5) are interpreted as resulting from advective heat transport induced by magma emplacement together with exhumation of rocks. (c) Hot- to ultra-hot metamorphic conditions with a segment of isobaric cooling characterizes the EGCD. (d) Western Granulites clockwise P–T paths with isothermal decompression. (e) Succession of 550–580 Ma high temperature/medium pressure (blue) to 540–510 Ma high temperature/low pressure conditions (red) in southern Madagascar and the Nampula Block is explained by extension postdating the collisional event. (f) Eclogite facies metamorphism and isothermal decompression within the Irumide and Lufilian belts. For further explanation see text. (De Souza Filho and Drury, 1998, Eliwa et al., 2004, Jöns and Schenk, 2004, Shimron, 1987)

The southern end of the ANS (SE Kenya) is of prime interest in this review because here the ANS merges with the MB, in a region bounded between continental Africa in the west and Azania in the east. Here, reconstruction of the accretion and collision history is facilitated by exposures of the Galana arc–arc suture and the Voi arc-continent suture (Frisch and Pohl, 1986). Neoproterozoic island arc suites of the Galana suture (970–845 Ma) are exposed as a narrow strip between Azania and the Eastern Granulite Belt in the Taita Hills (Fig. 4). This suture separates domains of different tectonic polarities and metamorphic ages (Hauzenberger et al., 2007, Bauernhofer et al., 2008a, Bauernhofer et al., 2008b). Azania experienced E-directed stacking and folding of continental units together with granulite-facies metamorphism (peak P–T 800 °C/0.9 GPa) at 580–540 Ma. By contrast, the Eastern Granulites underwent SW-directed nappe stacking at slightly higher metamorphic conditions (peak P-T 850 °C/1.1 GPa) between 650 and 620 Ma (Hauzenberger et al., 2004) (Fig. 5, Fig. 6, Fig. 7). The arc–arc suture itself is a steep, NNW-elongatedbelt aligned approximately parallel to the prominent Aswa–Nyangere and Mutito shear zones that converge at the southern tip of the ANS. Kinematic studies reveal that the early phases of deformation were characterized by low vorticity sinstral shear (accompanied by a significant pure shear component) and subsequent localized dextral shearing (Bauernhofer et al., 2008b) (Fig. 5). The Voi suture, exposed within the Taita Hills, contains mafic and ultramafic rocks and separates a shelf sequence (the Kurase Group, part of the Eastern Granulites) from an overlying downwarped basin assemblage that formed in the vicinity of the continental margin (the Kasigau Group). The suturing event is bracketed between 850 Ma (emplacement of magmatic rocks within the Kasigau Group) and 650–620 Ma, the time when both units shared a common granulite faciesmetamorphism. This coincided with southwestward thrusting of the Kasigau Group over the Kurase Group. Azania became fully attached between 580 and 550 Ma, at a time of dextral shearing within the Galana suture and eastward thrusting within the Azania shelf sequence.

Similar structures and metamorphic conditions occur along the southwestern ANS margin in western Kenya and Uganda. The ophiolitic suite of western Kenya and its continuation into Uganda (Vearncombe, 1983, Berhe, 1990) is an upper amphibolites-facies metamorphic assemblage thrust westward onto the Congo Craton and imbricated with shallow water continental shelf sediments and slivers of the reworked craton. Arc-related intrusive igneous rocks were dated at ∼663 Ma, and collision was completed before 593 Ma, prior to intrusion of late tectonic plutons (Ries et al., 1992). The east margin of the Congo Craton was intensely reworked at this time and contains 740–630 Ma charnockite and granitoid intrusions. Granulite-facies metamorphism (870–970 °C) at 630–615 Ma is attributed to the collisional event (Mänttäri et al., 2011, Mäkitie et al., 2011, Schenk and Loose, 2011).

Farther north, the ANS west margin is delineated by the N-trending Keraf–Kabus suture from which the E-trending Atmur-Delgo suture extends west and southwestward (Fig. 5). The ∼750–650 Ma old, SE-verging Atmur-Delgo fold-and-thrust belt is interpreted as having formed above a N-dipping subduction zone during the closure of a restricted oceanic basin that existed between the southern Bayuda and northern Halfa terranes. TheKeraf suture developed as a result of closure of a back-arc basin and remnants of a passive margin where marbles and carbonate-rich turbidites were deposited. The suture displays structural styles compatible with a sinistral transcurrent tectonic regime resulting from a NW–SE collision that occurred between 640 and 580 Ma (Abdelsalam and Stern, 1996, Abdelsalam et al., 1998, Abdelsalam et al., 2003, Bailo et al., 2003).

A prolonged Neoproterozoic tectonic history is recorded within the high-grade metamorphosed suites to the west of the Keraf suture. These high-grade rocks are subdivided into an external (western) Rahaba-Absol Terrane and an internal (eastern) Kurmut Terrane (Küster and Liégeois, 2001, Küster et al., 2008). The Tonian Rahaba-Absol Terrane (also known as Bayuda Terrane), which is part of the Sahara Metacraton, experienced early Neoproterozoic (921 Ma) amphibolite-facies metamorphism and deformation, followed by granite–granodiorite magmatism at ∼900 Ma. Küster et al. (2008) referred to this metamorphic–magmatic event as the Bayudian event.

Unlike the northern, NE-trending Nakasib-Bír Umq suture, the ANS arc–arc sutures to the south (Barsaloi–Tuludimtu–Baraka sutures and Galana–Adola–Moyale–Ghedem-Arag-sutures) trend N–S (Fig. 5). They consist of meta-volcanosedimentary rocks such as garnet–staurolite and amphibole schists, metamorphosed up to amphibolite facies (Ghebreab, 1996, 1999; Yihunie and Hailu, 2007). Most are decorated by linear belts of mafic and ultramafic rocks with ophiolitic affinity. The sutures are flanked by partly migmatic gneiss terranes (given different names in different places) where rocks show higher grades of metamorphism (Tsige and Abdelsalam, 2005, Yihunie et al., 2006, Yihunie and Hailu, 2007, Stern et al., 2010b, Woldemichael et al., 2010). The nature of these orthogneiss terranes is debated. They might represent either pre-Neoproterozoic crust or roots of Neoproterozoic arcs. Recent zircon geochronology in southern Ethiopia (Stern et al., 2010b) establishe that the bulk of these gneisses are Neoproterozoic. However, one sample from undeformed granite intruding a migmatitic gneiss has abundant ∼2500 Ma old zircons. Stern et al. (2010b) took this as argument that Archean crust exists locally at depth insouthern Ethiopia. Two pulses of magmatism (at 860–850 and 795–785 Ma) are discerned in the Tuludimtu region (Woldemichael et al., 2010), and four magmatic episodes (890–840, 790–700, ∼660 and 630–500 Ma) in the Adola Moyale Belt (Stern et al., 2010b). The oldest age dates the beginning of crust formation, interpreted as the result of rifting of Rodinia and the formation of an early passive margin. The period between ∼850 and 700 Ma is considered to be associated with arc to back-arc formation. The period between 660 and 500 Ma has been related to convergent tectonics and exhumation.

In Kenya and Ethiopia, W- and E-directed thrusting of individual units above the high-grade gneissic terranes are interpreted as an early phase of deformation between 800 and 650 Ma (Yibas et al., 2002, Yihunie and Tesfaye, 2002, Allen and Tadesse, 2003, Yihunie and Hailu, 2007). This event was related to collision of individual terranes after consumption an ocean along a possibly E-dipping subduction zone (Bulbul and northern Moyale belts: Tsige and Abdelsalam, 2005). N-trending strike-slip shearing was initiated soon after or contemporaneous with thrusting. The Adola-Moyale belt has been described as a positive flower structure (Tolessa et al., 1991, Ghebreab, 1992) and all subsequent authors typify the southern ANS belts as a transcurrent orogen with an oblique NW–SE to NE–SW shortening component (Fig. 5, Fig. 6). Regional E–W shortening resulted in both lateral and pronounced vertical flow of rocks as suggested bythe involvement of metamorphic terranes exhumed from 25 to 35 km (Johnson et al., 2004, Tsige, 2006) or even 45 km depth (Ghebreab et al., 2005). The superposition of strike-slip shearing with thrust-related fabrics produced the complex fold interference pattern typical for these belts (Bogliotti, 1989, Bonavia and Chorowicz, 1993). It is suggested that both sinistral and dextral displacement was active over considerable time (Woldehaimanot and Behrmann, 1995, Worku and Schandelmeier, 1996, Braathen et al., 2001, Yibas et al., 2002, Yihunie and Tesfaye, 2002, Tsige and Abdelsalam, 2005, Yihunie and Hailu, 2007).

Eritrea has a 850–650 Malow-grade metamorphic central volcanosedimentary domain comprising the Haggar, Nakfa, Adobha Abi terranes, flanked by amphibolite-facies gneiss domains (Drury and De Souza Filho, 1998, Ghebreab, 1999, Andersson et al., 2006). The western gneiss terrane is known as Barka terrane and the eastern as Ghedem-Arag terrane (Fig. 5). The intermediate terranes have been interpreted as an intra-arc system or paleo-oceanic troughs located above a W- to NW-dipping subduction zone (Ghebreab et al., 2009). Rocks within E-verging imbricated thrust sheets were affected by subduction-related metamorphism of ∼550 °C and 1.45 GPa (Fig. 7; Drury and De Souza Filho, 1998). The high-gradeterranes that bound the central volcanosedimentaryterranes show metamorphic conditions up to 700 °C and 1.2 GPa (Beyth et al., 1997, Ghebreab, 1999). The timing of deformation and metamorphism related to crustal thickening is best constrained by emplacement of pre- to syn deformational gneisses between 850 and 770 Ma (Ghebreab et al., 2005). Orogen-parallel strike-slip shearing, coeval with or subsequent to subduction, occurred between 650 and 580 Ma (Ghebreab, 1999, Ghebreab et al., 2005). Ghebreab et al., 2005, Ghebreab et al., 2009 indicate that the orogenic belts in Eritrea evolved through transpressional tectonic regimes. Wrench tectonics in the region concentrated along two shear belts.The western Barka sinistral shear zone is probably a northward continuation of the Tuludimtu Belt in Ethiopia (Fig. 5), and the eastern Ghedem–Araq shear belt (Asmara–Nakfa Shear Belt) a continuation of the Adola–Moyale Belt. NNE-trending sinistral strike-slip shear zones dominate much of the Barka shear zone, local dextral strike-slip is interpreted as younger structural overprints (Ghebreab et al., 2009).

### 4.2 Assembling the central ANS

Two major sutures are recognized in the central ANS that, in contrast to the southern ANS, trend E–W and SW–NE. The Nakasib-Bír Umq suture extends from central Sudan to western Arabia and the Allaqi-Heiani-Sol Hamed-Yanbu suture, also referred to as Yanbu-Onib-Sol Hamed-Gerf-Allaqi-Heiani (YOSHGAH) suture (Abdelsalam and Stern, 1996, Ali et al., 2010a) extends from southern Egypt and northern Sudan to northwestern Arabia. In Sudan and Egypt, both sutures are cut by younger N–S shear zones, the Oko Belt in the south and the Hamisana Belt in the north. In Arabia, the SW–NE trending sutures are truncated by the Nabitah Belt that aligns partly with the Najd Fault System (Fig. 5).

The Nakasib-Bír Umq arc–arc suture formed as a result of collision between the southern Jiddah-Haya terranes and the northern Hijaz-Gebeit Terranes (Abdelsalam and Stern, 1993, Stern and Abdelsalam, 1998, Johnson et al., 2003) (Fig. 2). Emplacement of greenschist-facies (locally metamorphosed to amphibolites-facies), SE-verging nappes during earlystages of the Nakasib deformation, resulted from closing of an oceanic domain along a N-dipping subduction zone at ∼750 Ma (Abdelsalam and Stern, 1993, Abdelsalam, 2010), a model that is contradicted by Schandelmeier et al. (1994) and Wipfler (1996). Some oblique slip is inferred from flower structure geometry associated with dextral displacement along the southern margin of the Nakasib suture (Schandelmeier et al., 1994, Johnson et al., 2003). Similarly, the Bír Umq suture has been interpreted as a flower structure including steeply dipping dextral shears and S-vergent thrusts (Johnson et al., 2003).

The Allaqi-Heiani-Sol Hamed-Yanbu arc–arc suture can betraced for ∼600 km across the Nubian and Arabian shields (Pallister et al., 1988, Abdeen and Abdelghaffar, 2011). The Allaqi-Heiani-Onib-Sol Hamed segment of this suture separates the Eastern Desert terrane in the north from the Gebeit terrane in the south. The Yanbu suture of Saudi Arabia delineates the northern Midyan from the southern Hijaz Terrane (Fig. 2). The ophiolite within the Allaqi segment appears to have been emplaced SSW-ward above a NNE dipping subduction zone (Kusky and Ramadan, 2002, Abdelsalam et al., 2003, Abdeen and Abdelghaffar, 2011) soon after its formation, i.e., 730–697 Ma (Ali et al., 2010a). Arc metavolcanic rocks are metamorphosed to greenschist-facies conditions but contain a metamorphicsole at the base of the ophiolite metamorphosed to 700 °C and 0.65–0.85 GPa (El-Nisr, 1997). Blueschist-facies metamorphic assemblages (awaiting verification) have been reported from slices within western portions of the belt (Taylor et al., 1993). Subsequent to thrusting (750–650 Ma: Abdeen and Abdelghaffar, 2011), oblique convergence released slip parallel to the belt (both sinistral and dextral). Ali et al. (2010a) concluded that the YOSHGAH ophiolite belt formed in two stages (∼810–780 Ma and ∼730–750 Ma) and indicate that arc–arc collision between the Gabgaba–Gebeit–Hijaz terranes to the south and the Eastern Desert–Midyan terranes to the north occurred as early as ∼730 Ma and no later than ∼709 Ma.

### 4.3 Assembling the northern ANS

In contrast to the steep, linear belts exposed in the south, rocks with oceanic affinity in the Eastern Desert terrane (Fig. 2) are present along shallow dipping thrusts. Nevertheless, identification of distinct subduction zones and subduction polarity remains speculative. A switch from early E-dipping subduction (∼830–700 Ma) to late W-dipping subduction (∼630 Ma) is proposed (Abd El-Rahman et al., 2009) for the Fawahir ophiolite, adjacent to the Meatiq dome (Fig. 5), whereas the Hafafit–Ghadir domain is interpreted as a back-arc accretion system that evolved above anorthwest-dipping subduction zone (Abd El-Naby and Frisch, 2006, Khalil and Azer, 2007, Abd El-Naby et al., 2008). In contrast, Azer and Stern (2007) summarized the compositions of spinels and whole rock major elements for the Eastern Desert serpentinites and concluded that these originated by forearc seafloor spreading. A generalized view on the tectonic evolution of the Eastern Desert Terrane by El-Sayed et al. (2007) includes SE-dipping subduction between 850 and 780 Ma, followed by arc splitting and bipolar subduction with SW- and NE-polarities between 780 and 620 Ma.

The crustal structure of the Eastern Desert terrane comprises structurally lower and higher-grade gneissic domes (structural basement) overthrust by structural cover nappes of lower-grade volcanosedimentary sequences (Fritz et al., 1996). Except for SSE-directed thrusting associated with accretion and obduction of an intra-oceanic arc in the southernmost part of the Eastern Desert terrane (Abdeen et al., 2008), there is overwhelming agreement that the terrane elsewhere was affected by NW-directed thrusting, which continued until ∼610 Ma (Fig. 6; Greiling et al., 1994, Fritz et al., 1996, Bregar et al., 2002, Fowler and El Kalioubi, 2002, Fowler and El Kalioubi, 2004, Fowler et al., 2007, Andresen et al., 2009, Andresen et al., 2010, Shalaby, 2010, Augland et al., 2012). The age of thrusting, as inferred from crosscutting relationships of granitoids and cooling ages is bracketed between 690 and 650 Ma (Sibai dome: Bregar et al., 2002, Fowler et al., 2007, Augland et al., 2012) and 630 and 609 Ma (Meatiq and Hafafit domes: Andresen et al., 2009).

Volcanosedimentary squences of the cover nappes evolved in thin-skinned tectonic style and experienced maximum metamorphic conditions of ∼500 °C and 0.5 GPa. The structural basement experienced high-grade metamorphism and deformation with peak P–T conditions around 680 °C and 0.8 GPa (Neumayr et al., 1998, Loizenbauer et al., 2001, Abd El-Naby et al., 2008, Abu-Alam and Stüwe, 2009) (Fig. 7). This suggests detachment between deeply buried island arc roots (now exposed within the gneissic domes) and volcanosedimentary cover nappes that remained at shallow crustal levels. Pressure–temperature evolutionary paths from basement and cover nappes overlap between ∼620 and ∼590 Ma when the structural basement was exhumed and structural cover units were advectivly heated by the exhumation process itself and/or by coeval emplacement of large volumes of granitoids. Gneissic domains in Sinai are intruded by large volumes of late tectonic plutons that frequently preclude recognition of structures along the contact between high-grade gneiss terranes and the volcanosedimentary carapace. Early thrust sheets may have been transported SSE-ward and are later transposed by sinistral shear (El-Shafei and Kusky, 2003, Abu-Alam and Stüwe, 2009, Fowler et al., 2010a, Fowler et al., 2010b).

### 4.4 Assembling the eastern ANS

Terranes of southern and western Saudi Arabia (Midyan, Hijaz, Jiddah, Asir terranes) are separated from the Afif and Tathlith terranes farther east by the N- to NW-trending Nabitah suture (Nabitah mobile belt: Stoeser and Stacey, 1988) (Fig. 2, Fig. 5). The Nabitah suture is aligned in large portions with the post-accretionary Najd fault system, making identification of the early accretion-related structures difficult. Gneiss belts that extend about 1300 km across the entire Arabian Shield are typically associated with the Najd fault system. The gneiss domains experienced amphibolites-grade metamorphism up to migmatisation and are surrounded by rocks with upper greenschist facies mineral assemblages. Sinistral transpression in the course of bulk WNE–WSE convergence between the Afif and Hijaz–Jiddah–Asir terranescharacterizes this steep belt (Quick, 1991, Genna et al., 2002, Johnson and Kattan, 2001). Both, SW- and NE-verging thrusts with a minor amount of lateral displacement are best interpreted as positive flower structures (Fig. 5, Fig. 6). The Nabitah fault (*sensu stricto*) constitutes a dextral, N–S trending strike slip system that makes up the southern part of the Nabitah belt. This fault originated during the Nabitah orogeny (680–640 Ma: Stoeser and Stacey, 1988) but underwent renewed slip after intrusion of late-tectonic 640 Ma granitoids (Stern and Johnson, 2010).

The youngest terranes in the ANS, with upper Cryogenian to Ediacaran protolith ages, are the AdDawadimi and Ar Rayn terranes in the easternmost Arabian Shield (Fig. 2, Fig. 5). The Ad Dawadimi and Ar Rayn terranes amalgamated through southwestward thrusting at ∼620 Ma and were jointly thrust westward onto the Afif terrane by ∼607 Ma (Fig. 7; Cox et al., 2012, Johnson et al., 2011).

### 4.5 Assembling juvenile Madagascar

**Fig. 8:**
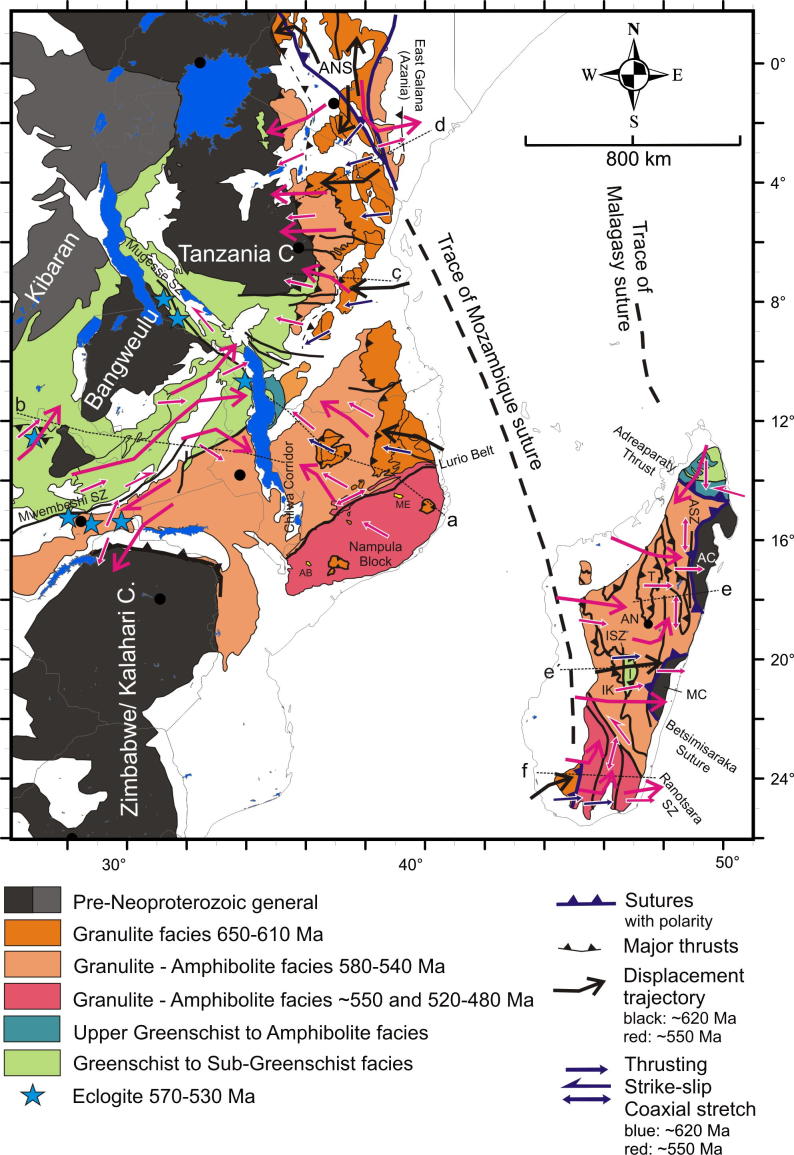
Structural and metamorphic map of the southern EAO. Displacement trajectories are drawn combining successive deformation phases. Dotted lines a,b,c,d,e,e′,f locate cross sections of Fig. 9. AB: Alto Benfica Group; AC: Antogil Craton; AN: Antananarivo Block; ANS: Arabian–Nubian Shield; ASZ: Angavo Shear Zone; I: Itremo Group; IK: Ikalamavony Domain; ISZ: Itremo Shear Zone; MB: Mecubúri Group; MC: Masora Craton; T: Tsaratanana Sheet.

The Vohibory Belt of southern Madagascar with its Neoproterozoic oceanic crust (Jöns and Schenk, 2008, Tucker et al., 2011) may be regarded as the southward continuation of the ANS (Fig. 4). It is interpreted as anE-verging continental accretionary wedge, emplaced onto the Azania margin across a W-facing continental ramp (De Wit, 2003, Emmel et al., 2008). Granulite-facies metamorphism dated at 612 Ma in the Voribory Belt occurred during accretion of the arc terrane and closure of a back-arc basin (Fig. 7, Fig. 8).

The Bemarivo Belt of northern Madagascar (Fig. 4, Fig. 8) probably delineates the eastern border of Azania. It is composed of two Neoproterozoic arc-marginal basin terranes separated by a major ductile shear zone (Thomas et al., 2009). The southern terrane is a sequence of high-grade paragneisses (upper amphibolites- to granulite-facies Sahantaha Group); the northernterrane is variably metamorphosed volcanosedimentary sequences (amphibolites-facies Milanoa Group and greenschist-facies Daraina Group). Both terranes were translated southward over “cratonic” Madagascar along the Andraparaty Thrust (De Waele et al., 2011) at ∼540 Ma. Monazite dating from the southern Bemarivo Belt (Jöns et al., 2006), indicates prograde metamorphism at 563–532 Ma and granulite-facies peak metamorphism at 521–513 Ma. The 563–532 Ma ages are inferred to reflect the attachment of the Bemarivo Belt to the amalgamating Gondwana supercontinent during the course of which, the southern Bemarivo Belt was buried to a depth of >25 km.

### 4.6 Synopsis of accretion and thrusting within the ANS

The distribution of crustal age domains indicates heterogeneous growth of the ANS and diachronous subduction/accretion processes (Fig. 3). Ages interpreted to date nappe stacking and metamorphism cluster between 670 and 600 Ma (650–615 Ma: southern and western ANS; 650–600 Ma: central ANS; 650–600 Ma: northern ANS; 620–607 Ma: eastern ANS; 612 Ma: Vohibory Belt). A closer look at the data indicates that prolonged shortening was initiated as early as 750 Ma in the southern ANS whereas oceanic domains where still open in the eastern ANS. Closure of the Mozambique Ocean and final docking of ANS fragments with the African margin was approximately contemporaneous at ∼620–610 Ma. High-grade 580–550 Ma metamorphism in the Bemarivo Beltindicates that the ocean along the eastern shore of Azania (Malagasy Ocean: Collins and Pisarevsky, 2005) closed later.

Tectonic styles and the degree of metamorphism differ throughout the ANS (Fig. 5, Fig. 6). The southernmost ANS is dominatly a pure-shear transcurrent orogen associated with a significant amount of vertical exhumation locally exposing granulite-facies rocks. Along the western ANS margin, oblique convergence was partitioned into sinistral shear and westward-directed thrusting. Granulite-facies metamorphic conditions developed in the southern ANS (Kenya, Uganda) associated with thick-skinned thrusting, but the degree of metamorphism decreased northward (Sudan). Transcurrent motion with significant coaxial N–S stretching dominated the southern steep belts such as Tuludimtu and Adola Moyale belt (Fig. 5, Fig. 6). Ophiolite-bearing sutures are metamorphosed up to amphibolites-facies conditions and flanked by high-grade, partly migmatic gneisses. The high-grade gneisses most probably represent roots of island arcs that experienced an early phase of deformation (∼800–650 Ma), probably related to shearing coeval with, or soon after their emplacement. Both, W- and E-directed thrusting evolved from positive flower structures between 650 and 580 Ma. Rare data indicate subduction-related metamorphism under conditions up to 550 °C and 1.45 GPa (Eritrea: Drury and De Souza Filho, 1998) whereas adjacent gneisses underwent high-temperature metamorphism at a higher thermal gradient (∼750 °C at 0.9 GPa). We argue for a prolonged deformation history in which the earliest fabrics evolved through formation ofisland arcs associated with high-temperature–moderate-pressure metamorphism reflecting a high thermal gradient, followed by accretion and the rare preservation of subduction-related metamorphism at a low thermal gradient.

The NE–SW trending Nakasib-Bír Umq arc–arc suture and the Allaqi-Heiani-Sol Hamed-Yanbu arc–arc suture in the central ANS developed by closure of oceanic basins ∼750 Ma and accretion ∼650 Ma, approximately coeval with the N–S trending steep belts farther south. Both suture zones record N–S shortening and southward thrusting above N-dipping subduction zones (Fig. 6). Simultaneous N–S shortening along these sutures and N–S horizontal stretching in the southern steep belts implies space problems and interference between structures at the contact between the southern and the central ANS domains (Fig. 5, Fig. 6). The E–W trending intra-arc sutures are interpreted as the confined northern boundary of the southern, N–S stretched terranes. Opposing shortening directions were compensated by amplified vertical rocks flow within the southern units, which might explain the generally higher-grade metamorphic conditions recorded within the southern ANS.

By contrast to the steep and narrow southern sutures, the Eastern Desert terrane exhibits northwestward thrusting along shallow-dipping thrusts, and has a wide distribution of ophiolite remnants. Although dispersal of units by extension tectonics may have played an important role, the existence of horizontal thrust planes suggests large transport distances during thrusting. In the sketch shown in Fig. 6 we show a model proposed by Abd El-Naby and Frisch, 2006, Abd El-Naby et al., 2008 andAbdeen and Abdelghaffar (2011) interpreting the southern Eastern Desert as closure of back-arc systems behind a northwestward-dipping subduction zone represented by the “South Hafafit suture”. According to this model, the northwestward thrusts evolved in a retro-wedge setting between ∼650 and 600 Ma. The thrusts separate high-grade gneissic cores from low-grade supracrustal nappes that display a thin-skinned tectonic style. At the same time, we recognize the strong arguments in favor of a forearc setting for these ophiolites (Azer and Stern, 2007).

The eastern ANS exposed in the Arabia Shield consolidated through westward thrusting of the Ad Dawadimi and Ar Rayan terranes by ∼615 Ma, followed by attachment to the Afif composite terrane by ∼607 Ma (Johnson etal., 2011). Westward stacking of supracrustal nappes in thin-skinned tectonic style was increasingly transferred to left-lateral motion along the northern segment of the NW-trending Nabitah orogenic belt, the precursor of the Najd Fault system (Fig. 5). Dextral slip along the N–S trending southern segment of the Nabitah belt (Nabitah Fault) may be envisaged as a conjugate system during bulk E–W convergence. The presence of two very large shear systems with opposite slip suggests that the previously consolidated western arc terranes (Midyan, Hijaz, Jiddah, Asir terranes) acted as an indenter during accretion of younger terranes.

## 5 Thickening in the collisional belts of SE-Africa and Madagascar

Two distinct tectonothermal cycles are recognized in southeastern Africa andMadagascar. Late Neoproterozoic deformation between ∼650 and 620 Ma (East African Orogeny) characterizes the largely juvenile Eastern Granulite–Cabo Delgado Nappe Complex (EGCD). Late Neoproterozoic to early Paleozoic nappe assembly (Kuunga Orogeny: ∼600–500 Ma) affected other continental fragments that had also experienced pre-Neoproterozoic orogeny of various ages.

### 5.1 The East African Orogeny within the Mozambique Belt

Evidence of ∼655–620 Ma granulite-facies metamorphism is found throughout the EGCD from Uganda, through southern Kenya and Tanzania, to northern Mozambique (Fig. 8). The Lurio Belt dividing Mozambique into a northern unit and a southern domain (Nampula Block) contains lithologies and relict ages comparable to the EGCD but was intensivelyreworked during the Kuunga Orogeny around 550 Ma (e.g., Engvik et al., 2007, Ueda et al., 2012). Granulite-facies (0.9–1.2 GPa, 755–800 °C) metamorphic ages of 630–600 Ma have been obtained from the Vohibory domain in southwestern Madagascar (e.g., Jöns and Schenk, 2008).

Metamagmatic rocks at the structural base of the EGCD yield emplacement ages of 740–684 Ma in Uganda (Mäkitie et al., 2011, Schenk and Loose, 2011), 880–820 Ma in Tanzania (Tenczer et al., 2006 and references cited therein), 973–740 Ma in Mozambique (Bingen et al., 2011) and850–700 Ma in southwestern Madagascar (Jöns and Schenk, 2008). The igneous rocks were emplaced in extending lower crust or represent roots of volcanic arcs. Metasediments at the structural top of the EGCD, including 800–600 Ma marble suites (Melezhik et al., 2008), were deposited on thinned crust. The EGCD suite of Mozambique is considered to be remnants of a Neoproterozoic accretionary collage thrust west to northwest toward the Congo–Tanzania margin (Viola et al., 2008, Bingen et al., 2011). Equivalent late Neoproterozoic rocks in the Vohibory domain of southern Madagascar are inferred to be part of an east-verging continental accretionary wedge, emplaced across a west-facing continental ramp onto the Azania margin (De Wit, 2003, Emmel et al., 2008).

Penetrative deformation coeval with granulite-facies metamorphism around 630 Ma characterizes the internal portions of the EGCD, although some data point to an earlier metamorphic pulse between 760 and 730 Ma (Viola et al., 2008, Bingen et al., 2011). However, final (north)westward transport of the EGCD onto the Western Granulites of Tanzania and the Mesoproterozoic Unango and Marrupa Complexes of Mozambique occurred later, between 596 and 550 Ma (Fig. 8; Rossetti et al., 2008; Viola et al., 2008, Bingen et al., 2011). The time gap of at least 30 Ma between EGCD internal deformation and final emplacement of the EGCD nappeassembly raises questions about the ancestry and provenance of those units, and it has been proposed, for example, that the nappes are far-travelled and originated “outboard”, i.e., southeast, of present-day Mozambique (Viola et al., 2008).

**Fig. 9:**
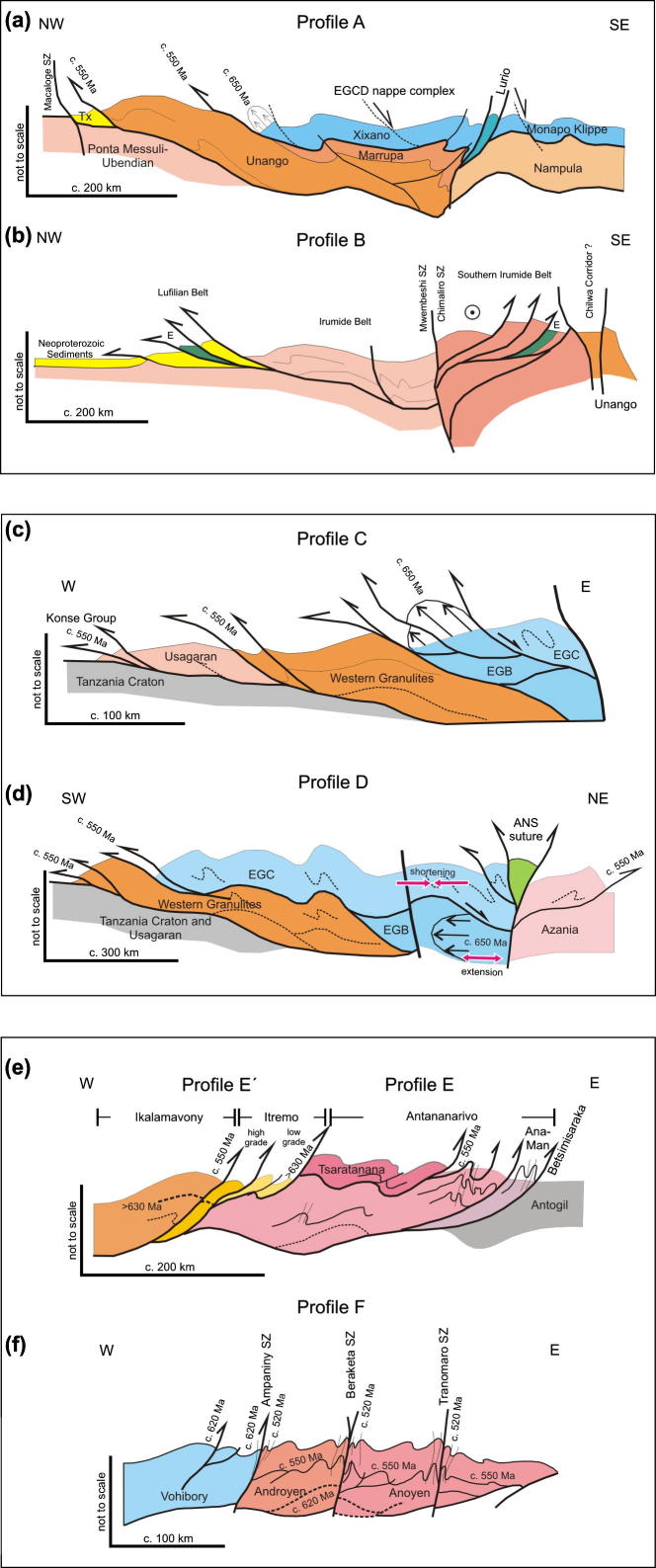
Tectonic style sketches from southern EAO segments (for profile location see Fig. 8). (a) Cross section through Mozambique showing two-phase deformation. The ∼550 Ma Kuungan deformation refolds the ∼650 Ma distributed flow (array of arrows) in internal portions of the EGCD. Kuungan thrusts climb westward and overthrust the low-grade metamorphosed Txitonga Group (Tx) and the Ubendian foreland. (b) Fan-like thrusts within the transcurrent Irumide Belt with incorporated eclogite lenses (E). (c) Kuungan and East African thrusting in southern (c) and northern (d) Tanzania. Channelled flow within the Eastern Granulite metamagmatic basement (EGB) is indicated by array of arrows. Horizontal shortening within Eastern Granulites metasedimentary cover (EGC) and coeval horizontal extension within the EGB (red arrows) requires a decollement between both units. (e) Out-of-sequence thrusting within central Madagascar. Itremo and Ikalamovony units were first thrust (∼630 Ma) onto Antananarivo. Ongoing shortening (∼550 Ma) resulted in final emplacement of Itremo units and amplified folding in the Anaboriana–Manampotsy belt (Ana–Man). (f) Deformation stages within southern Madagascar. The Voribory domain experienced ∼620 Ma thrusting. Androyen and Anoyen domains are additionally overprinted by ∼550 Ma deformation events. Ongoing W–E shortening culminated in amplified folding and development of steep N–S trending shear zones. For further information see text.

Tectonic styles (Fig. 9) and pressure temperature evolutionary paths (Fig. 7) for the basal metamagmatic suite of the EGCD display subhorizontal high-temperature flow during partly extreme temperature conditions (exceeding 900 °C at peak pressures between 1.2 and 1.4 GPa). Top-to-the northwest tectonic transport is recorded from Mozambique (Viola et al., 2008); melt-assisted top-to-the west displacement with a prominent coaxial component offlow prevailed in Tanzania (Fritz et al., 2005, Fritz et al., 2009). The tectonically upper metasedimentary part of the EGCD suite, in contrast, experienced E–W to NW–SE horizontal shortening, best evidenced by upright, N–S trending folds found in marble beds. Complex fold interference patterns with dome-and-basin geometry occur within Tanzania (Fritz et al., 2009, Le Goff et al., 2010), Mozambique (Viola et al., 2008) and in the Vohibory domain of southwestern Madagascar (Martelat et al., 2000, De Wit et al., 2001).

Pressure–temperature paths within the EGCD basement (Fig. 7) differ from those in all other domains within the EAO, and are characterized by well-defined isobariccooling from ∼900 °C down to 700 °C at pressures of ∼1.3 GPa (Appel et al., 1998, Hauzenberger et al., 2004, Jöns and Schenk, 2008, Schenk and Loose, 2011, Tenczer et al., 2011). Whether prograde metamorphism had a clockwise or anticlockwise P–T path is debated (Appel et al., 1998, Sommer et al., 2003, Fritz et al., 2009), although most data suggest an anticlockwise path. Evidently, the EGCD nappe complex experienced prolonged, slow cooling at deep crustal levels (Appel et al., 1998, Möller et al., 2000, Hauzenberger et al., 2005, Emmel et al., 2008).

Dynamic models explaining internal deformation in the EGCD need to incorporate factors of: (1) a high thermal gradient; (2) slow cooling(1–3 °C/Ma) from ∼850 °C down to ∼700 °C at pressures around 1.2 GPa, which implies a prolonged residence time of rocks at deep crustal levels consistent with the evidence of isobaric cooling (IBC) (Fig. 7); (3) a rock fabric that implies penetrative deformation concomitant with IBC rather than static annealing during prolonged residence at low crustal levels without deformation; (4) structural heterogeneities that suggest decoupling between EGCD metamagmatic basement and metasedimentary cover.

Up to now, apparently contradictory geodynamic concepts have been proposed for the EGCD. One proposal is that a temperature increase, together with moderate pressure increase followed by a phase of IBC (i.e., anticlockwise P–T path), was related to magma underplating at the base of the crust with simultaneous intrusion (magma loading) at anactive, Andean type continental margin (Appel et al., 1998). Appel et al. (1998) indicated that tectonic thickening of a previously thinned (extended) crust would be also compatible with this P–T evolutionary path but argued that overthickening of crust was unlikely because of an absence of decompression after the metamorphic peak. This scenario of magama underplating is supported by extensive formation of anorthosite and enderbite melt that might have formed in an extending continental margin, back arc domain or within island arc roots. In our opinion, however, the absence of decompression after the metamorphic peak is poor evidence for lack of crustal thickening. We note also that field studies and model calculations from orogens with similar style (Brown, 2007, Chardon et al., 2009, Gapais et al., 2009)suggest that shortening of thin, brittle upper crust overlying a weak ductile lower crust and lithospheric mantle would yield strain patterns reflecting the overall downward motion of rocks with only limited upward motions and limited topography. Ideally, distributed compressive deformation translates into homogeneous thickening with downward particle motion active over considerable time and low exhumation rates. For this tectonic scenario the term “sagduction” was introduced (Chardon et al., 1996) to discriminate distributed downward flow of hot and weak lower crust from localized subduction of cold crust and lithospheric mantle. The associated P–T evolutionary path is close to IBC.

**Fig. 10:**
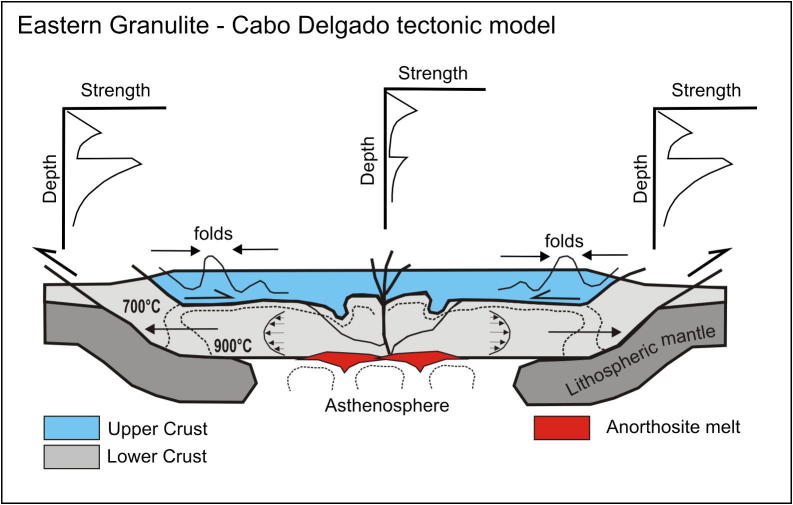
Model sketch of EGCD tectonics combining concepts of Beaumont et al., 2006, Cagnard et al., 2006, Chardon et al., 2009 and Fritz et al. (2009). Anorthosite and enderbite melts intrude extending crust and diminishe overall strength (schematic strength profiles are indicated). The low viscous lower crust flows outwards with rates faster than thermal equilibration; thereby rock particles cross disturbed thermal isogrades. Rocks cool slowly at constant depth and record isobaric cooling textures. Structural decoupling occurs along the upper surface of the flow channel (black array of arrows) arising in horizontal extensional flow within the lower crust and horizontal compressional flow and folding within the middle to upper crust. See text for further information.

A second proposal by Fritz et al. (2009) focusing on the tectonic evolution of the Eastern Granulite belt of Tanzania, is a model of lower crustal flow adopted from thechannel flow model of Beaumont et al., 2004, Beaumont et al., 2006. The model of Fritz et al. (2009) aimed to explain IBC within the lower crust, decoupling at mid-crustal levels, and structural heterogeneities between EGCD metamagmatic basement and metasedimentary cover units (Fig. 10). Prerequisite condition for lower crustal homogenous flow is a hot, low viscous, melt-penetrated lower crust. Channeled flow is triggered by gravitational energy, implying thickened crust and large, but not necessarily high, orogenic plateaus. Isobaric cooling is explained by horizontal flow with rates faster than thermal equilibration of the lower crust. The flow paths of rock particles consequently cross disturbed thermal isograds, and rocks cool at constant depth. Structural decoupling occurs along the upper surfaceof the flow channel, arising in horizontal extensional flow within the lower crust and horizontal compressional flow within the middle to upper crust (Fig. 9, Fig. 10).

Both concepts account for the existence of melt-weakened, high temperature lower crust with diminished strength. We believe this is best explained by crustal thinning predating contractional deformation. Similar structural and thermal characteristics seem to typify ultra-hot Proterozoic orogens (e.g., Chardon et al., 2009, Gapais et al., 2009). These orogens display long-lived convergence and consist of a thin upper crust resting on a thick “waterbed” of low viscous lower crust involving massive juvenile magmatism (Fig. 10). Homogenous shortening, together with displacement partitioning, arises in strong decouplingwith steep structures within the middle to upper crust, forming elongated basin and domes and variably sheared lower crust. The duality of the kinematic patterns between the upper and lower crust may lead to a wide spectrum of P–T histories, depending on the flow path followed by a sample during orogeny. Homogeneous thickening by combined downward movement of supracrustal rocks results in thickening with negligible topography and relief. In this setting, lower crustal rocks remain within the deep crust and cool isobarically. Hot orogens, as suggested for the Tanzania case, share many tectonometamorphic processes with ultra-hot orogens but may develop higher plateaus and thus the topographic gradient required to trigger channel flow.

### 5.2 The Kuungan Orogeny within the Mozambique Belt

Late Neoproterozoic to Cambrian convergent structures and polyphasetectono-metamorphic evolution affected large parts of southeast Africa and Madagascar during the Kuungan Orogeny between ∼596 and 500 Ma. Reported ages for Kuungan collisional metamorphism and deformation are between 596 and 550 Ma in the Irumide and Lufilian Belts of Zambia and Malawi (Kröner et al., 2001, Johnson and Oliver, 2004, Johnson et al., 2005) and the Ubendian Belt of southern Tanzania (Boniface et al., 2012). Maximum crustal thickening in northeastern Mozambique (Marrupa and Unango Complexes) occurred between ∼570 and530 Ma and somewhat later (550–500 Ma) in the Nampula Block south of the Lurio Belt (Bingen et al., 2009, Macey et al., 2010, Thomas et al., 2010). Within the Usagaran Belt of Tanzania the lateNeoproterozoic to Cambrian tectonic and metamorphic overprint was generally mild and limited to discrete domains (Fritz et al., 2005, Tenczer et al., 2007). For the easterly adjacent Western Granulite Belt there is growing body of evidence for crustal thickening and thrusting around 560–530 Ma (Rossetti et al., 2008, Tenczer et al., 2012). The East Galana Terrane (coastal Kenya: Fig. 8) and central Madagascar share similar ages of high-temperature metamorphism between 550–580 (Hauzenberger et al., 2007) and ∼570–520 Ma, respectively (e.g., Berger et al., 2006, Collins, 2006, Grégoire et al., 2009, Goodenough et al., 2010, Giese et al., 2011).

#### 5.2.1 Styles of nappe assembly within southern segments (Mozambique, Malawi, Zambia)

The oldest recorded Kuungan high-grade metamorphism in the Mesoproterozoic basement of the Unango and Marrupa Complexes (amphibolite to granulite facies conditions) is ∼555 Ma. This event is interpreted as representing peak crustal thickening during the Kuunga Orogeny in NE Mozambique, after deposition of the marine Geci Group (∼630–585 Ma: Melezhik et al., 2006). Evidence for regional top-to-the-NW thrusting is recorded along several of the main structural contacts separating the major imbricate packages (Fig. 8, Fig. 9). Regional mapping also revealed small-scale top-to-the-SE thrusts scattered throughout theregion. These are mostly located within the individual nappes and are interpreted as the expression of local back-thrusting, related to the same period of crustal shortening (Viola et al., 2008). Progressive shortening accommodation created NE–SW trending folds that refold previously formed thrust-related fabrics (Fig. 9). Progressive NW–SE shortening evolved from discrete thrusts at the base of individual imbricated crustal slices to continuous, diffuse folding, as typical for many fold-and-thrust belts. As a result, thrusts climbed progressively upward and northwestward toward the low-grade western foreland represented by the Ponta Messuli Complex. We call attention to the fact that the progressively evolving folds are, in large part, responsible for the present-day architecture of many domains within Kuungan East Africa and determine the trends of domain and terrane boundarieswithin Tanzania, Mozambique and Madagascar.

The Nampula Block to the south of the Lurio Belt contains small, deformed basins with Early Cambrian metasediments (maximum depositional age of 530 Ma), known as the Alto Benfica and Mecubúri Groups (Fig. 8). These basins are a valuable time-marker for deformation (Thomas et al., 2010, Ueda et al., 2012) in that the principal regional pervasive ductile foliation present in all the Nampula Block rock types formed during a progressive fold-and-thrust event prior to deposition of the sediments. The foliation appears to have developed largely during coaxial flattening strain. Peak metamorphism during the Kuungan Orogeny was typically mid-upper amphibolites-facies.

The Lurio Belt holds a key position for interpreting the boundary between the Nampula Block inthe south and the Unango and Marrupa complexes in the north. It is defined by exposures of the distinctive Ocua Complex (Fig. 4), a heterogeneous unit comprising mafic to felsic granulites, segmented and tectonically interlayered with amphibolite-facies rocks (Engvik et al., 2007, Viola et al., 2008, Ueda et al., 2012). A strong planar, coaxial, high strain WNW-dipping fabric is present in the belt, typically as mylonite. Deformation intensity diminishes in the adjacent complexes away from the belt. The ∼557 Ma high-pressure granulites in the Ocua Complex with P–T estimates up to 1.57 GPa and 950 °C (Fig. 7) indicate that the formation of this belt was associated with crustal shortening leading to crustal thickening in excess of 50 km.However, the Lurio belt is not considered a “fundamental Pan-African discontinuity” (Viola et al., 2008) because of lithological continuity on both sides of the belt, but is rather the site of significant and prolonged strain accommodation. Structural coherence with the 530 Ma old Mecubúri Group and intrusion of 505 Ma felsic plutons document long-term activity. Irrespective of the original significance of the Lurio belt, there is agreement that during its late history (later 530 Ma) the Lurio Belt was site of post-collisional extension.

The Irumide and Lufilian Belts of Zambia and Malawi hold an outstanding position in understanding the Mozambique Belt because of the unique appearance of Kuungan eclogites (Vrana et al., 1975). The Irumide Belt *sensu stricto* experienced low-grade Kuungan metamorphism whereas the Southern Irumide Belt underwent intense ∼550–520 Ma deformation and upper-amphibolite-facies metamorphism (Fig. 7, Fig. 8). The presence of metre- to kilometre-scale mafic and ultramafic bodies in the Lufilian and Zambesi Belts, interpreted as rift-related ophiolite remnants (John et al., 2003, Johnson et al., 2005), document the existence of Neoproterozoic basins that opened after 890 Ma (880–735 Ma). Probably individual rift basins existed such as the Zambesi supracrustal sequence of southern Zambia, the Txitonga basin of northeastern Mozambique, and the Bukoban Basin within the Tanzanian Ubendian Belt (Johnson et al., 2007, Bjerkgard et al., 2009 and references cited therein) and were later dismemberedduring collision between Congo–Tanzania–Bangweulu and Zimbabwe–Kalahari Cratons. Ocean closure and crustal thickening at ∼530 Ma is constrained from biotite-staurolite-kyanite schists, whiteschists and eclogites that record the highest pressure conditions of all rocks considered in this paper (up to 2.7 GPa and 740 °C: Ring et al., 2002, John et al., 2003, John et al., 2004) (Fig. 7). High-pressure conditions are recorded in rocks with MORB geochemistry as early as 595 Ma (Sm/Nd data: John et al., 2003). They imply a low thermal gradient and a burial depth of ∼90 km during subduction of an oceanic tract. The steep decompression path (isothermal decompression: Fig. 7) is interpreted asthe result of exhumation following crustal thickening at ∼530 Ma. The distribution of high-pressure remnants (Fig. 8) shows that most eclogites are located next to prominent shear zones. They align with the Mugesse Shear Belt that parallels the trends of major Ubendian terranes (Boniface et al., 2012) and the Mwembeshi Shear Zone, separating the Southern Irumide Belt from the Irumide Belts *sensu stricto*. This suggests that transcurrent motion was the dominant style of Neoproterozoic to Cambrian deformation within Zambia and Malawi. Ring et al. (2002) proposed a model of continuously evolving transcurrent motion that incorporated eclogites during its late stage. In their model, general E-transport of nappes partitioned along the prominent shear zones into combined strike-slip and NEtectonic transport along the Ubendian margin (Mugesse shear zone), and strike-slip combined with SE transport along the Mwembeshi shear zone. Similarly, Dirks et al. (1999) and Johnson et al. (2005) described the Lufilian–Zambesi Orogen as a doubly-vergent orogen with fan like thin- and thick-skinned thrusting (Fig. 9).

It is noteworthy that overall NW-displacement in north Mozambique was coeval with E-tectonic transport in adjacent Zambia and Malawi. This requires the existence of a mega-scale shear zone or fault between both domains. The Chilwa-Mavago Corridor (Ueda et al., 2012), a high-strain shear belt that extends from southern Lake Malawi northward to Tanzania, might represent such a boundary (Fig. 8, Fig. 9).

#### 5.2.2 Styles of nappe assembly within northern segments (Tanzania, Kenya)

The Usagaran Belt and the Western Granulite Belt are structurally sandwiched between the Tanzania Craton below and the Eastern Granulite Nappe above (Fig. 4). Both belts show evidence of Kuungan deformation, but their metamorphic grades are different. In the Usagaran Belt, well preserved Paleoproterozoic ages from the granitic basement and the ∼1900 Ma old metasedimentary cover (Ndembera Group) testify to mild Kuungan overprint (e.g., Sommer et al., 2005a, Thomas et al., 2013). Sparse evidence of early Cambrian low-temperature deformation and metamorphism is found in the thrust contact at the Tanzania Craton margin. Atthis margin, the metasedimentary Konse Group (Mruma, 1995) and the Usagaran basement including Paleoproterozoic eclogites (Möller et al., 1995) were thrust onto the Tanzania Craton in a thin-skinned tectonic style (Reddy et al., 2003, Reddy et al., 2004, Fritz et al., 2005) (Fig. 8, Fig. 9). Discordant U–Pb rutile data derived from retrogressed eclogite (Möller et al., 1995) fall on a reference line intersecting the Concordia curve at 2010 Ma and 500 Ma. The isotopic composition of the rutile fractions reflects only partial isotope homogenization during the Kuungan metamorphic event, indicating that the temperatures attained during that time did not exceed the closuretemperatures of rutile of ∼430 °C (Reddy et al., 2003). K/Ar data (Gabert and Wendt, 1974) and ^40^Ar/^39^Ar data (Reddy et al., 2004) of biotite and muscovite derived from the craton margin and Usagaran Belt range from ∼3200 to 500 Ma and also point to incomplete resetting during the Kuungan orogeny. Although micas commonly have a component of excess ^40^Ar, the youngest muscovite ^40^Ar/^39^Ar date at 535 Ma has been interpreted as a maximum age for greenschist-facies metamorphism and deformation (Reddy et al., 2004). Gabert and Wendt (1974) noted that within the 3200–500 Marange cooling ages display a general younging trend away from the Tanzanian Craton, reflecting increasing late Neoproterozoic thermal resetting southeastward. This would imply eastward deepening of the basal detachment above which Kuungan thin-skinned nappes were displaced (Fritz et al., 2005) (Fig. 9).

Apart from lithologic differences, the boundary between the Usagaran and Western Granulite Belts is defined by the first occurrence of unambiguous late Neoproterozoic to Cambrian cooling ages (Gabert and Wendt, 1974). At this boundary, the structural style changes from localized semi-brittle to distributed ductile fabrics. Both indicate top-to-the west displacement at elevated temperatures, i.e. upper greenschist- to amphibolites-facies metamorphic grade (Tenczer et al., 2007). Northward, the Usagaran Belt thins out and Western Granulites are in direct contact with the Tanzania Craton. Discordant U–Pb zircon data from this northern boundary intersect the Concordia at ∼2600 Ma and ∼550 Ma and concordant U–Pb monazite ages of ∼558 Ma provide the best estimate for the local Kuungan metamorphic event (Tenczer et al., 2012).

Unfortunately, neither the regional extent of the Western Granulites nor the age of the convergent tectonics are known with certainty, leaving this crustal domains as one of the least understood crustal domains in the EAO. Ages between 641 and 633 Ma for peak metamorphism (Sommer et al., 2003, Vogt et al., 2006) were thought to characterize the Western Granulites (Fritz et al., 2005), but this assumption was challenged by Cutten et al. (2006) who derived significantly younger estimates between 549 and 535 Ma. Comprehensive synopsis of published data support indeed the idea that the main metamorphic and tectonic imprint within the Western Granulites was younger and occurred during the Kuungan Orogeny (Maboko, 2000, Cutten et al., 2006, Rossetti et al., 2008, Feneyrol et al., 2010, Tenczer et al., 2012).

Structures within the Western Granulite Belt display west-directed, high-vorticity, non-coaxial flow compatible with crustal thickening during emplacement onto the Tanzania Craton and the Usagaran Belt, respectively. The hangingwall boundary, i.e., the contact with the Eastern Granulites, is defined as a ductile to semi-brittle shear zone withnorthwestward nappe emplacement at ∼550 Ma (Rossetti et al., 2008). This shear zone cuts the ∼640 Ma old internal fabric within the Eastern Granulites. The thrust contact between Western and Eastern Granulites was folded by ongoing E–W displacement. The magnitude of nappe translation was apparently higher in northern Tanzania and southern Kenya than farther south. In the north, where Eastern Granulite rocks come close to the Tanzania Craton as the Usagaran and Western Granulites are entirely overthrust (Fig. 8, Fig. 9). Clockwise pressure–temperature evolutionary paths within the Western Granulites show a pronounced trend of isothermal decompression (ITD), which differs from IBC reported for the Eastern Granulites (Fig. 7). In contrast to slow cooling of the Eastern Granulites, theWestern Granulites rapidly cooled after the ∼560 Ma Kuungan metamorphic peak as a result of thrusting and exhumation (Fritz et al., 2009).

Kuungan metamorphism and deformation is also present in the East Galana terane (Hauzenberger et al., 2004) in southeastern Kenya, which is considered part of Azania (Fig. 8, Fig. 9). The terrane contains high-grade rocks such as migmatic biotite-plagioclase and hornblende gneisses, garnet amphibolites, thin quartzites, and coarse-grained marbles that experienced peak metamorphic conditions of ∼800 °C and 0.75–0.95 GPa at 580–550 Ma (Hauzenberger et al., 2004, Hauzenberger et al., 2007). Structures include E- to NE-vergent recumbent folds andtop-to-the NE shear fabrics, compatible with eastward thrusting. These structure indicate a thrust direction opposite to that recorded from Kuungan structures elsewhere in SE Africa but consistent with structures from Archean Madagascar.

#### 5.2.3 Styles of nappe assembly within eastern segments (Madagascar)

Central Madagascar, between the Ranotsara shear zone in the south and the Andraparaty thrust in the north (Fig. 8), is composed of distinctive Archean crustal blocks; the eastern Antongil and Masora Cratons and the central Antananarivo Craton (e.g., Schofield et al., 2010, De Waele et al., 2011 and references cited therein). The age of regional deformation and metamorphism throughout Madagascar is debated. Evidence for pre-630 Ma deformation is widespread (Nédélec et al., 2000, Nédélec et al., 2003, Collins et al., 2003a, Collins et al., 2003c, Fernandez et al., 2003, Tucker et al., 2011, Key et al., 2012), but older events were commonly strongly overprinted and obscured during the Kuungan Orogeny (560–530 Ma). Separate evidence of polyphase tectonothermal events is provided by three major age groups of magmatic rocks in the Antananarivo Block. These include the 855–720 Ma Imorona-Itsindro and the 634–565 Ma Kiangara magmatic suites, each of which were associated with metamorphism and deformation. (e.g. Goodenough et al., 2010 and references therein; De Waele et al., 2011, Key et al., 2012).

The Antananarivo Block is juxtaposed against the Kuungangreenschist- to lower-amphibolite-facies Antongil Craton (or Antongil– Masora Craton) along the Betsimisaraka suture. A continuation of this suture may be the Cambrian (∼535 Ma) high-temperature–high-pressure metamorphic Palagat–Cauvery shear system of southern India (Collins et al., 2007, Collins et al., in press, Plavsa et al., 2012). The Anaboriana–Manampotsy belt (Fig. 9; De Waele et al., 2011) (Ambodiriana Group after Fitzsimons and Hulscher, 2005) coincides in large portions with the Betsimisaraka suture and was metamorphosed to granulite-facies conditions at 541–520 Ma (Fitzsimons and Hulscher, 2005). Recent data, however, suggest a more complex history for theAnaboriana–Manampotsy belt involving amalgamation of the Antananarivo and Masora Cratons at about 820 and 740 Ma and later collision of the Antongil Craton with the amalgamated Antananarivo–Masora Cratons at ∼560 Ma (Goodenough et al., 2010, Key et al., 2012).

A series of structurally overlying allochthonous belts occur within the north-central Antananarivo Craton, collectively termed the Tsaratanana Complex or Tsaratanana Sheet (Collins et al., 2003a) (Fig. 9). This complex is interpreted as a lower crustal fragment of a middle Neoproterozoic continental magmatic arc thrust eastward over the Antananarivo Block between 530 and 500 Ma. A mylonite zone with top-to-the E displacement separates the Tsaratanana Sheet from the underlyingAntananarivo Block. Deformation initiated at amphibolites- to granulite-facies conditions, ongoing E–W shortening and coeval cooling (∼501 Ma) was accommodated by upright folds that refold previously formed thrust-related fabrics (Goncalves et al., 2003) (Fig. 9). Superposition of events gave rise to complex fold interference patterns and formation of large-scale dome and basin structures. Given the high-density contrast between the mafic gneisses of the Tsaratanana Sheet and the underlying less dense granites and gneisses of the basement, Goncalves et al. (2003) suggested that such dome-and-basin pattern could result from a combination of fold-interference structures with gravitational instabilities.

The N–S trending Angavo Shear Zone is another structural element that evolvedapproximately coevally with thrust fabrics (Fig. 8). It has been described as a 1000 km long and 40 km wide zone of flattening (coaxial strain) that developed between 549 and 500 Ma (Grégoire et al., 2009, Raharimahefa and Kusky, 2010). Metamorphic conditions reached low-pressure granulite-facies conditions, likely as result of heating due to melts and/or fluids ascending along the shear zone (Grégoire et al., 2009). Time equivalence of the Angavo Shear Zone with overall shortening within the Antananarivo Craton suggests strain concentration along the Dharwar Craton margin during the waning stage of Gondwana consolidation. The Angavo Shear Zone probably nucleated along a rheological discontinuity, likely corresponding to the eastern border of the Antongil–DharwarCraton (Grégoire et al., 2009).

Western Madagascar comprises a nappe assembly composed, in part, of the Itremo Group and the Ikalamavony Domain (Fig. 8, Fig. 9; Tucker et al., 2007, De Waele et al., 2011) or alternatively named, theAmborompotsy Group (Fitzsimons and Hulscher, 2005). Both Itremo Group and Ikalamavony Domain contain Mesoproterozoic to Neoproterozoic metasedimentary rocks thrust over, and imbricated with, the Antananarivo Block (Collins and Windley, 2002). The eastern part of the nappe assembly includes an important package of much younger clastic metasedimentary rocks which Cox et al. (2004) termed the Molo Group (Fig. 5). The Molo Group yields detrital zircons as young as ∼623 Ma and is therefore interpreted as having been deposited between ∼623 and ∼523 Ma, the age of metamorphic rims. The Itremo Group is split by the Itremo Shear Zone (Fig. 8) into an eastern, greenschist-facies segment that preserves N–S trending folds and eastward emplaced thrusts. A two-phase tectonic evolution is proposed by Fernandez et al. (2003) and Nédélec et al. (2003) for this segment (Fig. 9). During the first phase, some time between 720 and 570 Ma but prior to 565 Ma, the Itremo Group sediments were incorporated into a fold-and-thrust belt and transported toward the N and NE over the Antananarivo Block. During the second phase, around 550–530 Ma (e.g. Fernandez et al., 2003, Berger et al., 2006, Giese et al., 2011), both the Itremo Group and underlying Antananarivo Block, including the previously formed thrusts, were refolded into large-scale N–S trending folds with steeply dipping axial planes. The western Itremo segment and the overlying Ikalamavony Domain experienced granulite-facies metamorphism at ∼540 Ma. Zircons from the Ikalamavony Domain yield a near-concordant U–Pb age of 541 Ma, and are interpreted as having grown during granulite-facies metamorphism (Fitzsimons and Hulscher, 2005).

Fitzsimons and Hulscher (2005) argued that the Itremo Group/Ikalamavony Domain and the Antogil Craton(Dharwar Craton) were on opposing sides of the Mozambique Ocean prior to consolidation. In their view the Antananarivo and Antogil Craton collided first, as evident from relict ages older than 650 Ma. Later, at ∼550 Ma, the Itremo-Ikalamavony rocks collided with the Antananarivo Block, thereby causing a strong thermal overprint across all Madagascar. If so, the Mozambique suture may be traced from the Galana Shear Zone of southern Kenya south to an unexposed area west of the Ikalamavony Domain and then to the eastern margin of the Vohibory Domain of southern Madagascar (Fig. 8).

Madagascar south of the Ranotsara shear zone, and east of the Vohibory Domain comprises the Androyen and Anosyen Domains (Fig. 4), separated by N–S trending high strain shear zones (Martelat et al., 2000, De Wit et al., 2001). The Androyen Domain experienced two periods of metamorphism, one between 620 and 600 Ma at conditions of >835 °C and >0.7 GPa, and another between 570 and 530 Ma at lower pressures (∼0.4–0.5 GPa). The Anoysen Domain was metamorphosed to granulite-facies conditions before 545 Ma and preserves some of the highest-grade metamorphic rocks in Madagascar (895–900 °C, 0.9–1 GPa) (Tucker et al., 2011). Martelat et al. (2000) envisaged the structural assembly of southern Madagascar as a result of ongoing E–W shortening during superposed deformation between 590–530 Ma (D1) and 530–500 Ma (D2). The D1 strain pattern is characterized by a subhorizontal foliation andmineral stretching lineation globally trending E–W. Shear criteria are coherent with folding and/or thrusting. The D1 structures were reworked during the D2 event that led to complete transposition of fabrics within the N–S oriented high-strain shear zones. The D2 bulk strain pattern was related to a transpressional regime during bulk horizontal E–W shortening. Strain partitioning resulted in formation of an anastomosing network of shear zones and formed dome and basin geometries in less deformed pure shear domains. Both D1 and D2 finite strain patterns evolved coevally with high-temperature granulite-facies metamorphism. Martelat et al., 1999, Martelat et al., 2000 proposed a pressure gradient across the southern belts ranging from ∼1.2 GPa in the southwest to a minimum ∼0.5 GPa in the east, an interpretation challenged by Jöns and Schenk (2011).These regional pressure differences were explained by differential exhumation along theN–S shear zones during D2 transpressive compression amounting to at least 20 km of vertical motion.

### 5.3 Synopsis of crustal thickening in the southern Mozambique Belt

The East African and Kuungan orogenies climaxed ∼620 and ∼550 Ma, respectively, with both phases recording E–W to NW–SE shortening directions (Fig. 8). The polarity of thrusting, however, was different. In Tanzania and Mozambique thrusts were emplaced westward onto the Zimbabwe–Tanzania–Congo cratonic foreland; in Madagascar, nappes were emplaced eastward onto the eastern Antogil–Masora–Dharwar Cratons. Two-phase thrusting in Tanzania and Mozambique resulted in refolding of earlier formed fabrics and thrust planes such as the basal contact of the EGCD (Fig. 8, Fig. 9). In Madagascar, polyphase thrusting is evident from out-of-sequence thrusts within the Ikalamavony-Itremo domain. Older thrusts emplaced the low-grade Itremo suite onto the Antananarivo Block. Later, high-grade Itremo equivalents and the Ikalamavony suite were farther transported eastward onto the Antananarivo Block (Fig. 9).

The older East African Orogeny, manifested in the EGCD and the Vohibory Complex, may be classified as a hot- to ultra-hot orogen reaching syn-tectonic temperatures up to 1000 °C. Isobaric cooling textures indicate that the melt-infiltrated lower crust cooled slowly and resisted significant exhumation. Similar high-temperature (HT) to ultra-high temperature (UHT) granulite-facies metamorphic belts developed worldwide in the Neoarchean toCambrian. The belts have in common near-isobaric cooling and large crustal residence times and correlate broadly with amalgamation of continental crust into supercratons and supercontinents (Brown, 2006). Many Neoproterozoic–Cambrian HT–UHT metamorphic belts appear to have developed in settings analogous to modern back-arcs that were closed and inverted during crustal aggregation and formation of the Gondwana supercontinent (Brown, 2007). Those settings are characterized by thinned lithosphere that is generally hotter than lithosphere associated with the current destruction of the Pacific Ocean on modern Earth. It is envisaged that the inherent weakness of the lithosphere in a hot thermal regime in the East African Orogeny, probably induced by previous extension, inevitably localized magmatism and deformation contemporaneously with granulite to UHT metamorphism. The presence ofcontinuously produced sub-lithospheric melts weakens the overlying lithosphere. As a consequence, plates become weak, lose coherency and do not produce self-sustaining one-sided subduction but produce large areas of magmatic underplating and sagduction.

The Kuungan orogenic phases in Madagascar, Tanzania and Mozambique share the same characteristics, but have opposite vergences. The crust was doubly-thickened but pressure–temperature paths (Fig. 7) suggest a lower thermal gradient compared to the EGCD. Conversely, the overall viscosity was probably higher and, as a consequence, localized thrusts and non-coaxial deformation zones developed. Topography grew during shortening, and isothermal decompression textures evolved during rock exhumation. Shear localization into individual thrust surfaces was most pronounced where thrusts propagated toward shallow-level foreland units such as the Ponta MessuliComplex in NW Mozambique or the Usagaran Complex in Tanzania (Fig. 9). Displacement trajectories on the African side are orthogonal to their cratonic forelands but curved in eastern Madagascar (Fig. 8). Here, E-displacement is increasingly transferred to coaxial E–W shortening and N–S stretch within the Angavo shear domain. In Madagascar, the Dharwar Craton (Antogil and Masora Cratons) may have acted as a stiff, rigid back-stop forcing rock particle into orogen-parallel flow.

The Irumide–Lufilian Belt is considered a true subduction-type orogen with eclogites indicating downgoing slabs reaching pressures of 2.3 GPa and low thermal gradients (Fig. 7). Eclogite boudins are embedded within supracrustal rocks and domains of much lower metamorphic overprint, suggesting that they wereincorporated along discrete shears. Dominant strike-slip motion and fan-like thin- and thick-skinned flower structures (Dirks et al., 1999, Johnson et al., 2005) define the orogen as a transcurrent belt with limited overall thickening.

## 6 Orogen decay and post-accretionary modification in SE Africa and Madagascar

The late-tectonic evolution in SE Africa and Madagascar is deduced from cooling ages; the location and sedimentary record of post-accretionary basins; the presence of late- to post-tectonic granitoids; and post-accretionary fault patterns. Ages reported here are either interpreted as mineral-formation ages or cooling ages below relevant retention temperatures of specific isotopes in minerals. U–Pb and chemical Th–U–Pb ages from zircon and monazite have high closuretemperatures exceeding 800 °C but monazite may also grow within a wide range of temperatures, from diagenesis to upper granulite-facies metamorphic conditions. ^40^Ar/^39^Ar data and Rb–Sr mineral ages reflect cooling between ∼500 °C (hornblende) and ∼300 °C (mica); for fission track (FT) partial annealing temperatures range between ∼300 °C and 100 °C for individual minerals (sphene, zircon, apatite) (e.g., Schmitz and Bowring, 2003, Reiners and Brandon, 2006). For simplicity, the sedimentary basins are divided into marine and terrestrial basins, and further divided by reference to their site of deposition into intramontane and foreland basins. Granitoids are classed as “late tectonic” either because they are discrete intrusions along late tectonic structural elements or are circular plutons discordant tothe regional fabric.

### 6.1 Madagascar and Mozambique

**Fig. 11:**
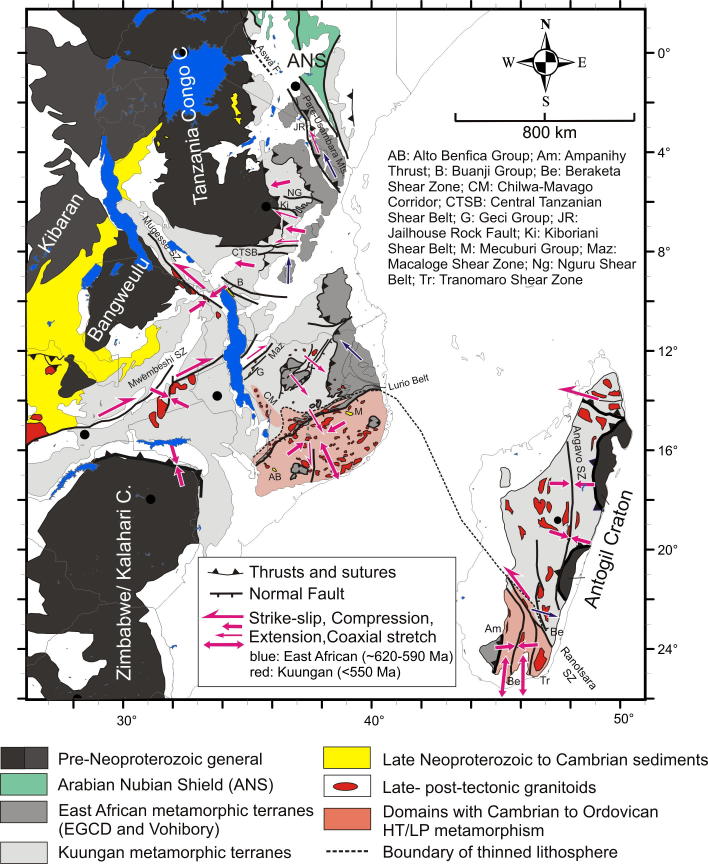
Late tectonic structures, late- to post-orogenic granitoids and distribution of Cambrian to Ordovician high-temperature low-pressure metamorphism within SE Africa and Madagascar. For further information see text.

Apart from the Betsileo Shear Zone between the Itremo Sheet and the Antananarivo Block (Fig. 11) with top-to-the-east sense of shear (Collins et al., 2000), the late-Neoproterozoic to Cambrian tectonic evolution of Madagascar is notable for the apparent absence of significant extensional structures such as low-angle or high-angle normal faults. Shearing at 630–561 Ma in the Betsileo Shear Zone defines an extensional period prior to Kuungan collision between 560 and 510 Ma; all other late tectonic structural elements comprise shear zones and faults that evolved within strike-slip or compressional regimes.

Left-lateralshearing along the NW-trending Ranotsara shear zone occurred between 550 and 530 Ma, followed by extension-related 530–520 Ma metamorphism. The lower crust cooled below ∼400 °C (De Wit et al., 2001; Schreurs et al., 2010) between 520 and 490 Ma. Schreurs et al. (2010) noted that lithologies, tectonic foliations, and axial trace trajectories of major folds can be followed N–S across most of the Ranotsara Zone and show only a marked deflection along its central segment. On this basis, they concluded that the Ranotsara Zone is not a megascale intracrustal ductile strike-slip shear zone that crosscuts the entire basement of southern Madagascar but is a prominent NW–SE trending brittle fault along most of its length. All other shear zones, including the AngavoShear Zone of central-northern Madagascar and the Ampanihy, Beraketa and Tranomaro shear zones of southern Madagascar, trend north–south (Fig. 11). The Angavo Shear Zone was active around 561–532 Ma (Grégoire et al., 2009, Raharimahefa and Kusky, 2010) and the southern shear zones around 500–440 Ma (Berger et al., 2006, Martelat et al., 2000, Giese et al., 2011). All evolved during bulk E–W compression and display high-temperature coaxial N–S stretch with a minor component of lateral shear (Martelat et al., 2000). Many shear zones have been identified as pathways for magmas and enhanced fluid flow from depth (Raharimahefa and Kusky, 2010).

An Ordovician low-pressure high-temperature event (Fig. 7) limited to southern Madagascar (490–440 Ma: Berger et al., 2006; 480–450 Ma: Giese et al., 2011) has been identified that post-dates the ∼550 Ma Kuungan collisional type high-temperature, high-pressure granulite-facies metamorphism. The low-pressure high-temperature conditions reflect crustal thinning after major ductile deformation (Giese et al., 2011). The waning stage of the Kuungan Orogeny in Madagascar was accompanied by emplacement of numerous granitoid plutons. The Maevarano magmatic suite of northern Madagascar consists largely of granitoid intrusions, which were emplaced between ∼537 and 522 Ma (Goodenough et al., 2010). A similar-aged magmatic event is recognized in central Madagascar, where metamorphism on the Angavo Shear Zone occurred at ∼550 Ma and was followed by magma emplacement at ∼550–530 Ma (Grégoire et al., 2009). The suites have been interpreted as resulting from lithospheric delamination associated with extensional collapse of the orogen (Goodenough et al., 2010). The authors argue that plutons of the Maevarano Suite are commonly associated with ductile shear zones that developed during extensional collapse; distinct extensional shears, however, have not been described so far. Instead, numerous E–W compressive structures extending to the Ordovician have been reported (e.g., Martelat et al., 2000).

In Mozambique,deposition ages of the Mecuburi and Alto Benfica sedimentary groups in the Nampula Block offer an excellent possibility to constrain the age of late metamorphism and deformation. The rocks of these groups (Fig. 11) comprise sequences of meta-psammite and meta-conglomerate interpreted as proximal, continental-fluvial sediments deposited in small, possibly intramontane, fault-controlled basins. The maximum depositional age of the Mecuburi Group derived from U–Pb ages of detrital zircon is 530 Ma. Clastic rocks were likely derived from the adjacent Lurio Belt and Cabo Delgado Nappe complex (Thomas et al., 2010). The Alto Benfica Group was deposited later than 530 Ma and records input from Mesoproterozoic and Paleoproterozoic sources. The sedimentary sequences were deposited in the course of exhumation after the Kuungan collision phase at∼550 Ma and were themselves deformed and metamorphosed at ∼500 Ma, concurrent with the emplacement of late-tectonic granitoids (Thomas et al., 2010). High-temperature/low-pressure metamorphism is evidenced from sillimanite-bearing rocks.

The Geci Group, another sedimentary package in northern Mozambique (Fig. 11), comprises remnants of a marine carbonate platform deposited upon the Unango Complex between ∼630 Ma and 585 Ma (Melezhik et al., 2006). Sedimentation postdates internal deformation of the Cabo Delgado nappes and probably relates to an early, pre-Kuungan extension phase (Viola et al., 2008). Crustal thickening and formation of high-pressure granulites during theKuungan Orogeny, between 570 and 530 Ma (Engvik et al., 2007), was followed by a second extension phase (Viola et al., 2008) and the intrusion of 533–486 Ma post-collisional granitoids (Marrupula suite; Jacobs et al., 2008, Bingen et al., 2009, Macey et al., 2010, Ueda et al., 2012). The volume of late-tectonic felsic magmatism is minor in the northern part Mozambique but increases markedly southwards along the Lúrio Belt and in the Nampula Block. This trend implies widespread crustal melting within and south of the Lúrio Belt, coeval with southeastward extension along the Lurio Belt and within the Nampula Block after ∼530 Ma (Viola et al., 2008, Ueda et al., 2012). The Chilwa-Mavago Corridorforms a similar N–S domain of extensive late-tectonic granitoid magmatism along the east shore of Lake Malawi (Ueda et al., 2012). Detailed studies are not available, but this corridor may define a domain of crustal weakness in northern Mozambique.

In summary, it is apparent that southern Madagacar and the Nampula Block share similar features. Both areas manifest widespread Cambrian to Ordovician (∼518–440 Ma) low-pressure, high-temperature metamorphism that postdates the Kuungan collisional type metamorphism. Late-tectonic N–S extension operated during ongoing E–W shortening; and large amounts of post-tectonic granitoid melts in both areas are inferred to have been generated by delamination of the lithosphere (Goodenough et al., 2010, Ueda et al., 2012).

### 6.2 Zambia, Malawi and southern Tanzania

The Irumide and Ubendian Belts as well as northernmost Mozambique and southernmost Tanzania are dominated by late-orogenic strike-slip tectonics (Fig. 11). Major faults are the dextral Mwembeshi shear system of Zambia and Malawi and the sinistral Mugesse shear system in the Ubedian Belt (Ring et al., 2002). Nearly all undeformed, post-tectonic granitoids in the region, dated between 510 and 474 Ma, lie between or along these shear zones (Johnson et al., 2006). The Mugesse shear zone exhibits a prolonged history with Paleoproterozoic dextral displacement (Boven et al., 1999) and Early Cambrian sinistral shear. The age of sinistralshearing is constrained by early Cambrian sediments (Buanji sediments) affected by sinistral strike-slip and a suite of late- to post-tectonic, NW-aligned, granitic to syenitic rocks ranging, in northern Malawi, from 490 to 440 Ma (Ring et al., 2002). Both major shear zones evolved in overall transpressive regimes combining components of strike-slip and thrusting. Hornblende ^40^Ar/^39^Ar cooling ages indicate that southward thrusting onto the Zimbabwe Craton and cooling below ∼500 °C had occurred by 507–491 Ma (Vinyu et al., 1999).

The low-grade sediments of the Geci Group in northernmost Mozambique crop out as small tectonic lenses within the Unago Complex. Interpretation of aeromagnetic maps suggest that the lenses are bounded by fault splays associated with the444 Ma old SW–NE trending Macaloge shear zone (Fig. 11; Bjerkgard et al., 2009). Diverse shear senses have been reported. However, from inspection of maps and correlation of the Macaloge shear zone with the greater Mwembeshi shear system, dextral shear as main deformation is suggested (Ring et al., 2002). Pyrite–chalcopyrite assemblages from gold-bearing quartz veins within this shear zone yield an imprecise age of 483 ± 72 Ma (Bjerkgard et al. 2009). Poorly exposed E–W trending shear zones in southern Tanzania, identified on aeromagnetic maps, may be regarded continuation of the Mugesse shear system. We consider these little known lineaments to be important because post-tectonic granitoids(<530 Ma) are absent to the north of them.

### 6.3 Central and northern Tanzania

Conspicuous differences between central and northern Tanzania and areas to the south are (1) an apparent absence of late-tectonic plutons (<530 Ma); (2) the presence of E–W trending shear zones; and (3) a scarcity of extensional fabrics. It is true that some N–S to NW–SE extensional fabrics occur in the Eastern Granulites (Fritz et al., 2005) correlating with those described from the Cabo Delgado Nappe Complex of Mozambique (Viola et al., 2008), but these fabrics record an extension phase after the ∼650–620 Ma East African Orogeny and prior to the ∼550 Ma Kuungan Orogeny. This agrees with ages fromthe Eastern Granulites that record general cooling below ∼500 °C between 630 and 580 Ma, well before the thermal peak and thrusting of the Kuungan Orogeny (Maboko et al., 1989, Möller et al., 2000 and references cited therein; Rossetti et al., 2008). In domains that experienced Kuungan deformation and metamorphism, late-tectonic extension is apparently absent.

In central Tanzania, E–W trending high-strain domains and shear zones are characterized by steeply dipping foliation planes that have high angles to the overall N–S gain of thrust-related structures. Syntectonic fabrics evolved close to peak metamorphic conditions of the units they cut. The Central Tanzanian Shear Belt (Fig. 11) can be traced from the southern margin of the Tanzania Craton eastward,cutting the low-grade Usagaran Belt and the high-grade Western Granulite Belt (Fritz et al., 2005, Tenczer et al., 2007). Along the craton margin and within the Usagaran Belt, brittle to semi-brittle fabrics are preserved; syntectonic temperatures increase eastward and reach granulite-facies conditions in the Western Granulites. Sinistral shear is well developed within rocks deformed at low-grade conditions. An increase of syn-tectonic temperatures coincides with an increase of N–S flattening (decreasing vorticity). The sense of shear is less clear within the northern Kiboriani and Nguru shear belts (Fig. 11). Dextral shear was suggested for the Kiboriani Belt (Fritz et al., 2005) whereas flattening without pronounced lateral motion is inferred for the Nguru Belt. Strongly metasomatized lithologies within the KiborianiBelt including yoderite whiteschists and sapphirine calcsilicates suggest that the steep belts provided pathways for fluids. Metamorphism associated with metasomatism is dated at 549–535 Ma (Cutten et al., 2006).

Rb–Sr biotite cooling ages with ∼300 °C blocking temperatures obtained from an E–W transect in northern Tanzania (immediately north of the Nguru Belt) gradually young eastward (∼531 to ∼470 Ma) away from the Tanzania Craton margin (Maboko, 2000). This gradient suggests that the eastern part of the Western Granulite Belt represents a deeper crustal level that cooled more slowly than the shallower western part. U–Pb data for apatites, titanites and rutiles from granulites in northern Tanzania yield concordant, generally overlapping dates between 547 and 509 Ma, which areinterpreted as cooling from 650–550 °C through 550–450 °C and 450–350 °C. The U–Pb ages suggest rapid cooling and exhumation during westward emplacement of Western Granulite nappes onto the Tanzania Craton (R.L. Rudnick, personal communication). Cooling below ∼400 °C at 517–511 Ma is also constrained from ^40^Ar/^39^Ar muscovite data derived from nearby Tsavorite gemstone deposits south of Arusha (Feneyrol et al., 2010, Feneyrol et al., 2013).

The E–W fault patterns of central Tanzania evolved close to the metamorphic peak between ∼550 and 530 Ma and differ in age from the Late Cambrian to Ordovician extensional and strike-slip faults of Mozambique, Zambia and Madagascar. The structures are interpreted as tear faults accommodating differing amounts of thrust displacement duringbulk E–W shortening (Tenczer et al., 2007). This explains different senses of shear within individual lineaments. Rooting of the faults and shear zones at the eastward-deepening basal decollement level of thrusts is compatible with eastward increase of temperatures during shear.

On the basis of its regional trend, a steep NW-oriented zone of sinitral faulting in northern Tanzania, along the western flank of the Pare Usambara Mountains (Fig. 11), is considered part of the southern ANS fault systems. This zone (the Jailhouse Rock Fault named after a locality close to Same village ) appears to be a continuation of the prominent Aswa Fault in Kenya and Uganda. Mafic to ultramafic rocks along the fault zone, including metagabbro, metapyroxenite and serpentinite, are either interpreted as ophiolitic cumulates (Bauernhofer et al., 2009) or mantle slivers. Ultramafic rocks are strongly hydrothermally altered and enclose small bodies of magnesite.

### 6.4 Summary of late-tectonic features in the southern Mozambique Belt

**Fig. 12:**
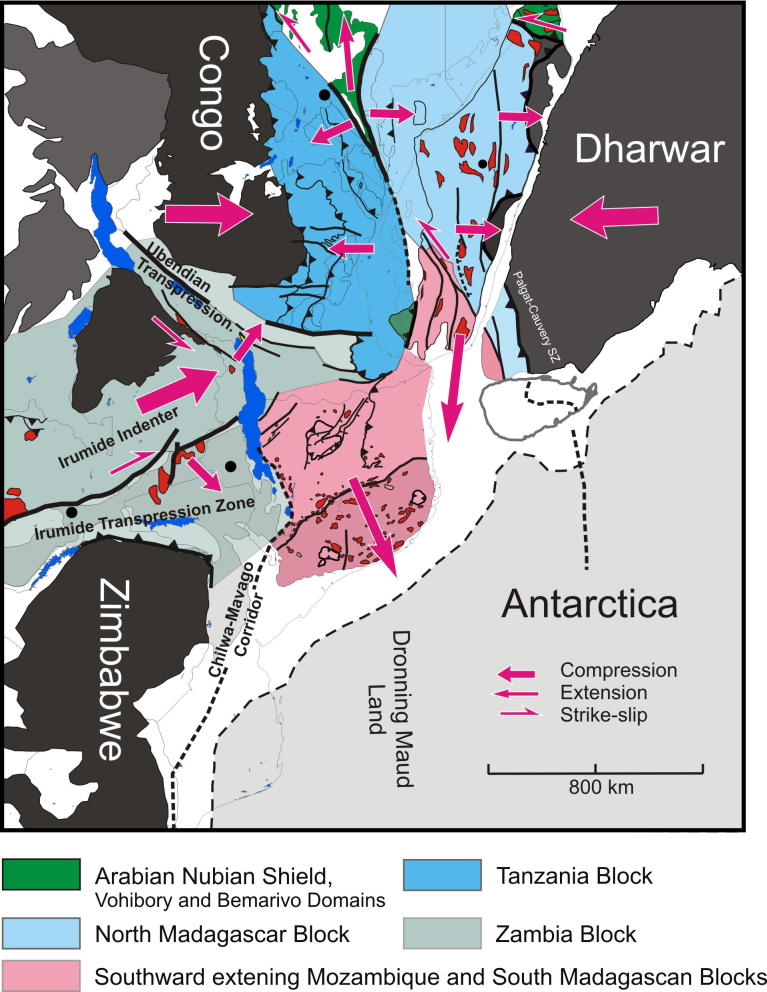
Crustal domains, here defined as coherent blocks with distinctive late tectonic (<530 Ma) structural, metamorphic and magmatic assemblages. Note that Madagascar, India and Antarctica are shown in positions before Gondwana break-off. Mozambique and southern Madagascar experienced southward extension concomitant with high-temperature low-pressure metamorphism and emplacement of vast granitoid melts. The Arabian Nubian Shield had northward motion that commenced at ∼600 Ma. Central Tanzania and northern Madagascar experienced orthogonal shortening resulting from convergence of Tanzania–Congo and Dharwar Cratons. The Zambia Block is viewed as eastward moving indenter (Irumide Indenter) with transpressional deformation concentrated along its margins. The boundary between the Zambia Block and the Mozambique Block might be represented by the melt invaded Chilwa–Mavango corridor that probably extends to the Natal embayment between South Africa and the Dronning Maud Land of East Antarctica.

Northern Tanzania and northern Madagascar share the common feature that faults and shear zones evolved through continuous E–W convergence. They evolved contemporary with ∼550 Ma Kuungan peak metamorphism. Tanzanian E–W striking shears, e.g., the 549–535 Ma Kiboriani Belt, are interpreted as tear faults accommodating different amounts of coeval thrust displacement. N–S shear zones in Madagascar (e.g., the 561–532 Ma Angavo Shear Belt: Grégoire et al., 2009, Raharimahefa and Kusky, 2010) accommodated prolonged E–W shortening.Both domains exhibit strong orthogonal shortening associated with thrusting onto their respective cratonic forelands, the Dharwar and Tanzana–Congo Cratons (Fig. 12).

Late-tectonic faults and shear zones in southern Madagascar, southern Tanzania, Mozambique, Malawi and Zambia have prolonged histories, extending from Cambrian to Ordovician times, and are associated with vast granite emplacement. In southern Madagascar and Mozambique, general southward extension and coeval high-temperature, low-pressure metamorphism younger than 530 Ma is considered as having resulted from lithospheric delamination during orogenic collapse (Jacobs et al., 2008, Viola et al., 2008, Ueda et al., 2012). Extension is seen as the result of retreating subduction along the southern Gondwana margin that in DronningMaud Land (Antarctica) generated coeval granulite-facies metamorphism (530–515 Ma: Jacobs et al., 1998, 2008) and extensional collapse granitoids (530 and 500 Ma) (Jacobs and Thomas, 2004). Contemporary NE motion of the Irumide Belt activated transcurrent movement and the emplacement of granitoid plutons along along the Mugesse and Mwembeshi shear belts. The southern Irumdide Belt and the Ubendian Belt are dextral and sinistral transpressional zones, respectively (Fig. 12) and the entire Irumide Belt may be regarded consequently as a NE-moving indenter with strong reworking along its margins.

## 7 Orogen decay and post-accretionarymodification in the Arabian–Nubian Shield

**Fig. 13:**
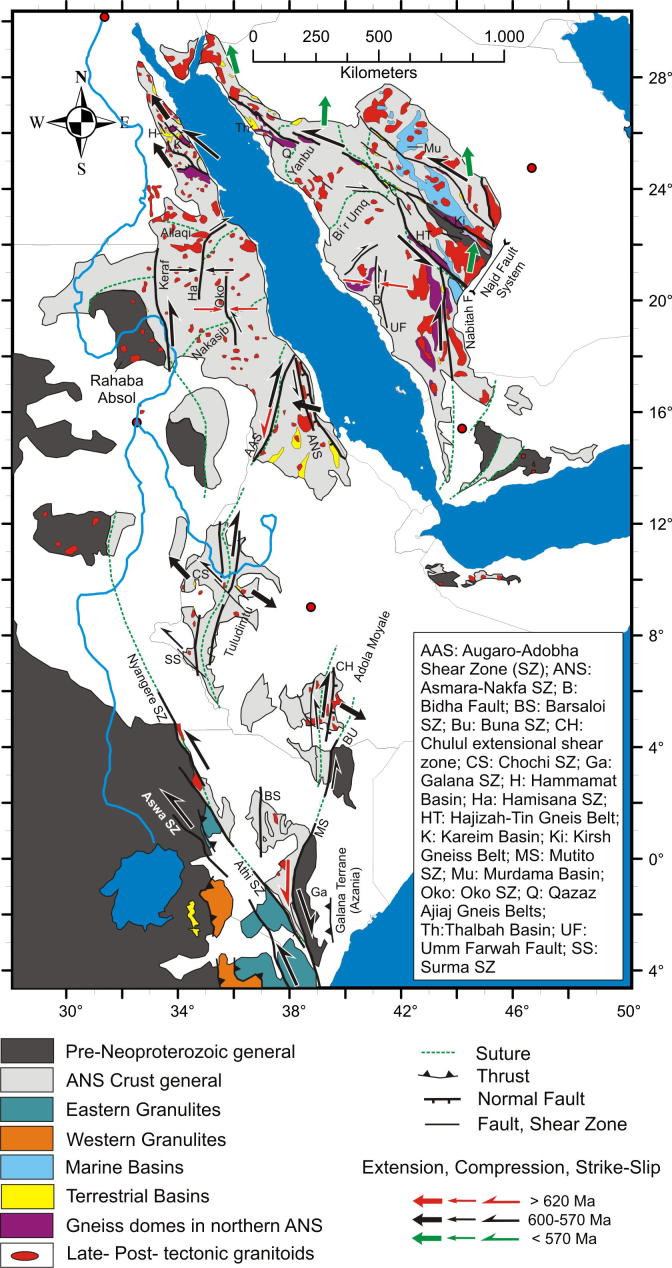
Late tectonic structures, late- to post-orogenic granitoids and distribution of sedimentary basins within the ANS. For further information see text.

Shear-zone patterns within the ANS occupy three different orientations with respect to ophiolite-decorated sutures. (1) In the domain between the southern ANS composite terrane and the Nakasib-Bír Umq suture, faults and shear zones strike north–south and parallel suture zones. Exceptions are the southwestern ANS boundary shears (Aswa and Nyangere shear zones) that strike NW. (2) Between the Nakasib-Bír Umq and Allaqi-Heiani-Sol Hamed-Yanbu sutures, fault zone are N–S oriented at high angles to the suture zones. (3) In the north and east the prominent Najd Fault system appears with NW-trending faults aligned roughly parallel to the Nabitah Orogenic Belt and cutting through the Bír Umq and Yanbu sutures (Fig. 2, Fig. 13).

### 7.1 Thesouthern Arabian–Nubian Shield

The Aswa and Nyangere shear zones, considered as extensions of the Ranotsara Shear zone of Madagascar (Martelat et al., 2000), are high-temperature sinistral shear zones (Shackleton, 1996, Bauernhofer et al., 2008a) with a strong component of coaxiality. The age of the Nyangere Shear zone is estimated at ∼593 Ma from imprecisely dated late- to post-tectonic granites cutting the Sekerr ophiolite suite (Ries et al., 1992). Polyphase shearing is recorded from the Galana Shear Zone, with early sinistral shear, again with a high component of coaxiality, and later dextral shear (Bauernhofer et al., 2008a). Here, the Eastern Granulites with 580–560 Ma cooling ages(^40^Ar/^39^Ar hornblende and biotite) are juxtaposed against the Galana terrane with ∼519 Ma cooling ages (^40^Ar/^39^Ar hornblende). The 519 Ma age is considered to date cooling below ∼500 °C, together with dextral strike-slip (Bauernhofer et al., 2008b). Divergent shear senses have been described from the Barsaloi suture where K/Ar biotite ages cluster around 550 Ma. Evidence for termination of shear is provided by a crosscutting dolerite dyke (Sinyai metadolerite), imprecisely dated 569 ± 126 and 593 ± 38 respectively (Key et al., 1989).

The Adola-Moyale and Tuludimtu ophiolite belts of Ethiopia (Fig. 5, Fig. 13) formed through prolonged oblique convergence with combined strike-slip and thrusting. Both sinistral and dextral shear senses have been described, which suggests superposed deformation or strong component of coaxiality. Most data, however, point to dextral shear as the dominant component of deformation (Tolessa et al., 1991, Worku and Schandelmeier, 1996, Yihunie and Tesfaye, 2002, Allen and Tadesse, 2003, Tadesse and Allen, 2005, Tsige and Abdelsalam, 2005). The final phase in the evolution of the Adola-Moyale Belt is characterized by combined dextral and southeastward normal-slip along the Chulul Shear Zone (Fig. 13), attributed to extensional collapse (Tsige and Abdelsalam, 2005). The ∼580 Ma age of extensional tectonics is constraint by a zirconage for granite emplaced during shearing. Numerous K/Ar and ^40^Ar/^39^Ar ages (muscovite and hornblende) range from 550 to 500 Ma (Tsige and Abdelsalam, 2005 and references therein). This interval is also marked by the emplacement of late- to post-tectonic granitoids associated with uplift and cooling at the end of the East African Orogeny in this area (Yihunie and Tesfaye, 2002). Post-accretionary sedimentary basins occur in two areas within and adjacent to the Tuludimtu Belt (Fig. 13; Allen and Tadesse, 2003). Up to 1000 m thick, undated successions of undeformed conglomerate–sandstone–shale sequences are regarded as molasse-type sediments, deposited in the course of gravitational collapse in the TuludimtuBelt. The sedimentary basins are elongated in a northwesterly direction and formed as pull-apart basins next to sinistral NW-striking shear zones (Fig. 13; Chochi and Surma Shear Zones). These strike-slip zones sinistrally offset all lithotectonic units and older shear zones in the Tuludimtu Belt. Gold, and base metal mineralization, deposited by hydrothermal fluids, accompanied this shearing event.

The most prominent Neoproterozoic shear belt in Eritrea is the western Augaro-Adobha Belt. Although sinistral strike-slip shearing was dominant, local fabrics indicate dextral slip during a later tectonic phase (Ghebreab et al., 2009). The sinistral Asmara-Nakfa Belt strikes NNW–SSE for ∼200 km, from south of Asmara to northernmost Eritrea and merges in the north with the NNE-trending Augaro-Adobha Belt. Juxtaposition of high-grade rocks(Ghedem Domain) with the central Eritrean low-grade volcanosedimentary sequences along a W-dipping low-angle mylonite zone was assigned to orogenic collapse (Ghebreab et al., 2005). Exhumation and cooling of high-grade rocks between 586 and 572 Ma (^40^Ar/^39^Ar hornblende plateau ages) and 576 and 563 Ma (^40^Ar/^39^Ar muscovite plateau ages) was correlated with formation of this extensional mylonite zone (Ghebreab et al., 2005). These ages agree with 587–564 Ma rutile ages from the Ghedem Domain, interpreted as cooling below ∼400 °C (Andersson et al., 2006). Immediately after or coeval with extension, sinistral strike-slip evolved in the Asmara–Nakfa Belt, coeval with granitic intrusions withages as young as 545 Ma (Tadesse et al., 1997). Deposition of sediments of uncertain stratigraphic age (Tambien Group) was connected with extension along low-angle detachments (Beyth et al., 2003). The sediments comprise slates, phyllites, greywackes, limestone and quartzitic dolomites deposited prior to emplacement of the cross-cutting late-tectonic Mareb granite dated 545 Ma (Tadesse et al., 1997) and ∼610 Ma (Avigad et al., 2007). The isotopic signature of carbonate rocks is similar with those of cap-carbonates formed during the Sturtian (740–720 Ma) glaciation (Miller et al., 2009). However, Miller et al.(2011) argued that the Tambien sediments were deposited in an intra-oceanic arc platform setting within the Mozambique Ocean, prior to ocean closure.

### 7.2 The central Arabian–Nubian Shield

The Keraf Suture defines the western margin of the ANS and formed during sinistral transpression in the course of final, oblique NW–SE collision between the Arabian–Nubian Shield and the Saharan Metacraton. The N- to NNW-striking shear zone deforms 620–580 Ma A-type granitoids along the northeastern part of the Saharan Metacraton (Abdelsalam et al., 2003).

Two N–S trending shortening zones post-date convergence along the Nakasib and Allaqi-Heiani sutures. The southern Oko shear zone (Fig. 13) offsets the780–750 Ma old Nakasib suture sinistrally by ∼10 km. It formed through three phases (Abdelsalam, 1994, Abdelsalam, 2010). The earliest was initiated by E–W shortening and produced N- to NNW-trending upright mostly tight to isoclinal folds. The intermediate phase culminated with the formation of strike-slip faults between ∼640 and 560 Ma. The youngest phase consisted of ongoing shortening and strike slip that produced E- and W-verging thrust sheets in a flower structure. Deformation of the Hamisana high-strain zone post-dated emplacement of ophiolites along the Allaqi–Heiani suture. Deformation was dominanted by pure shear in upper greenschist- to amphibolites-facies metamorphism (Miller and Dixon, 1992, Stern et al., 1989). Subsequent non-coaxial shearing took place at lower-temperatures and led to the formation of minorNE–SW-striking dextral faults. This dextral faulting is considered conjugate to the prominent NW–SE trending sinistral Najd fault system of the northern ANS (De Wall et al., 2001). Activity along the Hamisana belt may have begun as early as 660 Ma, culminating in intense thermal activity at ∼610–580 Ma. Deformation ceased before intrusion of undeformed, post-tectonic granites during this time (Stern et al., 1989). Overall, both the Oko and Hamisana high strain zones are characterized by pure shear expressed as E–W-shortening and strong N–S extension resulting in bulk constrictional strain (Abdelsalam, 2010, De Wall et al., 2001).

Similar N-trending structures are prominent within the Asir and Tathlith terranes south of the Bír Umq suture. In southwestern Saudi Arabia, these include the Nabitah, Umm Farwah, and Bidah fault zones (Fig. 13). The Nabitah fault between the Asir and Tathlith terranes is a dextral strike-slip system that underwent renewed slip after intrusion of late-tectonic 640 Ma granitoids (Stern and Johnson, 2010). Internal shear zones of the Asir terrane have both sinistral and dextral shear and are characterized by a strong E–W shortening component (Johnson et al., 2011 and references therein). The Umm Farwah Shear Zone extends for about 200 km N–S across the central part of the Asir terrane; it has S–C fabrics indicating both dextral and sinistral movements and is associated with moderate to strong folding about SSW-trending axes. Shearing is interpreted as the cause of crustal melting and emplacement of617 Ma old A-type granitoids. The Bidah Shear Zone has a sinistral sense of movement, the ealiest phase of which was marked by E–W compression and the development of N-trending folds and mineral lineations. The minimum age of shearing is constrained from plutons that intruded into sheared rocks at ∼635 Ma (Volesky et al., 2003). Northwesterly Najd faults prevail in the Hijaz terrane and obscured any north-trending fabric elements that may have been present (Fig. 2, Fig. 13).

### 7.3 The northern Arabian–Nubian Shield

Northwest-striking shear zones and faults of the Najd Fault System are the dominant structural elements within the Afif- Hijaz- and Midyan-terranes of northernwestern Saudi Arabia and the EasternDesert terrane of Egypt. The system consists of brittle–ductile shears in a zone as much as 300 km wide and more than 1100 km long, extending across the northern part of the Arabian Shield. The fault zone presumably continues across the concealed basement of the Arabian Plate into parts of India and the Lut block of Iran (Johnson et al., 2011). Shearing along the Najd Fault System is genetically linked with deposition of sediments, exhumation of gneiss domains, and emplacement of syntectonic granitoids.

Several types of sedimentary basins are present within the northern ANS. Deposition of volcanosedimentary assemblages as components of volcanic arcs occurred prior to 650 Ma. After 650 Ma, volcanosedimentary assemblages were deposited in post-amalgamation basins overlying newly amalgamated arc terranes. Post-amalgamation successionswith unconformable basal contacts include marine, mixed marine and terrestrial basins. Terrestrial basins concentrate in the northwest, suggesting a location more elevated and (or) farther from the sea than the marine basins in the east (Johnson et al., 2011).

Marine basins such as the Murdama Basin (Fig. 13) began to develop during and soon after the Nabitah orogeny (680–640 Ma) that marked suturing of the Afif terrane with oceanic ANS terranes to the west. The Murdama Group as the main constituent of the Murdama Basin is estimated to have been deposited between ∼650 and 620 Ma and is intruded by 650–570 Ma granitoid plutons. Gently plunging, open, upright, north-trending folds with locally vertical limbs are pervasive, indicating bulk E–W shortening, in part associated with sinistral shearing ontranscurrent Najd faults (Johnson, 2003). The abundance of marine deposits implies that large areas of the northeastern ANS were depressed below sea level during the late Cryogenian and Ediacaran.

Post-amalgamation terrestrial basins are concentrated along the northwestern part of the Najd Fault System. The largest terrestrial basin in Saudi Arabia is the Thalbah basin (Fig. 13) that contains sediments deposited between about 620 and 595 Ma (Johnson et al., 2011). In Eygpt, correlative terrestrial basins are known as Dokhan Volcanics and Hammamat Group. Whether both sequences are interfingering time equivalents or overlying successions is matter of debate. The Dokhan Volcanics, erupted between 630 and 592 Ma, vary in thickness from basin to basin, ranging from afew tens of metres to 1300 m (Eliwa et al., 2006). The Hammamat Group, deposited between about 600 and 580 Ma (Abd El-Rahman and Doig, 1987, Wilde and Youssef, 2000, Wilde and Youssef, 2002, Breitkreuz et al., 2010), ranges from about 4000 m thickness along Wadi Hammamat (Abd El-Wahed, 2009) to about 7500 m in the Kareim Basin (Fritz and Messner, 1999). We note that onset of deposition was likely not contemporaneous in all basins, suggesting the existence of individual depositional sites with variable sedimentary facies. Most Hammamat type basins are fault-bounded. Some are considered extensional basins that formed along extensional bridges of the Najd Fault shear system (Kareim Basin: Fritz and Messner, 1999, Abd El-Wahed, 2009, Fowler and Osman, 2013); some are strike-slip pull-apart basin (Shalaby et al., 2006). Relations between sedimentation, activity of faults, and exhumation of gneissic domes are well shown around the Kareim Basin in the Central Eastern Desert of Egypt (Fig. 13). Initial slow subsidence was associated with the formation of early sinistral strike-slip shear zones and the onset of exhumation of the southerly adjacent Sibai gneiss dome at ∼650 Ma. Terminal fanglomerate deposition at enhanced subsidence rate was associated with a later phase of sinistral shearing and general cooling of the gneiss dome below ∼500 °C at ∼620 Ma (^40^Ar/^39^Ar hornblende ages: Fritz etal., 2002). The sedimentary sequence was itself affected by shear tectonics and was later intruded by post-tectonic granites at about 580 Ma (Fritz and Messner, 1999). Other basins, such as the Hammamat Basin at Wadi Hammamat, record rapid hinterland uplift at about 595–588 Ma (Fritz et al., 1996, Loizenbauer et al., 2001) and were intruded by post-tectonic granites at ∼596 Ma (Andresen et al., 2009).

Typical for the northern ANS are gneiss domes that align with the Najd Fault system. In the Arabian Shield, late Cryogenian–Ediacaran gneiss crops out as relatively narrow northwest-trending gneiss belts that extend NW across the entire Arabian Shield (Fig. 13). Arabiangneiss belts such as the Kirsh gneiss belt, the Hajizah-Tin gneiss belt and Qazaz-Ajiaj gneiss belt are characterize by deformation that originated as ductile shearing under amphibolite-facies conditions and continued as brittle shearing. Vertical foliation and sub-horizontally plunging linetaions are frequent with a pronounced component of unidirectional stretch parallel to the NNW Najd Fault trend. Late tectonic plutons frequently intrude left-stepping extensional bridges, and small terrestrial basins evolved on down-faulted blocks or in pull-apart basins (Johnson et al., 2011). The Kirsh gneiss was exhumed between 645 and 550 Ma. Gneiss and pegmatite crystallized between 647 and 637 Ma and ^40^Ar/^39^Ar biotite ages indicate cooling below ∼300 °C at 557 Ma. The minimum age of deformation in the Hajizah–Tin gneiss belt isconstrained by post-tectonic granites dated at 592 Ma. Along the Qazaz-Ajiaj shear zone deformation continued at least until 575 Ma, the protolith age of incorporated granite gneiss (Johnson et al., 2011 and references cited therein). Late Cryogenian–Ediacaran mafic, felsic, and composite mafic–felsic dikes are widespread in the Arabian Shield but less frequent in the Nubian Shield. Such post-tectonic, overwhelmingly E–W striking dikes date between ∼590 and 545 Ma define cessation of Najd Fault shearing and are unambiguous evidence for N–S extension during the final phases of shield development.

Gneiss domes in Egypt have a different appearance because, in addition to strike-slip faults, they are frequently bordered by low-angle normal-faults. It should be noted, however, that the process forming the gneiss domes and surrounding shear zones is amatter of debate. The shear zone patterns have been interpreted as combined strike-slip and extensional shears that formed during exhumation of domes (e.g., Fritz et al., 1996, 2002) or as remnants of NW-directed thrusts (Andresen et al., 2010). Gneiss domes have either been interpreted as core complexes (e.g., Fritz et al., 1996, Blasband et al., 2000) or antiformal stacks formed during thrusting (e.g., Greiling et al., 1994). Nonetheless, there is agreement on significant NW–SE extension within the Eastern Desert Terrane of Egypt. The juxtaposition of low-grade volcanosedimentary sequences against high-grade gneisses along extensional shears suggests crustal-scale thinning by NW–SE extension, accompanied byintense magmatic activity (Blasband et al., 2000, Fowler and El Kalioubi, 2004, Fowler and Osman, 2009, Andresen et al., 2010). Geochronology suggests that extension and exhumation of gneiss domes, although probably diachronous across the region (Fritz et al., 2002), commenced around 620–606 Ma, as suggested by the oldest ^40^Ar/^39^Ar hornblende cooling ages and emplacement of extension-related granitoids (Fritz et al., 2002, Andresen et al., 2009). Deformation ceased prior to emplacement of discordant post-tectonic granitoids at about 580 Ma. This agrees with ∼585 Ma ^40^Ar/^39^Ar ages of white micas that grew during NW–SE extensional shearing and therefore directly date the shearingevent (Fritz et al., 1996).

Igneous activity was very important in the late Cryogenian–Ediacaran evolution of the northern ANS as plutonic rocks form 42% of the exposed surface (for details see Johnson et al., 2011). There is evidence of a temporal change in granitoid type, with a calc-alkaline assemblage at 645–615 Ma and strongly fractionated peralkaline, peraluminous, and related leucocratic granites emplaced at 585–570 Ma. The late Cryogenian–Ediacaran plutons are typically discordant with respect to already deformed and metamorphosed country rocks. Most lack penetrative deformation fabrics and have the general character of late- to post-tectonic intrusions. In the Nubian Shield, Lundmark et al. (2011) identified six pulses of magmatic activity at: (1)705–680 Ma; (2) ∼660 Ma; (3) 635–630 Ma; (4) 610–604 Ma; (5) 600–590 Ma: and (6) 550–540 Ma. The first three pulses are considered synorogenic, pulses 4 and 5 record exhumation of mid-crustal gneisses, and pulse 6 postdates orogeny.

### 7.4 Summary and discussion of late tectonic features in the Arabian–Nubian Shield

We call attention here to conspicuous differences in the structural evolution between the Arabian and Nubian parts of the northern ANS. The Najd Fault System of Arabia is best described as a wrench tectonic setting. Gneiss domains contain NW–SE stretched L and L–S tectonites; granites and sedimentary basins evolved within extensional bridges along releasing bends of the Najd shear system. Gneisses were exhumed along positive flowerstructures and basin sediments were deposited in adjacent small troughs. No low-angle extensional detachments have been reported from the Arabian Shield. By contrast, the Najd Fault System in the Nubian shield exhibits a strong component of vertical shortening with evidence of significant NW–SE extension shown by extensional faults adjacent to gneiss domes.

The distribution of sediments with marine facies in the northeastern Arabian Shield and terrestrial basins in the northwest may be interpreted in terms of variable exhumation and/or erosion. By the time of sedimentation of the marine Murdama Group (∼650–620 Ma) the eastern Shield was below sea level, whereas the northwestern Shield may have had a mountainous topography. This is indicated by coeval clastic sedimentation in the terrestrial Thalbah Basin (620–595 Ma) and in numerous intramontane Hammamat-type basins (600–579 Ma) in Egypt. Although notstrictly verified, ^40^Ar/^39^Ar cooling ages provide a weak trend with a 612–602 Ma age cluster in the northeastern part of the Arabian Shield and ages around 580 Ma in the northwest (Table 1 in Johnson et al., 2011). This may be taken as a suggestion of earlier, and probably slower, regional cooling and exhumation in the northeast than the northwest.

**Fig. 14:**
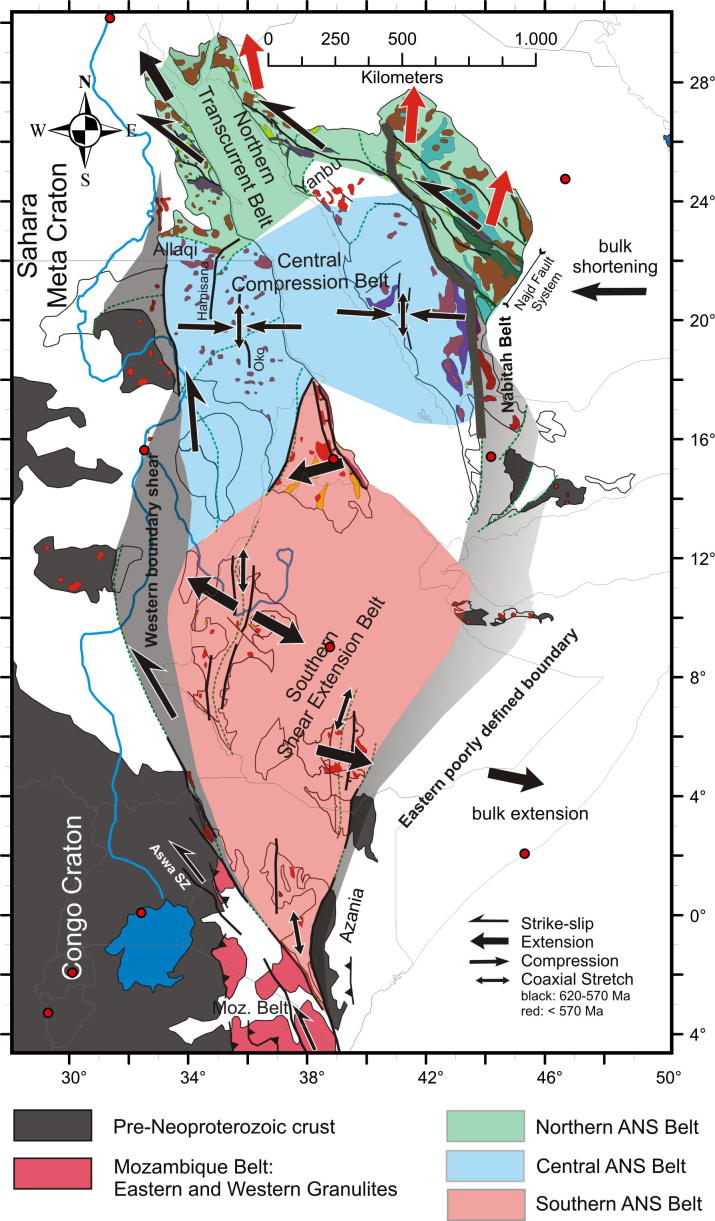
Crustal domains, here defined as coherent belts with distinct late tectonic structural, magmatic and sedimentary assemblages. Note the concentration of late tectonic granitoids, sinistral strike-slip shear zones and post-accretionary sedimentary basins in the northern ANS belt (compare with Fig. 13). The northern ANS is a transcrurrent belt that evolved through bulk W–E shortening during the Nabitah Orogeny releasing sinistral slip within the Najd Fault System. Later 570 Ma N–S extension was dominant. Internal portions of the central ANS belt evolved through orthogonal W–E convergence with respect to the southern Nabitah Belt and the Sahara Metacraton margin. It lacks significant strike-slip faulting, instead W–E shortening is evident from the Hamisana and Oko shortening zones. Sinistral strike-slip displacement concentrates along the Sahara Metacraton margin (western boundary shears). The southern ANS belt experienced NW–SE to W–E extension induced by gravitational collapse that occurred prior to final (<570 Ma) N–S extension in the northern ANS belt. For further information see text.

Summarizing data of orogen decay shows that different structural elements characterize individual portions of the ANS (Fig. 14). (1) The northern domain is dominated by the Najd Fault trend and the features described above. In short, it is characterized by NW-trending faults, extensive post-tectonic granitoid magmatism, occurrence of terrestrial and marine basins, and absence of high-grade metamorphism,except the gneiss domains. It is a transcurrent belt with significant NW stretching. Mafic–felsic dykes documenting ∼590 to 545 Ma N–S extension are widespread in the eastern Arabian Shield but less common in other areas. (2) The central domain comprising the Gebeit, Haya, Jiddah and Asir Terranes (Fig. 2, Fig. 14) experienced prolonged E–W shortening as seen in the Hamisana and Oko high strain belts. Prolate, unidirectional N–S stretching and E–W shortening is characteristic; otherwise no extension and few sedimentary basins occur in this area. The Nabitah Belt partly defines the eastern boundary between the central and northern domains. In its southern part E–W shortening prevails, whereas in the north sinistral strike-slip is dominant. This may be taken as evidence for partitioned displacement at the edges of earlier-formed arc terranes in the Arabian Shield (Afif, Jiddah, and HijazTerranes). (3) The southern domain exhibits the highest-grade metamorphism and coaxial N–S stretching, partly coeval with migmatite formation and granulite-facies metamorphism (Fig. 5). Few late-tectonic sedimentary basins and also few post-tectonic granitoids are exposed. Arguably, exhumation was greatest in the southern ANS, providing insight into a deeply eroded transcurrent arcs assembly. Significant E–W to NW–SE extension is attributed to orogenic collapse (Ghebreab et al., 2005, Tsige and Abdelsalam, 2005). Extension through orogenic collapse in the southern ANS is, most likely, not causally related to the contemporaneous N- to NW-extension in the northern ANS because the central domain with distinctly different late tectonic structural assemblages separates the northern from the southern domain (Fig.14).

Additional information on the late orogenic structure of the northern Arabia–Nubian lithosphere is provided from seismic refraction data (Gettings et al., 1986), from teleseismic receiver functions (Levin and Park, 2000, Al-Damegh et al., 2005), and thermomechanical modelling (Avigad and Gvirtzman, 2009). Levin and Park (2000) identified upper mantle shear zones in Eastern Arabia where the NNW-trending western Ar Rayn suture and the NW-trending Najd Fault system converge. Upper-mantle seismic N–S anisotropy is interpreted to reflect lattice-preferred fabric patterns of upper-mantle minerals formed during N–S extension after the assembly of the Arabian Shield. These authors hypothesized that a relatively strong mantle‘lid’ between the Moho and the Hales discontinuities migrated northward as an escape-tectonics ‘block’ during the last stages of terrane accretion in the Arabian Shield. Their data provide two types of information. The Najd Fault system is, at least in that region, a structure penetrating through the entire crust, although its nature at depth is poorly known. Further; late N–S extension, as also evidenced by the orientation of dyke swarms, is a lithospheric-scale process in the eastern Arabian Shield. Late-tectonic granitoids that constitute the most abundant rock type and, on the basis of seismic-refraction data, are inferred to be the main component of the upper 20 km of lithospheric crust in the Arabian Shield (Gettings et al., 1986), may have formed in the course of general N–S extension. Avigad and Gvirtzman (2009) proposed that removal/delamination of themantle lithosphere accounted for the coupled processes of magma emplacement and exhumation. As an additional factor, a significant rise of the overall geotherm may have been a consequence of mantle delamination. Removal of the previously thickened lithospheric mantle root potentially caused uplift of the northern ANS to elevations of more than 3 km, thus triggering rapid erosional unroofing and extension. Avigad and Gvirtzman (2009) argue that, between ∼630 and 600 Ma, crustal thinning by extension following mantle delamination resulted in the reduction of surface elevation by ∼2 km. Further surface lowering by another kilometre may have resulted from the restoration of the mantle lithosphere by thermal contraction. A discrepancy between information from the entire northern ANS and the model of Avigad and Gvirtzman (2009) relates to the presence of ∼650–620 Ma marine basins in the northeastern Arabian Shield, which indicate that the northeastern Arabian Shield was below sea level at ∼650–620 Ma and was later uplifted.

A particular type of “mantle delamination”, namely subduction, slab retreat and slab break-off may have been important for the northern ANS evolution. It is conceivable that retreat of arcs such as the Cadomian arc existed along the northern margin of the EAO and the larger Gondwana supercontinent. Existence of 583 Ma granulite facies rocks, ∼550 Ma orthogneiss, 530 Ma eclogites and metaclastic rocks with deposition ages between 600 and 550 Ma in the Menderes Massif of Turkey were attributed to closure of the Mozambique Ocean at the northern end of the East African Orogen (Koralay et al., 2012). Slabbreak-off at the northern peri-Gondwana margin may have induced emplacement of widespread lower crustal melts, their upward buoyant intrusion into supracrustal rocks, and the development of N–S extensional structures common throughout the northern ANS.

## 8 Summary of orogen forming events

**Fig. 15:**
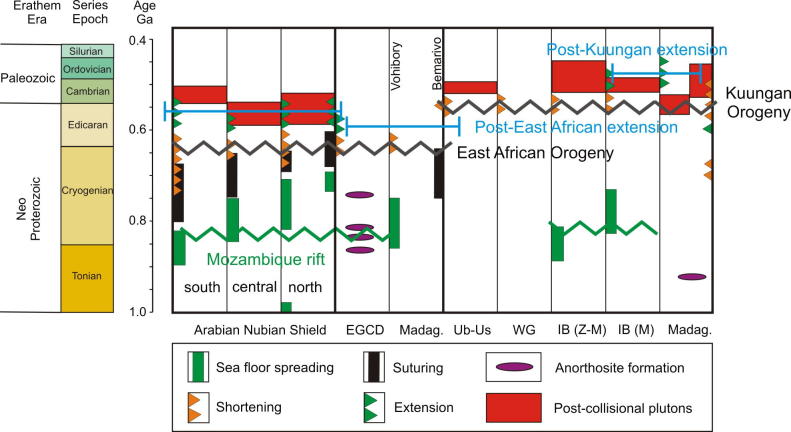
Condensed timetable of major geologic events in the East African Orogen. EGCD: Eastern Granulite–Cabo Delgado Nappe Complex; Mad: Madagascar; Ub–Us: Usagaran/Ubendian Belts; WG: Western Granulite Belt; IB (Z–M): Irumide Belt of Zambia and Malawi; IB (M): Irumide Belt of Mozambique.

Key factors, such as lithology as well as tectonic, metamorphic and magmatic events shown in Fig. 3 are, for simplicity, condensed in Fig. 15. Formation of the Mozambique Ocean initiated in the southern ANS as early as ∼900–850 Ma (some data from northern Sinai date back to 1030 Ma), but individual oceanic basins formed at different times. Some began to close soon after ocean floorformation. Approximately at the same time, oceanic crust formed within the Vohibory domain of southwestern Madagascar (Jöns and Schenk, 2008, Tucker et al., 2011), and small individual basins evolved within the Irumide Belt of Zambia, Malawi and Mozambique (John et al., 2003, Bjerkgard et al., 2009). Widespread emplacement of anorthositic and enderbitic melts within the EGCD of Tanzania and Mozambique but also in Madagascar is considered an expression of extending crust.

Prolonged convergence during the East African Orogeny and oblique collision of individual crustal units was initiated in the southern ANS and culminated at 640–620 Ma. At the same time (∼620 Ma), granulite-facies metamorphism occurred within the EGCD and the Vohibory domain, and early thrust-related fabrics evolved in centralMadagascar. Extensional structures postdating the East African Orogeny are widespread in the ANS and occur sporadically in the EGCD. Extension in the northern ANS, perhaps triggered by retreat of the Cadomian Arc was accompanied by voluminous intrusion of late tectonic granitoids and advectional heating of the entire crust.

The Kuungan Orogeny, variable in its metamorphic conditions, is limited to the southern EAO (Ubendian/Usagaran Belts, Irumide Belt, Western Granulite Belt, cratonic Madagascar). Kuungan metamorphism and deformation also affected the EGCD which achieved its final position within the nappe stack at ∼550 Ma. Post-Kuungan extension is most prominent in southern Madagascar and the Nampula Block of Mozambique. Here extension was accompanied by intrusion of late-tectonic granitoids and Ordovician high-temperature medium-pressure metamorphism dated 500–450 Ma (e.g. Thomaset al., 2010).

The ∼6000 km long composite East African mountain range was denuded by extension and erosion during late Neoproterozoic/Cambrian and Ordovician times. Enormous volumes of sediment would have been removed and deposited in adjacent basins. Remnants of such sediments are poorly preserved within central portions of the deeply eroded mountain range, but thick sequences of Early Paleozoic sandstone occur throughout northern Africa, Arabia and Antarctica, testifying to the past extent and size of this orogen (Jacobs and Thomas, 2004, Squire et al., 2006 and references therein).

## 9 Discussion on orogen styles

**Fig. 16:**
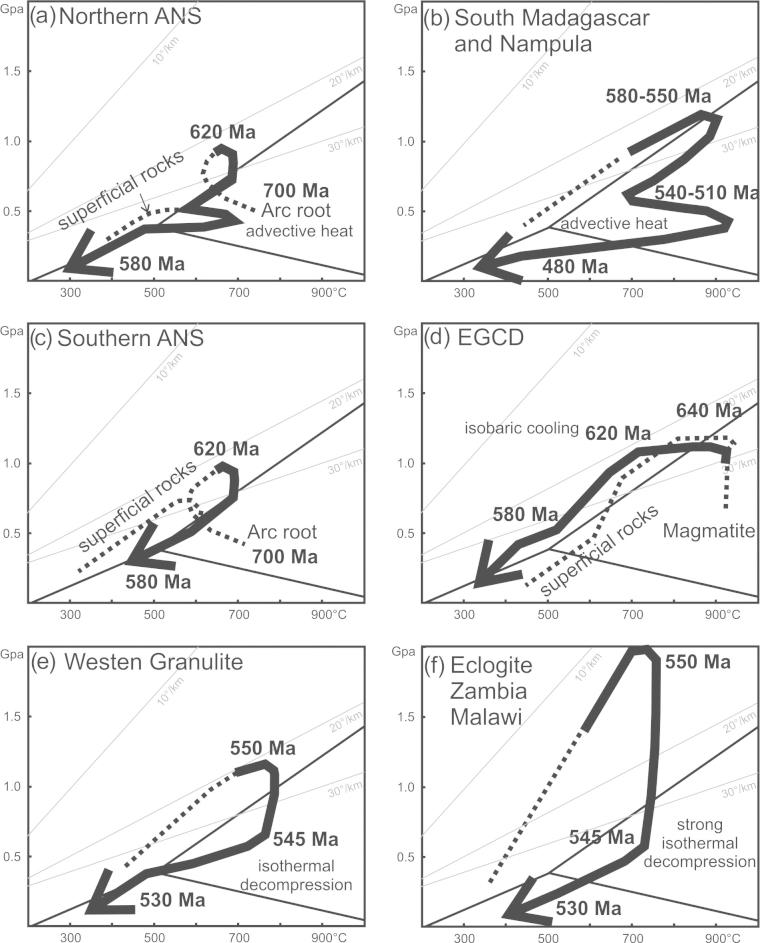
Summary sketch of pressure–temperature evolutionary paths typical for different domains within the EAO (data extracted from Fig. 7). Dotted lines are assumed courses of prograde paths. Approximate ages for individual segments are indicated. (a) Duality of evolutionary paths in the northern ANS. Arc roots cooled during thickening while superficial rocks were heated. Advective heating by combined rock exhumation and magma emplacement caused rise of the geotherm and β-shaped P–T loop. (b) A similar β-shaped P–T loop and rise of the geotherm resulted from Kuungan thickening followed by extension through delamination of the lithosphere in southern Madagascar and the Nampula Block. (c) The southern ANS experienced thickening by pure shear dominated transpression. P–T loops are similar to the northern ANS but advective heating due to extension is insignificant. (d) Slow isobaric cooling within the EGCD. (e and f) Clockwise P–T path in Western Granulites and Zambian/Malawian eclogites (see text for further explanation).

**Fig. 17:**
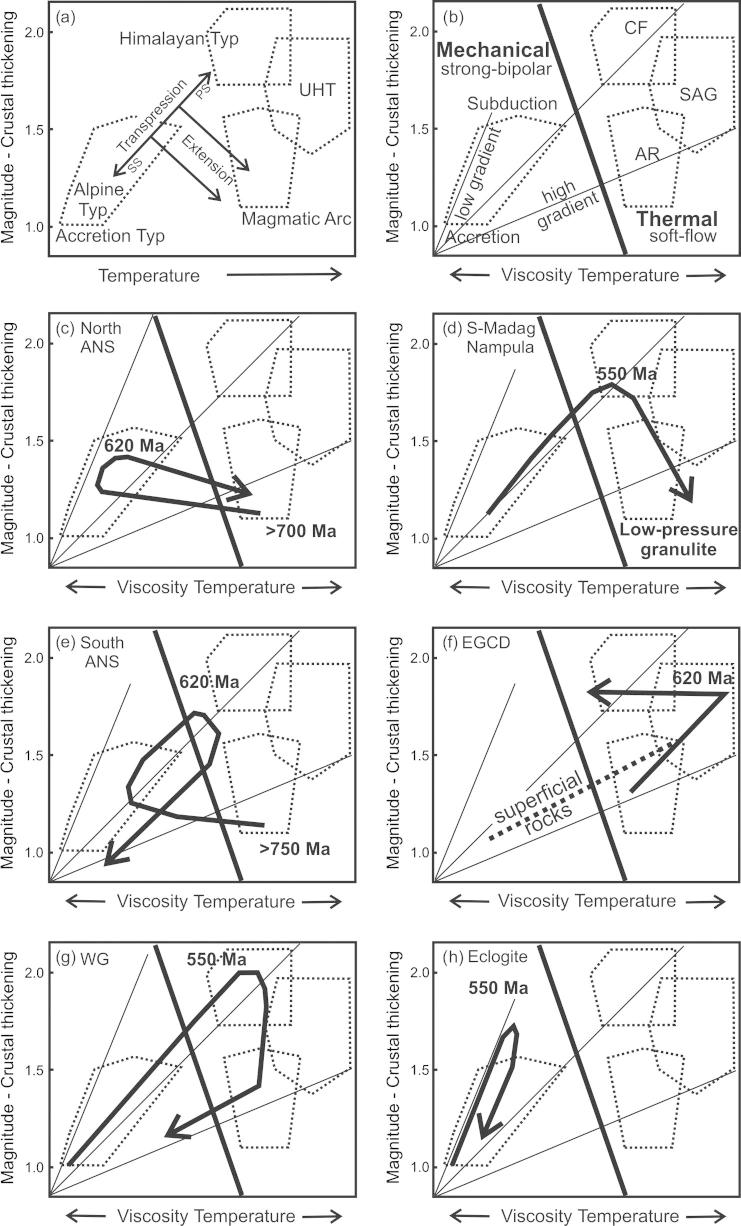
(a) Orogen magnitude – temperature diagrams with orogen types adopted from Beaumont et al. (2004). End members are small and cool accretion-type orogens and doubly-thickened Himalayan type orogens. Magmatic arcs and ultra-high-temperature orogens (UHT) evolved at higher geothermal gradients (thermal gradients are schematically shown in b–h). For transcurrent orogens the amount of thickening depends on the ratio between simple shear (ss) and pure shear (ps) components during deformation. Decay of orogens by extension and crustal thinning is accompanied by temperature rise. (b) Mechanical properties of orogens. Structures in cold orogens with high strength are determined by plate boundary forces. Typical features include subduction, and bipolar thrusting. Hot and soft orogens flow under their own weight (body force determined). Channel flow (CF), sagduction (SAG) and syn-magmatic deformation within roots of arcs (AR) characterize this setting. (c) Northern ANS evolution. Hot arc magmatics with high thermal gradient cooled during thickening and were incorporated into thin-skinned thrusting (accretionary type). Subsequently the orogen received advective heat during its decay. (d) South Madagascar and Nampula Block evolution. The orogen was growing during Kuungan orogeny (Himalayan type) and the geotherm was rising during orogen decay. Final cooling is not shown in c and d. (e) The southern ANS experienced thickening by pure shear dominated wrench tectonics and, by contrast to the northern ANS, cooled during orogen decay. (f) The EGCD was thickened by sagduction during the East African Orogeny, rocks remained at depth and cooled isobarically. (g) The Western Granulites (WG) were doubly thickened (Himalayan type) and subsequently rapidly exhumed. (h) Zambian and Malawian eclogites were incorporated into a subduction channel (Alpine type) (see text for further information).

To stimulate discussion, we summarize available information for key parameters that control orogenic styles. (1) Salientproperties of older crustal segments such as age and thermal state are thought to predetermine conditions at the onset of an orogeny. (2) Relative plate motion determines the overall tectonic style within orogens, here grouped into collision, transcurrent and extensional orogenic phases. (3) Internal strength and rheology stratification within plates and along plate boundaries are considered the result of the heat budget during growth and decay of orogens. The progressive thermal evolution is best displayed by use of pressure–temperature–time diagrams, here abstracted from available P–T path (Fig. 7, Fig. 16). In order to link pressure and temperature data with thickening and thinning events we have slightly modified a diagram from Beaumont et al. (2004) to define orogen styles (Fig.17). In this diagram paramount orogen types can be defined and linked with mechanical properties of the orogen. Simplifications have been made while recognizing that distinct portions within orogens may have experienced variable histories. Unless otherwise indicated we display evolutionary trends recorded from the middle to lower crust.

### 9.1 Pre-orogenic properties of East African orogen segments

Neoproterozoic/early Paleozoic tectonic events operated under a variety of different conditions. Orogeny within the ANS affected extending crust and spreading oceanic lithosphere. Soon after break-up of Rodinia and opening of the Mozambique Ocean, arc- back-arc basin systems formed, yielding a collection of different terranes. Protracted crust-forming events covered ∼250–300 million years, continuously forming juvenile crust andindividual terranes. We consider that the ANS lithosphere pre-dating Gondwana collision was hot because of continuous magma generation.

Late Neoproterozoic orogeny within the EGCD was characterized by inversion tectonics along a formerly extending crust. The orogenic lag time in the EGCD indicated by the interval between deposition of marbles that might represent a passive continental margin sequence (800–660 Ma: Melezhik et al., 2008) and peak metamorphism (655–615 Ma: e.g., Möller et al., 2000) may be as little as 5 Ma or as much as 175 Ma. This interval and the presence of voluminous lower crustal melts, such as anorthosite and enderbite formed between 900 and 700 Ma (e.g., Tenczer et al., 2006), imply thatthe EGCD crust was soft.

Reworked pre-Neoproterozoic crustal fragments within and on the margins of the EAO share a common feature in that Neoproterozoic/early Paleozoic orogenies overprinted heterogeneous and previously thickened domains. Preservation of pre-Neoproterozoic orogenic cycles with comparatively few superimposed Neoproterozoic rocks define them as large continental masses away from the immediate Mozambique Ocean margins. Time lags between the pre-Neoproterozoic and Neoproterozoic orogenic events range from ∼2000 Ma (cratonic Africa, Western Granulite Belt, Archean Madagascar), ∼1200–1050 Ma (Ubendian and Usagaran Belts and Itremo-Ikalamavony domain of Madagascar) to ∼470–400 Ma (Irumide Belts of Zambia Malawi and Mozambique). Large lag times together with a crust that was thickened and eroded by preceding orogenies identify the reworked MB as a thick and coolorogen.

### 9.2 Orogen evolution trends

Orogen evolution trends are best recognized when abstracted pressure–temperature paths (Fig. 16) are jointly inspected with magnitude-temperature diagrams (Fig. 17). In the latter, dimensionless orogen magnitude or crustal thickening is plotted against the overall heat an orogen possesses. In the absence of comprehensive data, the magnitude of crustal thickness has to be estimated. One measure of crustal thickness is the Moho depth, but where this is not available we estimate crustal thickness by assessing metamorphic conditions and the geodynamic settings of the crustal regions under consideration. For the heat budget, the temperature at Moho depth would be most useful measure, but again this is not everywhereavailable. Pressure–temperature data and inferred thermal gradients, together with plausible crust-forming scenarios, are used to define overall dimensionless thermal regimes. Typical orogen types such as Alpine-type, Himalayan-type, magmatic arcs, and ultra-high temperature orogens are indicated in Fig. 17a. For transcurrent orogens we note that simple shear-dominated transpression covers the field of small and cold orogens due to dominant lateral displacement. Pure shear dominated transpression may accommodate significant thickening, similar to collisional-type orogens. Fig. 17b also provides insight into prevalent mechanical properties during orogen evolution. Small and cold settings are mechanically determined, forming typically bipolar orogens. Hot and large orogens with their low-viscosity lower crust are thermally determined and tend to flow under their ownweight.

The northernmost ANS and the southern most EAO in southern Madagascar and Mozambique (Nampula) exhibit high thermal gradient during their decay (Fig. 17c and d). This is evident from abstracted pressure–temperature curves that display β-shaped loops (Fig. 16a and b). A significant rise in the overall geothermal gradient by extension is, in both orogens, explained by thinning of the mantle lithosphere. The plate tectonic frame is an Ordovician retreating southern EAO margin (Jacobs and Thomas, 2004) and probably the Cambrian retreating Cadomian and/or Al-Amar arcs (Johnson et al., 2011) in northern Arabia. The northern ANS evolved from initially hot arcs and the thermal gradient diminished during strike-slip accretion (Fig. 17c). Late tectonic heating by extension and exhumation of hot rock from deep in the crust significantly lowered the viscosity of the melt-infiltrated lower crust and allowed gneiss domes to ascent buoyantly. Blueschist- or eclogite-facies metamorphism is absent within the northern, thin-skinned ANS, which suggests that obduction was more significant than subduction in formation of the northern ANS. Although southern Madagascar, the Nampula Block, and the northern ANS share similar extension-related heating, pre-extension thickening was greater in the southern EAO than the north (Fig. 17c and d). The southernmost EAO experienced significant thickening at ∼550 Ma, evidenced by high-temperature, high-pressure granulite formation and was later subjected to extension evidenced by high-temperature, low-pressure granulite formation.

The southernANS is considered to be a pure shear-dominated transpressional orogen with significant and prolonged thickening. It evolved from initially hot arcs older than 750 Ma. During accretion, rocks joined with ophiolite suites that record low thermal gradients (Fig. 16c). Pure shear transpression was followed by exhumation without significant rise of temperature (Figs. 16c and 17e), a different exhumation scenario to that found in the northern ANS. Gravitational collapse evidently occurred at confined plate boundaries without thinning of the subcrustal mantle lithosphere (Tsige and Abdelsalam, 2005).

An anticlockwise pressure–temperature loop characterizes the EGCD nappe complex, which has the character of a hot to ultra-hot orogen(Figs. 16d and 17f). Enderbite and anorthosite magmas formed at formerly extended lower crust (short orogenic lag time) and joined with superficial rocks by sagduction. The melt-weakened, extremely low-viscosity lower crust was continuously thickening by thermally induced orogen flow. Slow, isobaric cooling of the lower crust in the EGCD is valid if horizontal flow was faster than thermal equilibrium in the rocks (Fig. 10). The Western Granulite Belt has, by contrast, an inconspicuous pressure–temperature trend. The belt experienced crustal thickening of formerly stiff and cold crustal fragments (large orogenic lag time) to form a thick-skinned, Himalayan type orogen (Fig. 17g). Isothermal decompression fabrics and rise of the geothermal gradient (Fig. 17e) indicate that the crust underwent subsequent exhumation, probably by combined thrusting and erosion. The other Kuungan collisional belts such as central Madagascar and northern Mozambique may have formed in a similar fashion.

The Irumide of Zambia and Malawi and the Ubendian Belts differ in that they contain Kuungan high-pressure medium-temperature eclogite boudins enclosed within lower-grade rocks (Fig. 16f). This suggests localized, mechanically induced, subduction of oceanic remnants at a low geothermal gradient. The fact that the northern Irumide and the Ubendian Belts never experienced areally distributed Neoproterozoic to Cambrian metamorphism disallows the existence of extensively thickened crust on a regional scale. An absence of overthickened crust is in agreement with descriptions that define the Irumide Belt as a doubly-vergent transcurrentthin- to thick-skinned orogen (Dirks et al., 1999, Ring et al., 2002). This belt resembles bipolar Alpine-type orogens (Fig. 17h). Isothermal decompression in this case is in agreement with localized eclogite exhumation within a narrow subduction channel.
